# Bipedalism and brain expansion explain human handedness

**DOI:** 10.1371/journal.pbio.3003771

**Published:** 2026-04-27

**Authors:** Thomas A. Püschel, Rachel M. Hurwitz, Chris Venditti

**Affiliations:** 1 Institute of Human Sciences, School of Anthropology and Museum Ethnography, University of Oxford, Oxford, United Kingdom; 2 School of Biological Sciences, University of Reading, Reading, United Kingdom; University of St Andrews, UNITED KINGDOM OF GREAT BRITAIN AND NORTHERN IRELAND

## Abstract

Humans exhibit a striking and near-universal population-level right-hand preference, an evolutionary singularity unmatched among primates. Despite its pervasiveness, the origins of this lateralization remain poorly understood. Here, we combine phylogenetic comparative methods with meta-analysis to investigate manual lateralization across 41 anthropoid species (*n* = 2,025), testing longstanding eco-evolutionary hypotheses for handedness direction (mean handedness index, MHI) and strength (mean absolute handedness index, MABSHI). Our models reveal significant phylogenetic signal for both traits and identify *Homo sapiens* as an evolutionary outlier, exhibiting exceptional rightward bias and strength relative to phylogenetic expectations. However, this outlier status disappears when brain size (endocranial volume) and intermembral index are included, suggesting these factors are central to the emergence of human handedness. We also show that high MABSHI evolved early in hominin evolution, while MHI increased to unparalleled levels with the appearance of the genus *Homo*. Our findings identify bipedalism and neuroanatomical expansion as likely key drivers of uniquely human lateralization, while also revealing broader ecological patterns shaping handedness across primates. This work provides a framework for disentangling human-specific adaptations from general primate trends in the evolution of behavioral asymmetries.

## Abstract 2

Why do humans exhibit a striking and near-universal population-level right-hand preference? This study of 41 anthropoid species reveals Homo sapiens as an evolutionary outlier and identifies bipedalism and brain expansion as likely key drivers of human lateralization.

## Introduction

In all human cultures across every corner of the globe about 90% of people favor their right hand [1–4]. Based on archeological evidence, some have argued that this has been true since the Neolithic [5], whilst others contend that it has been constant through the entire *Homo* lineage [6–11]. Furthermore, individual human's strong manual lateralization means that ambiguous hand preferences, or forms of ambidexterity, are extremely rare, which appears unusual when compared to other primate species [11–13]. Still, some level of directional manual lateralization is present in sub-populations of various primate species, but the level and consistency of handedness in humans is unmatched, and despite much interest, still represents an unexplained evolutionary singularity [4,11,12,14–16].

**Fig 1:**
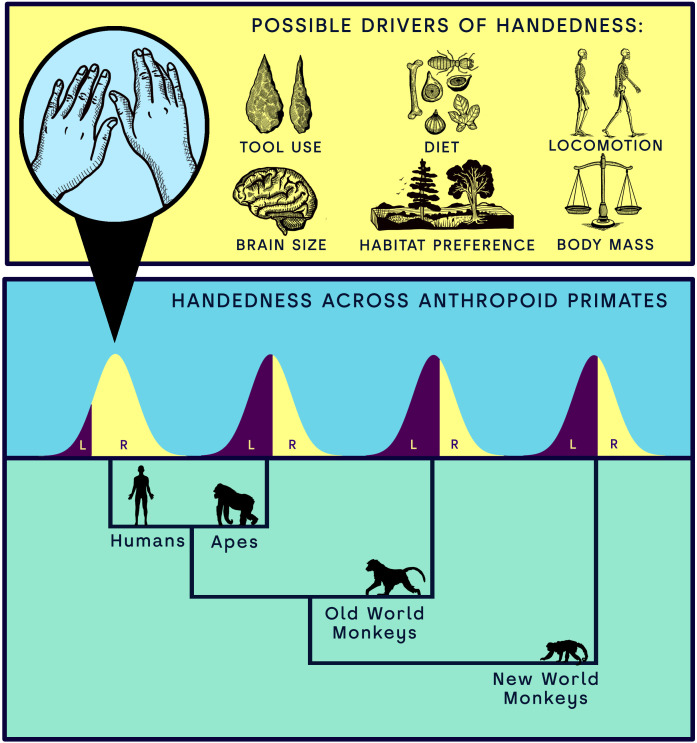
Drivers often proposed to explain the unique pattern of human handedness direction. Humans show an unparalleled level of rightwards handedness bias as compared to other anthropoid clades.

At a mechanistic level, the neurological basis of handedness is known to be rooted in specialized brain regions [17,18] showing associations with hemispheric specialization for some higher cognitive functions [19,20]. Genetic studies further indicate that handedness is a highly polygenic trait, influenced by several loci alongside complex epigenetic interactions, though the full complexity of genetics on handedness is still unclear [21]. At the ontogenetic level, hand preference appears to begin developing in utero, as suggested by early unilateral arm movements observed in embryos [22,23], although the interpretation of these early movements remains uncertain due to small sample sizes. Hand preference then continues to develop and consolidate throughout infancy and adolescence [24], potentially influenced by early life factors [25], and ultimately resulting in lasting changes in bone shape and density [26]. Still, at the evolutionary level, the origins and persistence of population-level handedness remain enigmatic [4,27–31]. Several hypotheses have been advanced to account for its emergence and maintenance across primates, often linking manual asymmetry to ecological, anatomical, or cognitive pressures (Fig 1; S1 Table). However, many of these explanations have only been posited in ambiguous and descriptive terms making them difficult to test and hampering progress in the area. This has resulted in many conflicting hypotheses concurrently existing in the literature without any clear tests of their applicability.

Handedness in non-human primates has been comparatively understudied relative to humans, especially longitudinally, as well as across species. Most frequently, primate handedness is determined via observation of repeated tasks in the wild (i.e., nut-cracking in chimpanzees) or using standardized tests in captive environments (i.e., simple reach tasks or the tube task). As a result, there is limited evidence of a right-hand bias in chimpanzees, and evidence that, like with humans, this is associated with brain asymmetries [13,32]. However, on the whole these studies tend to have small sample sizes, the primates’ hand usage may vary between task type (i.e., when gesturing versus reaching versus using a tool) and concerns have been raised that individuals in the latter studies may be influenced towards right-handedness by their human carers [33,34]. This has created significant debate regarding the extent to which handedness can be quantified in primates, especially at the population-level [34,35]. A recent meta-analysis found that, unlike humans, non-human anthropoids do not show population-level handedness but do exhibit strong manual preferences during bimanual tasks [36]. A phylogenetic study confirmed that human right-handedness is an extreme case, with population-level handedness being rare in non-human primates [15]. The latter also found no strong link between manual lateralization and factors like tool use, substrate preference, or brain size, but noted that terrestrial species had weaker hand preferences than arboreal ones.

No combined phylogenetic comparative and meta-analytical method has been used to study anthropoid handedness, leaving key eco-evolutionary hypotheses untested. This approach offers several advantages, including better identification of variation drivers, improved control of data biases, and more accurate phylogenetic conclusions [37]. Although comparative and meta-analytical methods are rarely combined in evolutionary studies, they reduce sampling errors, account for inter-study variability, and help address research biases, such as the overrepresentation of certain charismatic species (e.g., chimpanzees) [38–40]. This study integrates both methods to assess hypotheses on primate and human handedness.

**Fig 2:**
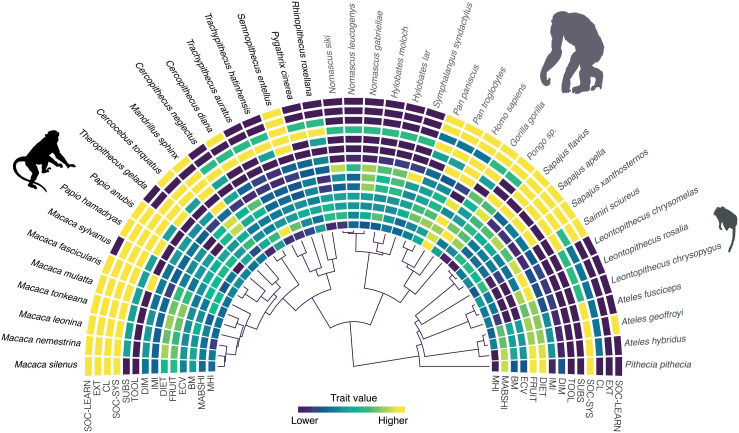
Traits analyzed in the present study and maximum credibility clade tree for the analyzed anthropoid species. The acronyms correspond to: MHI, mean handedness index; MABSHI, mean absolute handedness index; BM, body mass [kg]; ECV, endocranial volume [cm^3^]; FRUIT, percentage of fruits in diet; DIET, percentage of fruits and animals in diet; IMI, intermembral index; DIM, body mass sexual dimorphism; TOOL, tool use [0 = absence, 1 = presence); SUBS, substrate preference [0 = arboreal, 1=both, 2 = terrestrial]; SOC-SYS, social system [0 = solitary, 1 = pair, 2 = group]; CL, intra-sexual competition levels sensu [1–4,70]; EXT, extractive foraging [0 = absence, 1 = presence]; social learning sensu [71] [0 = absence, 1 = presence]. Traits are color coded from lower to higher values. In the case of the discrete traits, colors also go from lower to higher values based on the categorization provided in this legend. Cercopithecoidea, Hominoidea and Platyrrhini are represented by different silhouettes, as well as colors. The data underlying this Figure can be found in S2 Data.

We applied Bayesian Phylogenetic Comparative Meta-Analytical methods to assess handedness patterns across anthropoids. Handedness was evaluated based on two facets: direction (mean handedness index, MHI) and strength (mean absolute handedness index, MABSHI). Our data include a recent meta-analysis [36] and recent experimental data [15] (see Materials and methods), resulting in a standardized dataset of 2,025 individuals across 41 anthropoid species. To test the influence of phylogeny and previously proposed hypotheses on handedness in humans and other primates, we compiled relevant covariates (e.g., body mass, brain size, tool use, intrasexual competition) from various literature sources (Fig 2; S1 Data; see Materials and methods). Multiple lines of evidence suggest that *Homo sapiens* is an evolutionary outlier in handedness, showing extreme rightward bias. We therefore conduct our hypothesis testing including and excluding *H. sapiens.* In addition, to explicitly assess our potential evolutionary distinctiveness, we use a “Phylogenetic Outlier” test [41]. We also use our phylogenetic meta-analytical models to predict MHI and MABSHI values for extinct hominin species, based on their phylogenetic positions and associated predictor variables.

## Results

### Directionality of handedness

Contrary to previous studies, we demonstrate that MHI shows significant phylogenetic signal (*h*^2^ = 0.48). This discrepancy likely stems from our approach, which adjusts for sampling error rather than relying on species means. There is no directional preference in MHI value across anthropoid species (MHI = −0.03, 95% CI: −0.32, 0.22) (S1 Fig). As expected, the highest MHI of 0.76 was found in *H. sapiens*, and although population-level handedness direction-biases were uncommon, other species including *Pan troglodytes*, *Gorilla gorilla*, and *Cercopithecus diana* showed a weak-moderate rightward preference (MHI > 0.17) (S1 Fig). Interestingly, there were more species displaying a stronger leftwards bias (MHI < −0.3) (e.g., *Pithecia pithecia*, *Ateles hybridus*, *Sapajus flavius*, *Pongo* sp., *Rhinopithecus roxellana*, *Cercopithecus neglectus*) (S1 Fig). However, it is important to keep in mind that in most cases, confidence intervals overlap with zero, indicating higher variability. In fact, *Pongo* sp. (MHI = −0.32) and *Rhinopithecus roxellana* (MHI = −0.32) are the only two taxa that show credible leftward preference, whereas humans are the only species showing a credible rightward preference.

When testing all the hypotheses listed in S1 Table using MHI as the dependent variable, we found that no single hypothesis performed meaningfully better than any other, which was true both with and without humans (S2 Table). Interestingly, the inclusion of humans in the model routinely changed the significance of predictors in the model highlighting our species as an evolutionary outlier (see S1 Section). This was the case in every hypothesis that included brain size (measured by endocranial volume, ECV) and locomotor adaptations (measured by the intermembral index, IMI). The human IMI is extremely low, 72, reflecting that our hindlimbs (legs) are significantly longer than the forelimbs (arms), which is a key adaptation for bipedal locomotion. This provides evidence that brain size and bipedal location have driven our exceptional MHI. After excluding *H. sapiens,* only “social system,” when testing the *Tool-use and social organisation hypothesis* (TU-SH-SSH) hypothesis, is a credible predictor (which is explained by the orangutan) and body mass in the *Substrate preference, social organisation, bipedalism and tool-use hypothesis* (SP-SS-B-TUH).

**Fig 3:**
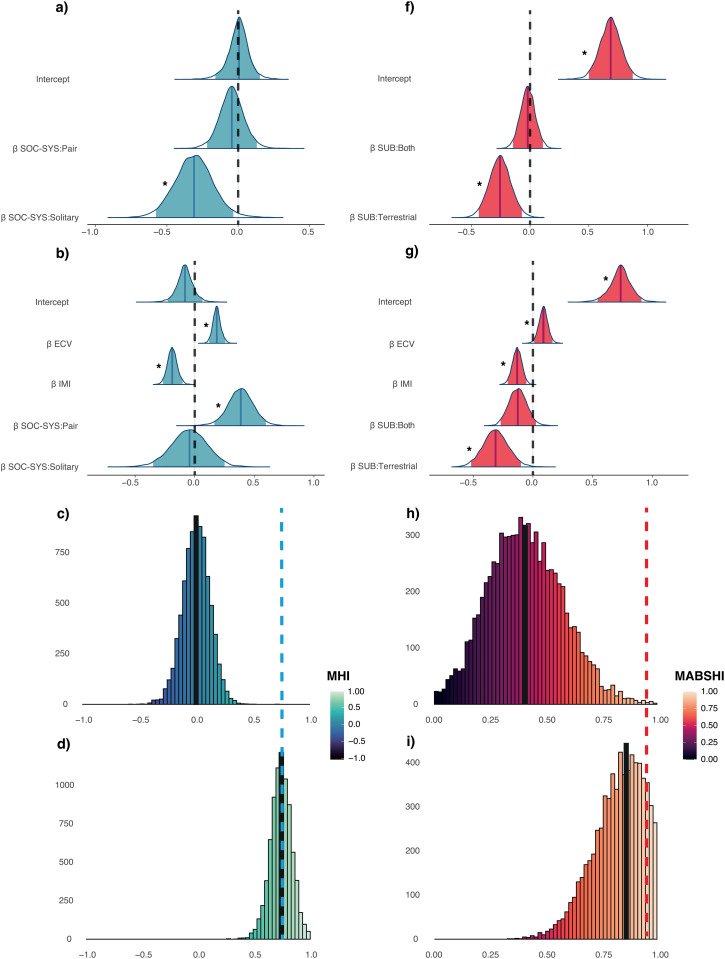
Coefficient estimate distributions for the reduced models and phylogenetic outlier tests for MHI and MABSH. The upper for panels show the coefficient estimate distributions for the reduced models: **(a)** MHI excluding *Homo sapiens*, **(b)** MHI including *H. sapiens*, **(f)** MABSHI excluding *H. sapiens*, and **(g)** MABSHI including *H. sapiens*. Effects are considered credible when the 95% credibility intervals (light shaded areas) do not overlap with zero (dotted line). The median for each coefficient distribution is represented by darker solid lines. Credible predictors are marked with asterisks. The lower four panels present the posterior distribution of predictions for MHI and MABSHI for a new *Homo sapiens* observation. It illustrates these predictions based on the reduced models: **(c)** MHI excluding humans, **(d)** MHI including humans, **(h)** MABSHI excluding humans, and **(i)** MABSHI including humans. All predictions are marginalized over meta-analytical and non-phylogenetic species random effects. The data underlying this Figure can be found in S2 Data.

To better evaluate the role of the predictors identified as credible for MHI, we created two new models, following the same modeling procedures as described before. Each model used only the predictors that were deemed relevant across all tested hypotheses. The first model, which excluded *H. sapiens*, included IMI, body mass and social system as covariates. The second model included *H. sapiens*, and featured diet, ECV, IMI, tool use, substrate preference, body mass, and social system as fixed effects. We then applied a model reduction procedure, iteratively removing predictors whose posterior distributions showed no credible effects. Social system: pair was the only predictor that retained a credible effect in the first model excluding *H. sapiens* after model reduction (Fig 3a). The *R*^2^ value for this model was 0.26, indicating that social system only explains a limited amount of the variation in MHI patterns. The second model resulted in only three predictors retaining credible effects, ECV, IMI, and social system: pair after model reduction (Fig 3b). This model displayed a *R*^2^ of 0.42, thus showcasing the relevance of these three covariates.

These results suggest that *H. sapiens* are an outlier relative to the general primate trend. Therefore, we explicitly tested the status of humans as an evolutionary singularity using a phylogenetic outlier test (methods). Fig 3c displays both the observed MHI value for *H. sapiens* and the posterior distribution of MHI values derived from a model in which humans were excluded. The MHI prediction for the model using the predictor with a credible effect when excluding humans is 0.0, whereas the observed value is 0.76, highlighting the exceptional nature of human handedness direction relative to phylogenetic expectations. This divergence implies that strong selective pressures would have been necessary to produce such a striking difference. Interestingly, *H. sapiens*’ position as an outlier is no longer evident when ECV, IMI, and social system: pair are included in the model, as the obtained MHI value (0.74) is almost identical to the observed one (0.76) (Fig 3d). This suggests that selection pressures associated with these traits may explain the uniquely high MHI value observed in humans.

**Fig 4:**
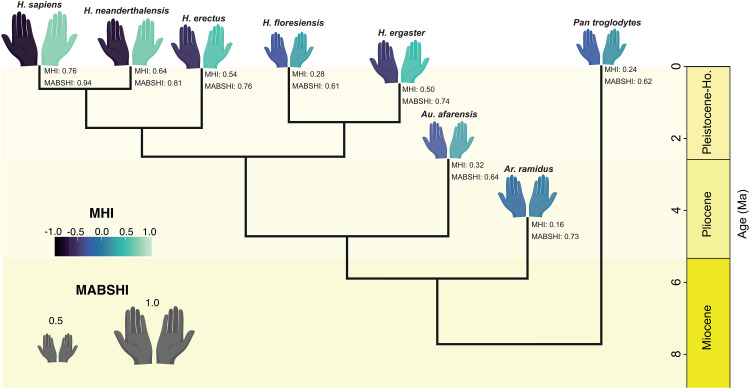
Predicted handedness in hominin species based on the reduced models including *Homo sapiens.* Right-hand colors represent the predicted magnitude of MHI; left-hand colors show the corresponding negative values (i.e., greater color differences between hands reflect stronger handedness direction bias). Hand size is proportional to handedness strength, MABSHI. The phylogeny is the maximum a posteriori tree from ref [34]. Only hominin species with complete relevant covariate data were included in the analysis and are shown here. The handedness values for *Pan troglodytes* and *Homo sapiens* correspond to observed data. The data underlying this Figure can be found in S2 Data.

When using the reduced model including humans to predict hominin MHI values we found a clear trend of increasing MHI from older to more recent hominin species (Fig 4): *Ardipithecus ramidus* (0.16), *Australopithecus afarensis* (0.32), *Homo ergaster* (0.50), *Homo erectus* (0.54), and *Homo neanderthalensis* (0.64). The notable exception is *Homo floresiensis*, which exhibits a comparatively weaker handedness directionality (MHI = 0.28). These results suggest that earlier hominins displayed weaker or less consistent hand preferences, whereas more recent species demonstrated stronger and more consistent lateralization.

### Strength of handedness

MABSHI showed a stronger phylogenetic signal (*h*^2^ = 0.66) than MHI, though lower than previously reported [15]. Again, this difference likely stems from our meta-analytic and multilevel approach, which allowed us to include multiple measurements per species and apply differential weighting based on study standard errors. The mean MABSHI for anthropoids was credibly above zero (MABSHI = 0.66, 95% CI: 0.44, 0.91; S2 Fig), indicating strong individual hand preferences across the clade, despite a lack of directional bias in most species. As expected, *H. sapiens* showed one of the highest MABSHI values (0.94), though East Javan langurs (*Trachypithecus auratus*) exhibited the strongest handedness strength among anthropoids (MABSHI = 0.98). Overall, humans and primarily arboreal species, such as langurs (*Trachypithecus*) and spider monkeys (*Ateles*), displayed the highest MABSHI values, while predominantly terrestrial species, such as geladas (*Theropithecus*) and baboons (*Papio*), showed weaker hand preferences (S2 Fig).

Similar to MHI, testing all hypotheses listed in S1 Table with MABSHI as the dependent variable revealed no hypothesis performed significantly better than others, regardless of whether humans were included (see S2 Section; S3 Table). The exclusion of humans did, however, alter the significance of some predictors. As with MHI, predictors such as ECV and IMI showed credible effects when humans were included. For MABSHI, body mass displayed a credible association across multiple hypotheses. When we excluded humans from our dataset, we observed that most of the tested hypotheses retained substrate:terrestrial as the only predictor with a credible effect. The only other covariate showing a credible effect corresponded to body mass sexual dimorphism under the fighting hypothesis, whose posterior interval did not include zero.

To further assess the relevance of the predictors that showed credible effects for MABSHI, we ran two new models that exclusively considered all the predictors that were found to be relevant in all the tested hypotheses. The same modeling steps used in the previous models were followed. Therefore, we tested one model excluding *H. sapiens* that had as covariates substrate preference and body mass sexual dimorphism, and another including humans that had as fixed effects diet, ECV, IMI, substrate, body mass and body mass sexual dimorphism, and applied an iterative model reduction approach to only keep those predictors that were credibly associated. Substrate:terrestrial was the only credible predictor in the first model without *H. sapiens* (Fig 3f). The *R*^2^ value for this model was 0.79, which indicates again the crucial role that locomotor differences seem to play in MABSHI patterns. The second model that included *H. sapiens*, resulted in only three credible predictors after model reduction, ECV, IMI and substrate:terrestrial (Fig 3g). The *R*^2^ for this model was 0.8, which indicates the key role that locomotion and brain size plays in MABSHI pattern.

As with MHI, we explicitly tested the status of *H. sapiens* as an evolutionary singularity for MABSHI using a phylogenetic outlier test (see Materials and methods). Fig 3h shows both the observed MABSHI value for *H. sapiens* and the posterior distribution of MABSHI values predicted by a model in which humans were excluded. The phylogenetic prediction for MABSHI is 0.43, whereas the observed value is 0.94, further highlighting the exceptional nature of human MABSHI relative to phylogenetic expectations. However, when IMI is included in the model, *H. sapiens* is no longer identified as an outlier (MABSHI: 0.86; Fig 3i). This suggests that IMI is a critical factor in explaining the distinctiveness of human handedness as captured by MABSHI.

Using the reduced model to predict hominin MABSHI values, we observed consistently high handedness strength across species: *Ar. ramid*us (0.73), *Au. afarensis* (0.64), *H. ergaster* (0.74), *H. erectus* (0.76), *H. floresiensis* (0.61), and *H. neanderthalensis* (0.81) (Fig 4). These results suggest that handedness strength has remained consistently high since the last common ancestor of hominins and panins.

## Discussion

We present a comprehensive phylogenetic comparative meta-analysis of handedness in anthropoids that accounts for shared evolutionary history, effect variation across multiple sources, and potential publication bias, thus addressing many previous limitations. By testing multiple hypotheses about the evolution of handedness simultaneously, we offer new insights into the role of evolutionary selection in shaping both the direction (MHI) and strength (MABSHI) of manual lateralization. Our results should be understood with the caveats which come from collating data from multiple sources, and that handedness as determined by standardized protocols may not fully reflect the complexity of primate hand use and manual preferences in the wild [4,34,35]. Nevertheless, the meta-analytical framework applied here mitigates to some extent data heterogeneity by modeling between-study variance and applying inverse-variance weighting, thereby giving greater influence to more precise estimates [42]. Thus, by integrating behavioral data using a meta-analytical and phylogenetic comparative approach [37], we show that humans occupy an extreme position within the primate order with respect to both the strength and direction of handedness.

Our results highlight significant phylogenetic signal in both MHI and MABSHI, indicating that handedness traits have evolved with descent along the branches of the primate phylogenetic tree. However, the data do not fully support any single existing hypothesis, with most predictors only showing significance when *H. sapiens* is included. This suggests that many hypotheses regarding handedness may be overly anthropocentric. As such, future research should seek to clearly distinguish between human-specific and broader primate explanations for manual lateralization as it extends to hand preferences, manipulative abilities, and communicative influence [43].

A striking finding of this study is the identification of *H. sapiens* as an outstanding outlier in MABSHI but especially MHI. Humans display a pronounced right-handed bias (MHI = 0.76), which contrasts sharply with the phylogenetic prediction of the reduced model excluding humans (MHI = 0.0) (Fig 3c). Likewise, humans show extreme handedness strength (MABSHI = 0.94), with MABSHI values near the highest observed among anthropoids, although some arboreal species, such as spider monkeys and langurs, also exhibit high levels of lateralization strength. These results strongly support the hypothesis that human exceptionalism in handedness is likely due to to strong, human-specific selective pressures.

When considering the broader primate phylogeny, several predictors show credible effects for both MHI and MABSHI include factors such as IMI, ECV, diet, and social system. However, when *H. sapiens* is excluded from the analysis, only a limited number of predictors retain credible effects, with most posterior intervals overlapping zero. Notably, IMI, substrate use emerged as key factors. Lower IMI (indicative of more quadrupedal or leaping locomotion) correlate with leftward bias in MHI, and arboreal species generally exhibit higher MABSHI values. Arboreal species, needing flexible stabilization and precise movements in the canopy, often use one hand for support (left or right being arbitrary) and benefit from higher MABSHI for enhanced motor control. Terrestrial species, facing comparatively simpler and more uniform substrates, exhibit less complex locomotor patterns, requiring less specialized and lateralized motor skills owing to more repetitive and straightforward movements. This highlights the importance of locomotor and ecological factors in shaping handedness across non-human primates, whereas our large brain size emerges as an important link to human’s strong directionality in handedness. These results imply that our unusual gait was the main initial driver of our exceptional handedness strength with our large brain more linked to the directionality. In humans, the evolution of bipedalism and the subsequent freeing of the hands may have intensified selective pressures for stronger hand preferences. This finding aligns with previous research linking bipedal posture to the evolution of more pronounced handedness in fossil hominins, further suggesting that the evolutionary trajectory of human handedness is rooted in our unique locomotor adaptations [44–47].

Our predictions for other hominin species using our reduced models provide a more precise temporal perspective on the evolution of handedness direction and strength in human evolution (Fig 4). Our results indicate consistently high handedness strength (MABSHI) among hominins from early on in the lineage. Although hominins became increasingly terrestrial throughout their evolution [48], a locomotor behavior that reduces MABSHI among other anthropoids, the persistence of strong handedness strength is likely explained by our unique mode of locomotion—bipedalism—which freed the upper limbs and enabled specialized manual behaviors [49], maintaining selective pressure for lateralized hand use. This consistently high MABSHI level may also offer some support for those views that consider that that adaptations for bipedalism arose in an arboreal context [50], as more arboreal species tend to show higher handedness strength values.

In contrast, handedness direction (MHI) follows a different evolutionary pattern (Fig 4). Early hominins such as *Ar. ramidus* and *Au. afarensis* exhibit MHI values relatively similar to other great apes. It is with the emergence of the genus *Homo*, and particularly the onset of significant encephalization, that we observe a marked increase in MHI values, reaching levels that are highly unusual among anthropoids. This pattern suggests a novel link between the evolution of directional handedness and increasing brain size [51], highlighting a link between these two human evolutionary hallmarks. In this context, the emergence of pronounced right-handedness bias in humans may be viewed as part of a broader suite of neurological and behavioral specializations tied to the unique cognitive trajectory of our lineage. Importantly, this connection between encephalization and handedness direction has not been demonstrated in such a temporally resolved, phylogenetically informed framework before, offering new insights into the deep evolutionary roots of human lateralization.

The intriguing result for *H. floresiensis*, which shows relatively low MHI despite its placement within the genus *Homo*, may be explained by its unusual combination of small brain size and a unique locomotor repertoire blending bipedalism with arboreality. While the pelvis and lower limbs of *H. floresiensis* exhibit clear adaptations for upright walking, features such as long feet, an elongated forefoot, and curved phalanges suggest a locomotor pattern more similar to *Australopithecus*, including climbing behaviors [52]. This unique morphology may have reduced handedness direction evolution in this lineage. However, further analyses are necessary to confirm this hypothesis.

While only studies that employed the standardized “tube task” to calculate MHI and MABSHI values were included, and analyses were designed to minimize individual study bias and account for sample heterogeneity, some limitations must be acknowledged. Direct comparisons between human and non-human primate handedness are inherently constrained by differences in data collection, including potential variation in exact tasks, trial numbers, and sample sizes across species, as well as by the fact that human handedness data are often derived from questionnaires or observations of adults with strongly established, and likely culturally reinforced, manual preferences [16,43]. Importantly, handedness is also context-dependent: in humans, strong right-handedness is most consistently observed in goal-directed object manipulation, whereas its strength may weaken or even reverse in other behavioral contexts (e.g., repetitive gross motor or social actions) [43,53–56]. This further complicates cross-species comparisons, as different experimental paradigms may capture distinct aspects of lateralized behavior [36]. In non-human primates, for example, stronger right-handed biases are often reported in tasks involving complex bimanual coordination, including tool use and gestural behaviors [56,57]. These limitations reflect the nature of the available data rather than shortcomings of the present study. Nevertheless, by restricting analyses to a single, widely used behavioral paradigm in non-human primates and applying meta-analytical methods that explicitly model between-study variance [42], we aim to maximize comparability across taxa. Concerns regarding the independence of data points are warranted, as the reuse of subjects across studies cannot be entirely ruled out in the existing literature. Where possible, we minimized this risk through careful screening of study samples at the individual level, which applied to the vast majority of our dataset. In any case, any residual non-independence would be expected to inflate variance rather than systematically bias effect estimates, rendering our conclusions conservative.

Furthermore, the effects of culture are difficult to fully account for in comparative analyses of handedness. Although some non-human primates exhibit forms of cultural transmission [58–60], humans are unique in displaying cumulative cultural evolution, which may amplify or stabilize behavioral asymmetries. This raises the possibility that greater variation in the strength and direction of handedness exists than is captured in the available data [61]. However, the predominance of right-handedness in humans is a robust cross-cultural phenomenon [8,62], and no left-dominant human society has been documented, making it unlikely that culture alone can account for the evolutionary trajectory of handedness in our species.

Taken together, it is plausible that bipedalism and encephalization did not operate as independent drivers of handedness but acted synergistically. The initial adoption of an upright gait freed the upper limbs, creating novel opportunities for tool use, gestural communication, and other fine motor behaviors in which lateralization would have conferred performance advantages. Although terrestrial species generally exhibit lower handedness levels, humans may represent an alternative expression of the arboreal handedness pattern, despite being primarily terrestrial. The freeing of our hands from locomotor constraints allowed them to be deployed asymmetrically more frequently and extensively than in any other anthropoid lineage. This likely had precursors in an ancestor engaged in significant arboreal activities, as evidenced by the hominin fossil record, which indicates that arboreal behaviors were crucial not only at the origin of bipedalism but also in later lineages [63,64]. Concurrently, increases in brain size and associated cortical reorganization may have promoted greater hemispheric specialization, thereby enhancing the neural efficiency of such lateralized behaviors, especially after the emergence of the genus *Homo*. In this context, the initial locomotor change prompted by bipedalism can be seen as providing ecological and anatomical opportunities for manual specialization, while encephalization may have later reinforced and further canalized population-level patterns of lateralization. Furthermore, culture may have acted concurrently with or amplified the effects of this emerging trajectory of hominin right-handedness.

While our analysis provides substantial evidence for the role of both ecological and anatomical factors in the evolution of handedness, it also raises important questions for future research. Notably, our findings underscore the need to refine hypotheses that distinguish human-specific factors, such as cumulative cultural evolution, bipedalism, or ECV, from broader primate trends in terms of handedness. Additionally, expanding this analysis to include non-primate taxa which display clear limb lateralization, such as parrots or kangaroos, would be valuable in investigating the potential for convergent evolution in handedness across species [65–67]. Finally, expanding the fossil sample size, by, e.g., using other handedness proxies such as dental wear analyses, could significantly enhance our understanding of handedness evolution in extinct hominins and contribute to more refined and robust phylogenetic models.

In conclusion, this study highlights the complexity of handedness evolution and demonstrates the importance of considering both ecological and anatomical factors in understanding the selective pressures that have shaped human lateralization. Our findings support a more nuanced interpretation in which human handedness, particularly in the context of goal-directed object manipulation, reflects both continuity with broader primate trends and a uniquely pronounced degree of specialization, with humans representing an extreme at the upper end of the handedness spectrum. Future research will benefit from a more comprehensive approach that considers both the primate and broader animal phylogenies, as well as the distinct evolutionary trajectory of *H. sapiens*.

## Materials and methods

We combined the two most recent and comprehensive datasets available for primate handedness to carry out our subsequent analyses (S1 Data). Soto and colleagues 2022 [36] corresponds to a meta-analysis of hand preferences in coordinated bimanual tasks in non-human primates that compiled multiple sources on non-human handedness, whilst Caspar and colleagues 2022 [15] consists of a phylogenetic comparative analysis that combined both new experimental data, as well as published sources on hand preference. To enable meaningful comparisons, we only considered studies that used the so-called “tube task” as this experimental paradigm corresponds to a simple and widely applicable test to determine primate hand preferences [13,68]. Importantly, while human handedness is often determined via questionnaire, these data use results of humans engaging in the “tube task” [43]. We only included data on MHI and MABSHI, as these two variables captured the main aspects of our phenomena of interest, namely the direction and strength of the manual asymmetries. The MHI is calculated by averaging the handedness scores of all individuals in a group, where handedness scores range from −1 (indicating complete left-handedness) to +1 (indicating complete right-handedness), with 0 representing ambidexterity. The MHI provides insight into the overall direction of hand preference within a population or sample. The MABSHI quantifies the strength of hand preference in a group, regardless of the direction of the preference (i.e., left, or right). It is calculated by taking the absolute value of the handedness scores of all individuals in the group, then averaging these absolute values. This is useful for determining the consistency or strength of hand preference within a population, irrespective of whether the preference is for the left or right hand. Therefore, we extracted all the information available for these two variables (i.e., effect sizes, variance measures and sample sizes) from [36] and [15] being careful of not duplicating sources that were referenced in both studies. The final dataset included information from 2,025 individuals across 41 species of anthropoid primates. It is important to bear in mind that in the present study, we exclusively focus on the evolutionary underpinnings of population-level hand preferences, as opposed to studies or analyses that have focused on the individual-level of variation within species.

We downloaded 100 trees from http://vertlife.org/ comprising the 41 anthropoid species for which we have data to be used in throughout our analyses. These phylogenies correspond to Bayesian-inferred trees built using a “backbone-and-patch” approach based on a 31-gene super-matrix [69]. These phylogenies were used in our subsequent comparative analyses. To assess the hypotheses proposed to explain primate and/or human hand preference (S1 Table) we compiled diverse relevant covariates from the literature. These variables are BM, body mass [kg]; ECV, endocranial volume [cm^3^]; FRUIT, percentage of fruits in diet; DIET, percentage of fruits and animals in diet; IMI, intermembral index; DIM, body mass sexual dimorphism; TOOL, tool use [0 = absence, 1 = presence); SUBS, substrate preference [0 = arboreal, 1=both, 2 = terrestrial]; SOC-SYS, social system [0 = solitary, 1 = pair, 2 = group]; CL, intra-sexual competition levels sensu [1–4,70]; EXT, extractive foraging [0 = absence, 1 = presence]; social learning sensu [71] [0 = absence, 1 = presence] (Fig 1; S1 Table; S1 Data). In the case of IMI and body mass sexual dimorphism (DIM), there were a limited number of species for which we did not find adequate data in the literature (see S1 Data for more details). Therefore, we applied a phylogenetic imputation procedure known as “PhyloPars” to deal with this missing data. Phenotypic covariance was assumed to be equivalent among species, and we also assumed Brownian motion in our imputation procedure. We used the maximum credibility clade phylogeny from our 100 trees when carrying out this procedure. Missing observations were incorporated by maximizing the log-likelihood of the covariance parameters using relevant covariates (i.e., body mass for IMI and body mass sexual dimorphism), thus allowing us to predict means and covariances for missing values at the tips of the phylogenetic tree [72]. This phylogenetic imputation procedure was carried out using the R package “Rphylopars” v.0.3.9 [73]. All the covariates that were considered in our subsequent modeling procedures were used as shown in S1 Table to test the 10 hypotheses proposed to explain handedness. Continuous predictors were logged_10_ and scaled prior to the modeling steps, while the few proportion variables were logit-transformed to ensure numerical stability.

All our Bayesian meta-analytical phylogenetic comparative analyses were done using the “brms” package, which provides an R interface to fit Bayesian generalized linear and non-linear multivariate models using Stan [74]. We applied generic weakly informative priors as all our continuous predictors were standardized (i.e., scaled, and centered). Continuous and categorical predictors were modeled using a normal distribution with mean 0 and variance 1. Prior predictive checks show that using wider priors (e.g., mean 0 and variance 5 or 10) did not influence our obtained results, and therefore narrower priors were preferred for computational efficiency. All predictors shown in S1 Table were treated as fixed effects. Our random effects comprised non-phylogenetic species effects (i.e., specific effects that would be independent of the phylogenetic relationship between species such as environmental or niche effects), as well as phylogenetic species-effects assumed to be sampled from a normal distribution with a mean of 0 and covariance matrix proportional to the phylogenetic correlation matrix among taxa obtained from our phylogenetic trees. Between-study heterogeneity standard deviation (τ) was explicitly modeled via the group-level effects per study. Hence, we used (τ^2^), which corresponds the variance of true effects, as a measure of between-study heterogeneity. All random effects were modeled using half-Cauchy distributions with location 0 and scale 0.05, as our random-effect parameters should always be non-negative but can be close to zero [75]. As our models were also meta-analytical, each dependent variable (i.e., MHI or MABSHI) had an associated measurement error defined by the standard error of every specific study. We considered an effect to be credible (i.e., “significant”) for handedness patterns when the model’s 95% credible intervals for the intercept and corresponding slopes did not include zero. Phylogenetic signal was measured using Lynch’s *h*^2^, which is equivalent to Pagel’s *λ* in the context of phylogenetic generalized linear mixed models [76,77]. We ran two independent chains per model using a warmup period of 4,000 out of a total of 12,000 iterations. Convergence was assessed both visually, by looking at the obtained trace plots, as well as by using rstan’s standard convergence and efficiency diagnostic metrics for Markov chains. The Gelman-Rubin values were always one for all the estimated effects in all our models [78], thus confirming that our chains were well-mixed. Bulk and Tail Effective Sample Sizes were >1,000 for all the effects estimated by all our models, hence indicating good sampling efficiency, as well as that all our estimates were reliable.

We repeated all our analyses separately for both variables, MHI and MABSHI, as different hypotheses may disparately apply to manual strength and/or direction asymmetry. In addition, we also repeated all our analyses excluding *H. sapiens*, to assess how our highly derived handedness patterns may influence the overall pattern observed across anthropoids. To account for topological differences, we repeated all our analyses using the 100 phylogenies previously mentioned. Values reported correspond to the average of the 100 models ran for each one of our analyses. We compared those models showing at least one credible predictor using an efficient approximate leave-one-out cross-validation approach (LOO-CV) [79]. Observations that were too influential in each model and that could not be accurately approximated were estimated using an exact cross-validation. To assess the fitness of our models, we carried out posterior predictive checks by comparing 1,000 datasets simulated from each one of our models with our original data (i.e., MHI and MABSHI) (S1 and S2 Sections). All our models showed simulated datasets that resembled our original data, thus indicating the good fit of our models. We also ran additional models including, as fixed effects, all predictors that showed a credible effect in at least one of the tested hypotheses. We then applied a model reduction procedure, iteratively removing predictors whose 95% credible intervals overlapped zero. This process resulted in four reduced models (two for MABSHI and two for MHI, each including and excluding *H. sapiens*) that retained only covariates with credible effects. The idea was to assess what covariates played a key role in the observed handedness patterns. To evaluate the contribution of these covariates on the variance of MHI and MABSHI, we used an R^2^ metric that is suitable for Bayesian multilevel models [80].

To assess *H. sapiens’* apparent singularity, we used our reduced models to predict the expected levels MHI and MABSHI for our own species using a “phylogenetic outlier test” [41], while also considering a meta-analytical component. We computed a posterior distribution of predictions for the dependent variables (i.e., MHI or MABSHI) for a new observation (i.e., in our case *H. sapiens*) given the posterior distribution of the reduced models, the standard error for our species, the phylogenetic effects and the relevant covariates present in the reduced models. Predictive distributions that deviate strongly from the known value (i.e., outliers) provide evidence that the species has undergone a substantial amount of evolutionary change which cannot be accounted for by its phylogenetic position, branch lengths, and evolutionary change in the independent variable. The implication is that the trait has adaptive value for the species in ways not shared by its close relatives. This test was used to evaluate the idea that human handedness levels correspond to an evolutionary singularity (i.e., a derived character unique to us). This allowed us to assess the relative contribution of the covariates present in the reduced models when explaining *H. sapiens* handedness singularity.

To predict hominin MHI and MABSHI values, we used the reduced human models to generate predictions for hominins, while marginalizing over measurement error and non-phylogenetic species effects. We assumed human-level standard errors for our meta-analytical component when computing our predictions. Data on hominin IMI was obtained from [81], whilst ECVs were obtained from [51]. Hominins were categorized as “group” in social system, whereas for substrate we classified *Ardipithecus* as “both,” whilst all the remaining hominin species were classified as “terrestrial.” We grafted the maximum credibility clade phylogeny from [51] to a consensus phylogeny obtained from the previously used 100 anthropoid trees. We removed the hominin species for which we did not have any data, which left us with the following species: *Ar. ramidus*, *Au. afarensis*, *H. ergaster*, *H. erectus*, *H. floresiensis*, and *H. neanderthalensis*.

## Supporting information

S1 Table Eco-evolutionary hypotheses for handedness. Describes the various eco-evolutionary hypotheses for handedness tested in this manuscript. This includes names, definitions, considerations of, fixed effects, and key references. While other hypotheses related to hominin hand exist, only ones which could be defined with testable variables were included in this study.
(DOCX)

S2 Table LOO-CV model comparison for the MHI hypotheses. Showing results of comparing models used to test eco-evolutionary theories for MHI.
(XLSX)

S3 Table LOO-CV model comparison for the MABSHI hypotheses. Showing results of comparing models used to test eco-evolutionary theories for MABSHI.
(XLSX)

S1 Fig MHI Forest Plot. A forest plot of all studies included for MHI.
(PDF)

S2 Fig MABSHI Forest Plot. A forest plot of all studies included for MABSHI.
(PDF)

S1 Section Modeling results for all tested MHI hypotheses. (PDF)

S2 Section Modeling results for all tested MABSHI hypotheses. (PDF)

S1 Code Script to run the phylogenetic meta-analytical models. (R)

S2 Code R workspace file in rdata format to accompany the code. (R)

S1 Data Dataset analyzed in the present study. (CSV)

S2 Data Numerical data used in Figs 2, 3, and 4. (XLSX)

## Abbreviations

ECV: endocranial volume
IMI: inembral index
MABSHI: mean absolute handedness index
MHI: mean handedness index
