# Supplementary material for: Bipedalism and brain expansion explain human handedness
Source: PLoS Biol. 2026 Apr 27;24(4):e3003771. doi: 10.1371/journal.pbio.3003771 (PMC13138751; doi:10.1371/journal.pbio.3003771)
Supplement: S1 Table — Describes the various eco-evolutionary hypotheses for handedness tested in this manuscript. This includes names, definitions, considerations of, fixed effects, and key references. While other hypotheses related to hominin hand exist, only ones which could be defined with testable variables were included in this study. (DOCX) [file pbio.3003771.s001.docx]

**S1 Table. Eco-evolutionary hypotheses for handedness**

| Name | Acronym used in this study | Description | Considerations | Fixed effects | Key references |
| --- | --- | --- | --- | --- | --- |
| Postural Origins Hypothesis | POH | The original Postural Origins Hypothesis (POH) suggests that the handedness observed in primates is a result of adaptations related to feeding behaviours. Initially, a preference for using the left hand developed for visually guided unimanual reaching tasks, such as manipulating fruit, while the right hand was used for maintaining stability during posture and arboreal locomotion. Later, as primates transitioned towards terrestrial locomotion, the constraints of arboreal living decreased, potentially allowing the right hand to specialize in more intricate tasks, like bimanual manipulation | Based on this hypothesis, arboreal primates are expected to be more left-lateralized, whereas terrestrial primates would be more left lateralized. Similarly, more fruit in the diet is expected to increase left-handedness. | Percentage of fruit in diet + substrate preference | MacNeilage (2007)^1^; MacNeilage, Studdert‐Kennedy, and Lindblom (1987)^2^ |
| Alternative Postural Origins Hypothesis | POH2 | The Postural Origins Hypothesis (POH) has been further elaborated to include the influence of hypothetical insectivorous primate ancestors, who displayed a right-hand bias to support their bodies on vertical substrates, while the left hand specialized in fast grasping movements, known as ballistic reaching. In light of this, the hypothesis has been modified to consider not only fruit consumption but also the combined proportion of animal components (both vertebrate and invertebrate) in diet. | We modified this hypothesis to also consider the combined proportion fruit and animal (i.e., vertebrate and or invertebrate) components in diet, as these diet item require a higher degree of manipulation in general. For species without reported animal consumption, we assumed a value of zero. | Percentage of fruit and animals in diet + substrate preference | Ward (1998)^3^ |
| Bipedalism hypothesis | BH | The bipedalism hypothesis (BH) presents another commonly cited explanation for the link between posture and handedness. It suggests that the postural and biomechanical constraints of bipedal stance are crucial factors in the development of handedness within groups. BH proposes that brain lateralization provides motor skill advantages necessary for adopting bipedal posture, which inherently lacks stability and requires precise control of body movements, particularly in maintaining balance stability after disturbances. | As data on the proportion of bipedal behaviour is scare for most primate species, we used the intermembral index (IMI) in combination with substrate preference and body mass as a general proxy of locomotor repertoire as all these variables relate to locomotion. | Intermembral index + substrate preference + body mass + endocranial volume | Sanford, C., K. Guin, and J. P. Ward (1984)^4^; Westergaard, Kuhn, and Suomi (1998)^5^ |
| Tool-use hypothesis | TUH | The Tool Use Hypothesis (TUH) proposes that the prevalent use of the right hand in humans is a trait developed through the utilisation of tools, a characteristic parsimonically considered to have been present in the common ancestor of humans and panins. This theory is supported by research indicating that right-handed actions are associated with the left cerebral hemisphere's ability to manage the complex temporal sequences involved in tool crafting and usage. | We included body mass as predictor for this hypothesis as relative brain size is the covariate of interest | tool use presence + body mass + endocranial volume | Frost (1980)^6^; McGrew and Marchant (1999)^7^ |
| Tool-use and substrate hypothesis | TU-SH | Similar as the above hypothesis but also considering substrate preference. | As with the TUH, but substrate preference (terrestrial v. arboreal) was included as well. | tool use presence + body mass + endocranial volume + substrate preference | Caspar et al (2022) |
| Tool-use and bipedalism hypothesis | TU-BH | Other studies also suggest specific co-evolutionary connections between laterality and various aspects of behaviour. They propose that ecological factors, such as arboreal and terrestrial habitats, postural positions including quadrupedal, tripedal, and bipedal stances, the complexity of tasks, and the utilization of tools, are environmental elements likely to have influenced the development of manual asymmetry in both human and non-human primates. | Similar as with the BH, body mass, intermembral index and substrate preference were used as locomotor proxy | tool use presence + body mass + endocranial volume + substrate preference + intermembral index | Braccini et al (2010); Bailoo et al (2019); Díaz et al (2021)^8^ |
| Tool-use and social organisation hypothesis | TU-SH-SSH | Prieur et al. (2019) proposed the emergence of a rightward bias in modern humans as a result of manual laterality shaped by ecological (terrestrial) and social (group structure) factors, alongside the increasing complexity of daily tasks, including bimanual coordinated actions such as tool use. | Substrate preference was used to account for the ecological context of terrestriality v. arboreality. | substrate preference + social system + tool use presence | Prieur et al., (2019)^9^ |
| Substrate preference, social organisation, bipedalism and tool-use hypothesis | SP-SS-B-TUH | It has been hypothesised that the rightward bias in modern humans' manual laterality likely emerged from their ecological (i.e., terrestrial) and social (i.e., multilevel system) lifestyle. This bias would have been further reinforced by the gradual adoption of bipedal stance linked with bipedal locomotion, as well as the increasing complexity of daily tasks, including bimanual coordinated actions and tool use. | Similar as with the BH, body mass, intermembral index and substrate preference were used as locomotor proxy | substrate preference + social system + tool use presence + body mass + intermembral index | Prieur et al., (2019)^9^ |
| Modified Fighting hypothesis | FH | The fighting hypothesis posits that left-handedness persists due to its rarity providing a surprise advantage in fighting interactions, while being less frequent because of associated health costs. However, evidence for the health cost of left-handedness is lacking, leaving the greater frequency of right-handers unexplained. This hypothesis proposes that the hand used to hold a weapon could have influenced fight outcomes due to the location of vital organs. A left-handed grip exposes the more vulnerable left hemithorax, whereas a right-handed grip exposes the less vulnerable right hemithorax, potentially giving right-handed ancestors a fighting advantage. | As holding weapons is an almost exclusive human behaviour, we generalised this hypothesis to test for the potential association between higher intra-sexual competition levels and a higher lateralisation. We included body mass sexual dimorphism as an extra covariate as a complementary way of accounting for aggression levels. | body mass sexual dimorphism + intra-sexual competition level | Billiard, Faurie and Raymond (2005); Raymond et al (1997) |
| Task complexity hypothesis | TCH | The task-complexity hypothesis suggests that the strength of laterality will increase with increasing task complexity. Therefore, the more simplistic a task, the more likely an animal is to use either hand (thus showing ambidexterity). However, if the task requires both hands, multiple steps, or complementary actions, the likelihood of displaying a lateral preference (particularly to the right) increases. | Since more complex tasks (such as the multi-step material choice, tool manufacture, and use) often necessitate a learning period, social presence or absence of social leaning was included in this model in addition to tool use itself. Similarly, extractive foraging often requires the use of tools and an understanding that a nutritional resource is present but out of sight, so it was included as well. | extractive foraging + social learning + tool use | Frost (1980)^6^; Fagot and Vauclair (1991)^10^ |

**References**

1. MacNeilage PF. Present Status of the Postural Origins Theory. In: Hopkins WD, editor. Special Topics in Primatology. Elsevier; 2007. pp. 58–91. doi:10.1016/S1936-8526(07)05003-8

2. MacNeilage PF, Studdert-Kennedy MG, Lindblom B. Primate handedness reconsidered. Behavioral and Brain Sciences. 1987;10: 247–263. doi:10.1017/S0140525X00047695

3. Ward JP. Left hand advantage for prey capture in the galago (*Galago Moholi*). International Journal of Comparative Psychology. 1998;11: 173–184.

4. Sanford C, Guin K, Ward JP. Posture and laterality in the bushbaby (*Galago senegalensis*). Brain Behav Evol. 1984;25: 217–224. doi:10.1159/000118867

5. Westergaard GC, Kuhn HE, Suomi SJ. Bipedal posture and hand preference in humans and other primates. J Comp Psychol. 1998;112: 55–64. doi:10.1037/0735-7036.112.1.55

6. Frost GT. Tool behavior and the origins of laterality. Journal of Human Evolution. 1980;9: 447–459. doi:10.1016/0047-2484(80)90002-0

7. McGrew WC, Marchant LF. Laterality of hand use pays off in foraging success for wild chimpanzees. Primates. 1999;40: 509–513. doi:10.1007/BF02557586

8. Díaz S, Murray L, Roberts SGB, Rodway P. Between-task consistency, temporal stability and the role of posture in simple reach and fishing hand preference in chimpanzees (Pan troglodytes). Applied Animal Behaviour Science. 2021;242: 105417. doi:10.1016/j.applanim.2021.105417

9. Prieur J, Barbu S, Blois-Heulin C, Lemasson A. The origins of gestures and language: history, current advances and proposed theories. Biological Reviews. 2019;95. doi:10.1111/brv.12576

10. Fagot J, Vauclair J. Manual laterality in nonhuman primates: A distinction between handedness and manual specialization. Psychological Bulletin. 1991;109: 76–89. doi:10.1037/0033-2909.109.1.76
