# Supplementary material for: Bipedalism and brain expansion explain human handedness
Source: PLoS Biol. 2026 Apr 27;24(4):e3003771. doi: 10.1371/journal.pbio.3003771 (PMC13138751; doi:10.1371/journal.pbio.3003771)
Supplement: S2 Fig — A forest plot of all studies included for MABSHI. (PDF) [file pbio.3003771.s005.pdf]

Reference	Species	Sample size	Mean [95% CI]
Caspar et al. 2022[1]	*Ateles fusciceps*	37	0.76 [0.67, 0.86]
Nelson & Boeving 2015[2]	*Ateles fusciceps*	9	0.94 [0.87, 1.00]
Nelson et al., 2015[3]	*Ateles fusciceps*	10	0.70 [0.51, 0.89]
Caspar et al. 2022[1]	*Ateles geoffroyi*	9	0.73 [0.47, 0.99]
Motes Rodrigo et al. 2018[4]	*Ateles geoffroyi*	14	0.89 [0.81, 0.98]
Caspar et al. 2022[1]	*Ateles hybridus*	18	0.92 [0.84, 1.00]
Caspar et al. 2022[1]	*Cercocebus torquatus*	18	0.79 [0.68, 0.90]
Maille et al. 2013[6]	*Cercocebus torquatus*	13	0.49 [0.28, 0.70]
Caspar et al. 2022[1]	*Cercopithecus diana*	20	0.75 [0.62, 0.89]
Caspar et al. 2022[1]	*Cercopithecus neglectus*	12	0.52 [0.32, 0.73]
Maille et al. 2013[5]	*Cercopithecus neglectus*	12	0.72 [0.58, 0.86]
Schweitzer et al., 2007[6]	*Cercopithecus neglectus*	12	0.70 [0.56, 0.84]
Hopkins et al., 2003[7]	*Gorilla gorilla*	31	0.50 [0.38, 0.62]
Hopkins et al., 2011[8]	*Gorilla gorilla*	76	0.54 [0.47, 0.61]
Cochet & Vauclair, 2012[9]	*Homo sapiens*	127	0.94 [0.91, 0.97]
Caspar et al. 2022[1]	*Hylobates lar*	16	0.64 [0.46, 0.81]
Caspar et al., 2018[10]	*Hylobates lar*	3	0.54 [0.08, 0.99]
Morino et al. 2017[11]	*Hylobates lar*	6	0.59 [0.46, 0.72]
Spoelstra 2021[12]	*Hylobates lar*	11	0.62 [0.48, 0.76]
Caspar et al. 2022[1]	*Hylobates moloch*	22	0.80 [0.67, 0.93]
Caspar et al. 2022[1]	*Leontopithecus chrysomelas*	30	0.51 [0.40, 0.63]
Caspar et al. 2022[1]	*Leontopithecus chrysopygus*	15	0.35 [0.21, 0.49]
Caspar et al. 2022[1]	*Leontopithecus rosalia*	28	0.50 [0.38, 0.62]
Caspar et al. 2022[1]	*Macaca fascicularis*	12	0.64 [0.48, 0.81]
Chatagny et al., 2013[13]	*Macaca fascicularis*	8	0.75 [0.54, 0.96]
Zhao et al., 2016[14]	*Macaca leonina*	9	0.49 [0.31, 0.67]
Nelson et al., 2011[15]	*Macaca mulatta*	16	0.45 [0.29, 0.61]
Caspar et al. 2022[1]	*Macaca nemestrina*	29	0.53 [0.40, 0.65]
Caspar et al. 2022[1]	*Macaca silenus*	35	0.47 [0.37, 0.57]
Caspar et al. 2022[1]	*Macaca sylvanus*	15	0.63 [0.48, 0.77]
Regaiolli et al., 2018[16]	*Macaca sylvanus*	6	0.76 [0.47, 1.04]
Schmitt et al., 2008[17]	*Macaca sylvanus*	3	0.72 [0.45, 0.99]
Canteloup et al., 2013[18]	*Macaca tonkeana*	14	0.54 [0.35, 0.73]
Caspar et al. 2022[1]	*Mandrillus sphinx*	32	0.39 [0.28, 0.49]
Caspar et al. 2022[1]	*Nomascus gabriellae*	6	0.47 [0.16, 0.77]
Caspar et al., 2018[10]	*Nomascus gabriellae*	4	0.85 [0.71, 0.99]
Caspar et al. 2022[1]	*Nomascus leucogenys*	7	0.62 [0.36, 0.89]
Caspar et al., 2018[10]	*Nomascus leucogenys*	9	0.62 [0.36, 0.87]
Fan et al., 2017[19]	*Nomascus leucogenys*	9	0.39 [0.16, 0.63]
Caspar et al., 2018[10]	*Nomascus siki*	4	0.73 [0.27, 1.19]
Chapelain & Hogervorst, 2009[20]	*Pan paniscus*	29	0.57 [0.46, 0.68]
Hopkins et al., 2011[8]	*Pan paniscus*	118	0.53 [0.48, 0.58]
Hopkins et al., 2011[8]	*Pan troglodytes*	536	0.51 [0.48, 0.53]
Llorente et al., 2009[21]	*Pan troglodytes*	14	0.80 [0.68, 0.92]
Padrell et al., 2019[22]	*Pan troglodytes*	14	0.81 [0.69, 0.93]
Phillips & Hopkins, 2007[23]	*Pan troglodytes*	16	0.35 [0.21, 0.49]
Vauclair et al. 2005[24]	*Papio anubis*	84	0.53 [0.46, 0.59]
Caspar et al. 2022[1]	*Papio hamadryas*	24	0.41 [0.29, 0.53]
Caspar et al. 2022[1]	*Pithecia pithecia*	7	0.93 [0.86, 1.00]
Hopkins et al., 2003[7]	*Pongo* sp.	19	0.63 [0.36, 0.90]
Hopkins et al., 2011[8]	*Pongo* sp.	47	0.49 [0.40, 0.57]
Cubi & Llorente, 2021[25]	*Pygathrix cinerea*	18	0.50 [0.33, 0.67]
Zhao et al., 2012[26]	*Rhinopithecus roxellana*	24	0.73 [0.63, 0.83]
Meguerditchian et al., 2012[27]	*Saimiri scuireus*	36	0.76 [0.67, 0.85]
de Andrade & de Sousa, 2018[28]	*Sapajus apella*	12	0.61 [0.41, 0.81]
Lilak & Phillips, 2008[29]	*Sapajus apella*	11	0.67 [0.53, 0.81]
Phillips & Hopkins, 2007[23]	*Sapajus apella*	11	0.75 [0.59, 0.91]
Phillips & Sherwood, 2005[30]	*Sapajus apella*	7	0.70 [0.46, 0.94]
Phillips & Sherwood, 2007[31]	*Sapajus apella*	13	0.76 [0.62, 0.90]
Phillips, Sherwood & Lilak, 2007[32]	*Sapajus apella*	13	0.76 [0.62, 0.90]
Spinozzi et al., 1998[33]	*Sapajus apella*	26	0.82 [0.75, 0.89]
Caspar et al. 2022[1]	*Sapajus flavius*	3	0.68 [0.06, 1.30]
de Andrade & de Sousa, 2018[29]	*Sapajus flavius*	18	0.78 [0.62, 0.94]
Caspar et al. 2022[1]	*Sapajus xanthosternos*	16	0.64 [0.49, 0.79]
de Andrade & de Sousa, 2018[28]	*Sapajus xanthosternos*	18	0.71 [0.57, 0.85]
Caspar et al. 2022[1]	*Semnopithecus entellus*	30	0.56 [0.46, 0.66]
Caspar et al. 2022[1]	*Symphalangus syndactylus*	14	0.57 [0.38, 0.76]
Morino et al. 2017[11]	*Symphalangus syndactylus*	16	0.38 [0.21, 0.55]
Caspar et al. 2022[1]	*Theropithecus gelada*	38	0.26 [0.19, 0.32]
Caspar et al. 2022[1]	*Trachypithecus auratus*	8	0.98 [0.96, 1.01]
Cubi & Llorente, 2021[25]	*Trachypithecus hatinhensis*	18	0.82 [0.73, 0.91]

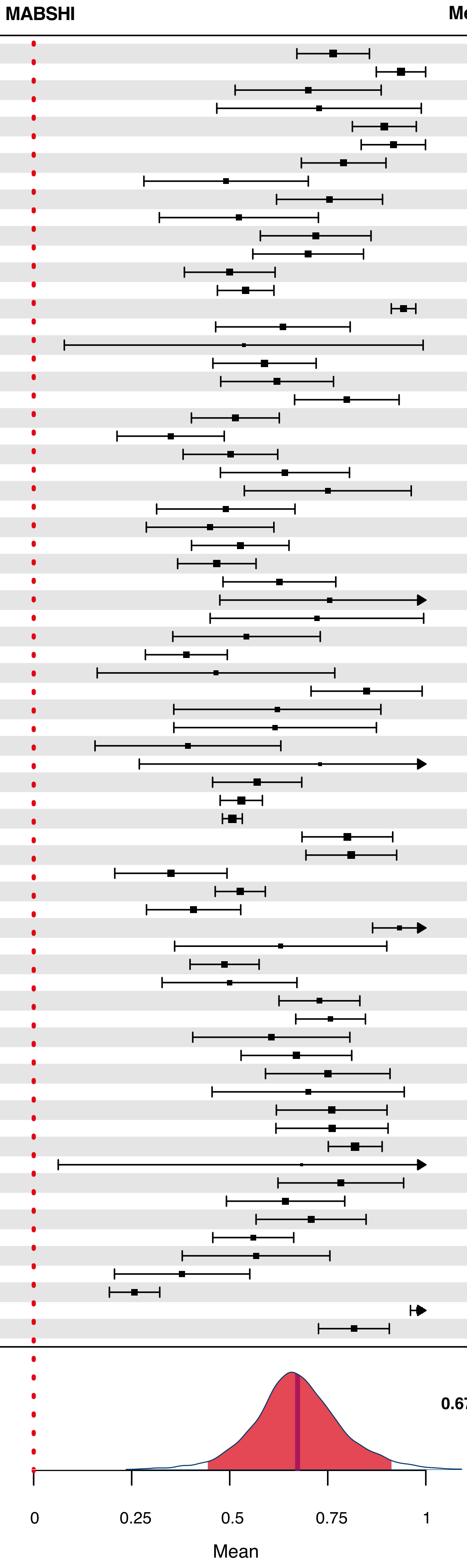

0.67 [0.44, 0.91]

S2 Figure References

1. Caspar KR, Pallasdies F, Mader L, Sartorelli H, Begall S. The evolution and biological correlates of hand preferences in anthropoid primates. Tung J, Przeworski M, DeCasien A, Meguerditchian A, editors. eLife. 2022;11: e77875. doi:10.7554/eLife.77875

2. Nelson EL, Boeving ER. Precise digit use increases the expression of handedness in Colombian spider monkeys (*Ateles fusciceps rufiventris*). Am J Primatol. 2015;77: 1253–1262. doi:10.1002/ajp.22478

3. Nelson EL, Figueroa A, Albright SN, Gonzalez MF. Evaluating handedness measures in spider monkeys. Anim Cogn. 2015;18: 345–353. doi:10.1007/s10071-014-0805-5

4. Motes Rodrigo A, Ramirez Torres CE, Hernandez Salazar LT, Laska M. Hand preferences in two unimanual and two bimanual coordinated tasks in the black-handed spider monkey (*Ateles geoffroyi*). J Comp Psychol. 2018;132: 220–229. doi:10.1037/com0000110

5. Maille A, Belbeoc'h C, Rossard A, Bec P, Blois-Heulin C. Which are the features of the TUBE task that make it so efficient in detecting manual asymmetries? An investigation in two Cercopithecine species (*Cercopithecus neglectus* and *Cercocebus torquatus*). Journal of Comparative Psychology. 2013;127: 436–444. doi:10.1037/a0032227

6. Schweitzer C, Bec P, Blois-Heulin C. Does the Complexity of the Task Influence Manual Laterality in De Brazza's Monkeys (*Cercopithecus neglectus*)? Ethology. 2007;113: 983–994. doi:10.1111/j.1439-0310.2007.01405.x

7. Hopkins WD, Stoinski TS, Lukas KE, Ross SR, Wesley MJ. Comparative assessment of handedness for a coordinated bimanual task in chimpanzees (*Pan troglodytes*), gorillas (*Gorilla gorilla*) and orangutans (*Pongo pygmaeus*). J Comp Psychol. 2003;117: 302–308. doi:10.1037/0735-7036.117.3.302

8. Hopkins WD, Phillips KA, Bania A, Calcutt SE, Gardner M, Russell J, et al. Hand preferences for coordinated bimanual actions in 777 great apes: Implications for the evolution of handedness in Hominins. Journal of Human Evolution. 2011;60: 605–611. doi:10.1016/j.jhevol.2010.12.008

9. Cochet H, Vauclair J. Hand preferences in human adults: Non-communicative actions versus communicative gestures. Cortex. 2012;48: 1017–1026. doi:10.1016/j.cortex.2011.03.016

10. Caspar K, Mader L, Pallasdies F, Lindenmeier M, Begall S. Captive gibbons (*Hylobatidae*) use different referential cues in an object-choice task: insights into lesser ape cognition and manual laterality. PeerJ. 2018;6. doi:10.7717/peerj.5348

11. Morino L, Uchikoshi M, Bercovitch F, Hopkins WD, Matsuzawa T. Tube task hand preference in captive hylobatids. Primates. 2017;58: 403–412. doi:10.1007/s10329-017-0605-z

12. Spoelstra K. Lateralized behavior in white- handed gibbons (Hylobates lar).

13. Chatagny P, Badoud S, Kaeser M, Gindrat A-D, Savidan J, Fregosi M, et al. Distinction between hand dominance and hand preference in primates: a behavioral investigation of manual dexterity in nonhuman primates (macaques) and human subjects. Brain and Behavior. 2013;3: 575–595. doi:10.1002/brb3.160

14. Zhao D, Wang Y, Wei X. Hand preference during bimanual coordinated task in northern pig-tailed macaques *Macaca leonina*. Curr Zool. 2016;62: 385–391. doi:10.1093/cz/zow064

15. Nelson EL, Emery MS, Babcock SM, Novak MFSX, Suomi SJ, Novak MA. Head orientation and handedness trajectory in rhesus monkey infants (*Macaca mulatta*). Developmental psychobiology. 2011;53. doi:10.1002/dev.20517

16. Regaiolli B, Spiezio C, Hopkins WD. Asymmetries in mother-infant behaviour in Barbary macaques (*Macaca sylvanus*). PeerJ. 2018;6: e4736. doi:10.7717/peerj.4736

17. Schmitt V, Melchisedech S, Hammerschmidt K, Fischer J. Hand preferences in Barbary macaques (*Macaca sylvanus*). Laterality. 2008;13: 143–157. doi:10.1080/13576500701757532

18. Canteloup C, Vauclair J, Meunier H. Hand preferences on unimanual and bimanual tasks in Tonkean macaques (*Macaca tonkeana*). American J Phys Anthropol. 2013;152: 315–321. doi:10.1002/ajpa.22342

19. Fan P, Liu C, Chen H, Liu X, Zhao D, Zhang J, et al. Preliminary study on hand preference in captive northern white-cheeked gibbons (*Nomascus leucogenys*). Primates. 2017;58: 75–82. doi:10.1007/s10329-016-0573-8

20. Chapelain A, Hogervorst E. Hand preferences for bimanual coordination in 29 bonobos (*Pan paniscus*). Behavioural brain research. 2008;196: 15–29. doi:10.1016/j.bbr.2008.07.012

21. Llorente M, Mosquera M, Fabré M. Manual Laterality for Simple Reaching and Bimanual Coordinated Task in Naturalistic Housed Pan troglodytes. International Journal of Primatology. 2009;30: 183–197. doi:10.1007/s10764-009-9338-1

22. Padrell M, Gómez-Martínez C, Llorente M. Short and long-term temporal consistency of hand preference in sanctuary chimpanzees (*Pan troglodytes*) for unimanual and bimanual coordinated tasks. Behavioural Processes. 2019;167: 103911. doi:10.1016/j.beproc.2019.103911

23. Phillips KA, Hopkins WD. Exploring the relationship between cerebellar asymmetry and handedness in chimpanzees (*Pan troglodytes*) and capuchins (*Cebus apella*). Neuropsychologia. 2007;45: 2333–2339. doi:10.1016/j.neuropsychologia.2007.02.010

24. Vauclair J, Meguerditchian A, Hopkins WD. Hand preferences for unimanual and coordinated bimanual tasks in baboons (*Papio anubis*). Cognitive Brain Research. 2005;25: 210–216. doi:10.1016/j.cogbrainres.2005.05.012

25. Cubí M, Llorente M. Hand preference for a bimanual coordinated task in captive hatinh langurs (*Trachypithecus hatinhensis*) and grey-shanked douc langurs (*Pygathrix cinerea*). Behav Processes. 2021;187: 104393. doi:10.1016/j.beproc.2021.104393

26. Zhao D, Hopkins WD, Li B. Handedness in nature: first evidence on manual laterality on bimanual coordinated tube task in wild primates. Am J Phys Anthropol. 2012;148: 36–44. doi:10.1002/ajpa.22038

27. Meguerditchian A, Donnot J, Molesti S, Francioly R, Vauclair J. Sex difference in squirrel monkeys' handedness for unimanual and bimanual coordinated tasks. Animal Behaviour. 2012;83: 635–643. doi:10.1016/j.anbehav.2011.12.005

28. de Andrade AC, de Sousa AB. Hand preferences and differences in extractive foraging in seven capuchin monkey species. American Journal of Primatology. 2018;80: e22901. doi:10.1002/ajp.22901

29. Lilak A, Phillips K. Consistency of Hand Preference across Low-Level and High-Level Tasks in Capuchin Monkeys (*Cebus apella*). American journal of primatology. 2008;70: 254–60. doi:10.1002/ajp.20485

30. Phillips KA, Sherwood CC. Primary motor cortex asymmetry is correlated with handedness in capuchin monkeys (*Cebus apella*). Behav Neurosci. 2005;119: 1701–1704. doi:10.1037/0735-7044.119.6.1701

31. Phillips KA, Sherwood CC. Cerebral petalias and their relationship to handedness in capuchin monkeys (*Cebus apella*). Neuropsychologia. 2007;45: 2398–2401. doi:10.1016/j.neuropsychologia.2007.02.021

32. Phillips KA, Sherwood CC, Lilak AL. Corpus Callosum Morphology in Capuchin Monkeys Is Influenced by Sex and Handedness. PLOS ONE. 2007;2: e792. doi:10.1371/journal.pone.0000792

33. Spinozzi G, Castorina MG, Truppa V. Hand preferences in unimanual and coordinated-bimanual tasks by tufted capuchin monkeys (*Cebus apella*). Journal of Comparative Psychology. 1998;112: 183–191. doi:10.1037/0735-7036.112.2.183
