# Supplementary material for: Bipedalism and brain expansion explain human handedness
Source: PLoS Biol. 2026 Apr 27;24(4):e3003771. doi: 10.1371/journal.pbio.3003771 (PMC13138751; doi:10.1371/journal.pbio.3003771)

S2 Section: MABSHI

Thomas A. Püschel, Rachel M. Hurwitz, Chris Venditti

MABSHI results excluding *Homo sapiens*

Numerical results for all the hypotheses tested, using MABSHI as the dependent variable, are presented here (see Supplementary Table 1 for further details). *Homo sapiens* was excluded from these analyses. In addition, the final reduced model is also presented after all the hypotheses tested. Posterior predictive checks for each of the tested hypotheses are shown, along with plots illustrating the estimates for the fixed effects included in each of the tested models.

0) Intercepts-only model

Summary

effect	component	term	estimate	std.error	conf.low	conf.high	rhat
fixed	cond	(Intercept)	0.67	0.11	0.46	0.88	1

effect	component	group	term	estimate	std.error	conf.low	conf.high	rhat
ran_pars	cond	obs	sd___(Intercept)	0.10	0.02	0.07	0.14	1
ran_pars	cond	phylo	sd___(Intercept)	0.03	0.01	0.01	0.05	1
ran_pars	cond	species	sd___(Intercept)	0.04	0.03	0.00	0.10	1

Posterior predictive check; 100 simulated datasets

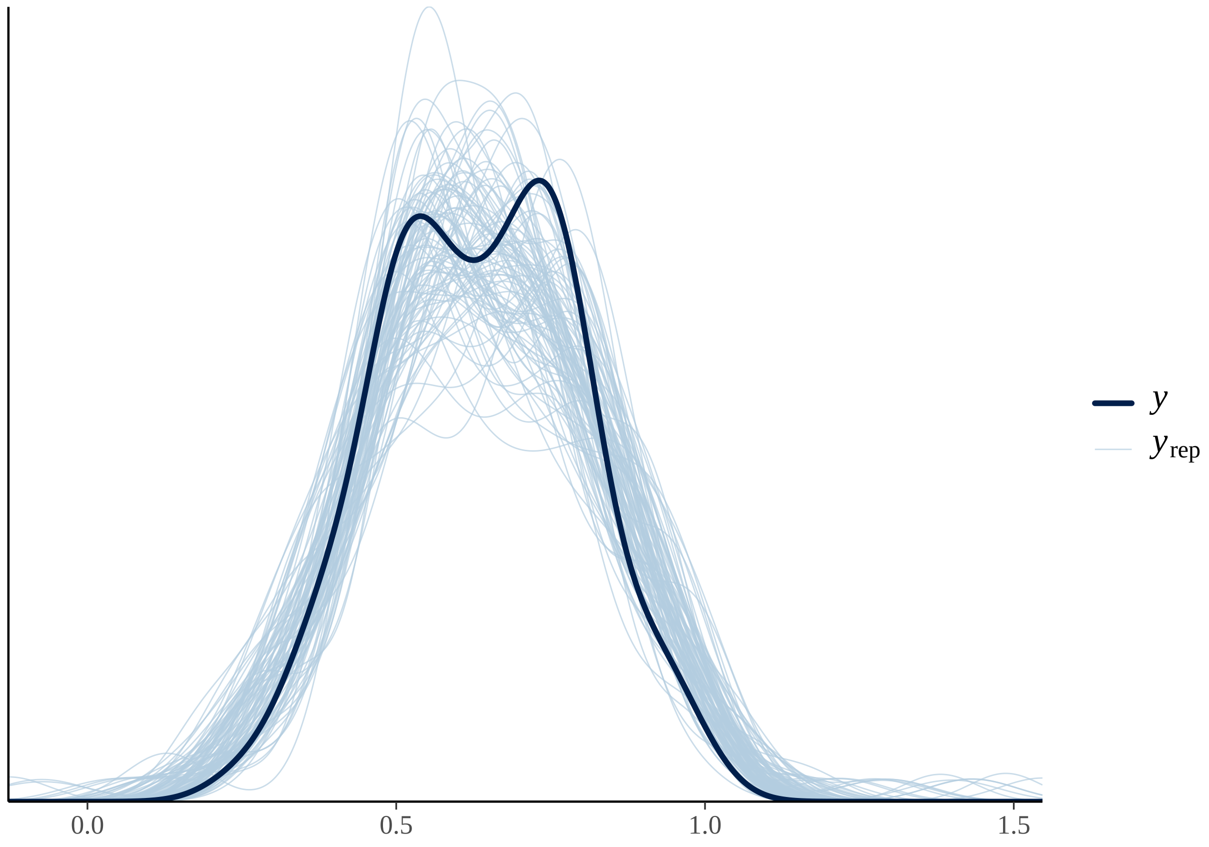

Fixed effects results

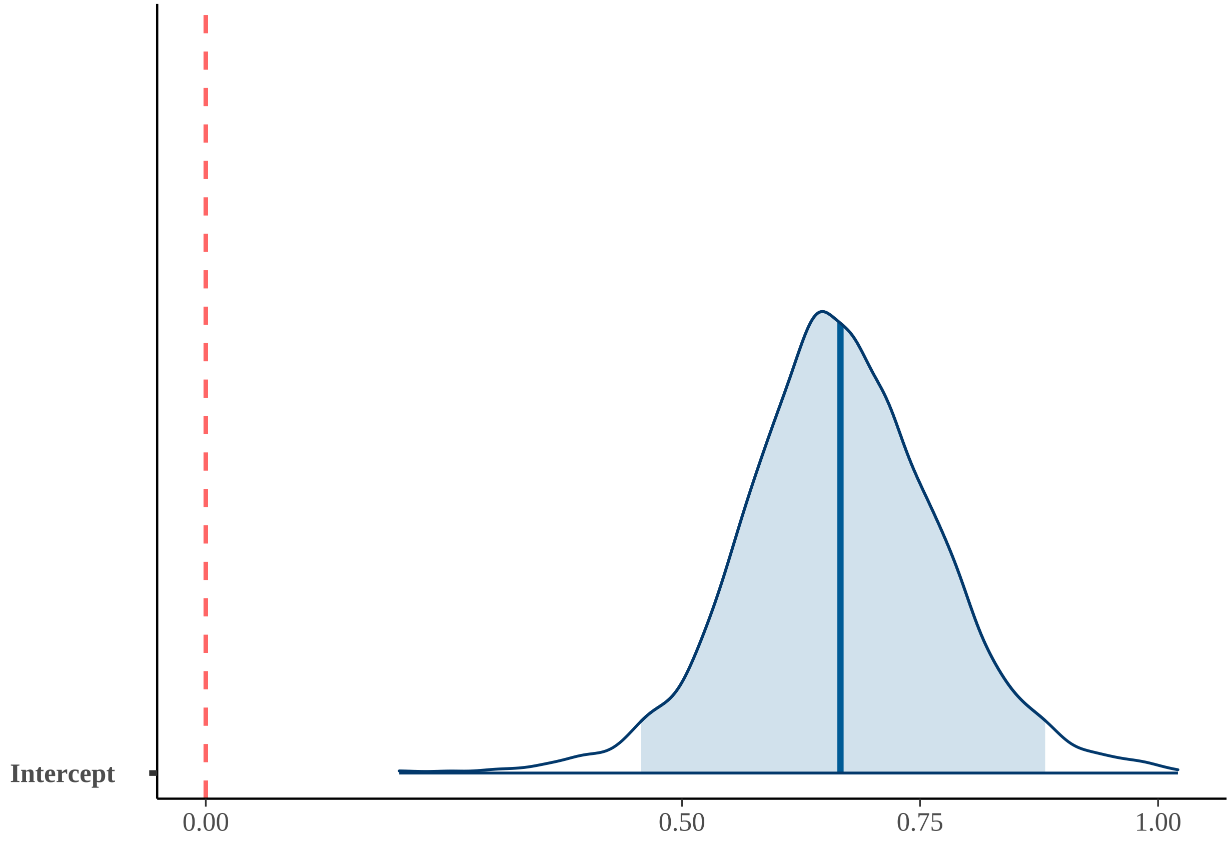

1) POH1

Summary POH1

effect	component	term	estimate	std.error	conf.low	conf.high	rhat
fixed	cond	(Intercept)	0.68	0.10	0.48	0.88	1
fixed	cond	fruit	0.02	0.02	-0.02	0.06	1
fixed	cond	substrateBOTH	-0.02	0.06	-0.14	0.11	1
fixed	cond	substrateT	-0.23	0.10	-0.42	-0.04	1

effect	component	group	term	estimate	std.error	conf.low	conf.high	rhat
ran_pars	cond	obs	sd__(Intercept)	0.09	0.02	0.06	0.13	1
ran_pars	cond	phylo	sd__(Intercept)	0.03	0.01	0.01	0.05	1
ran_pars	cond	species	sd__(Intercept)	0.03	0.02	0.00	0.09	1

Posterior predictive check POH1; 100 simulated datasets

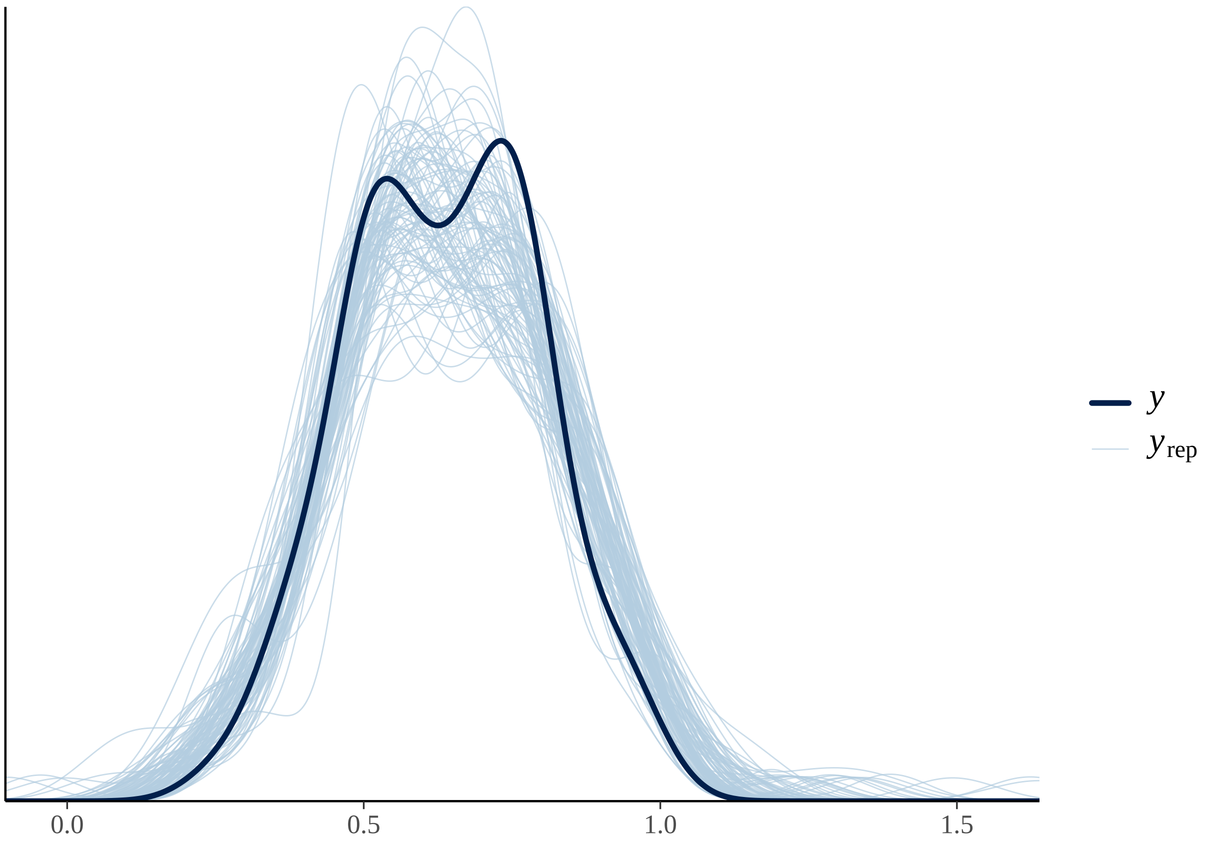

Fixed effects results POH1

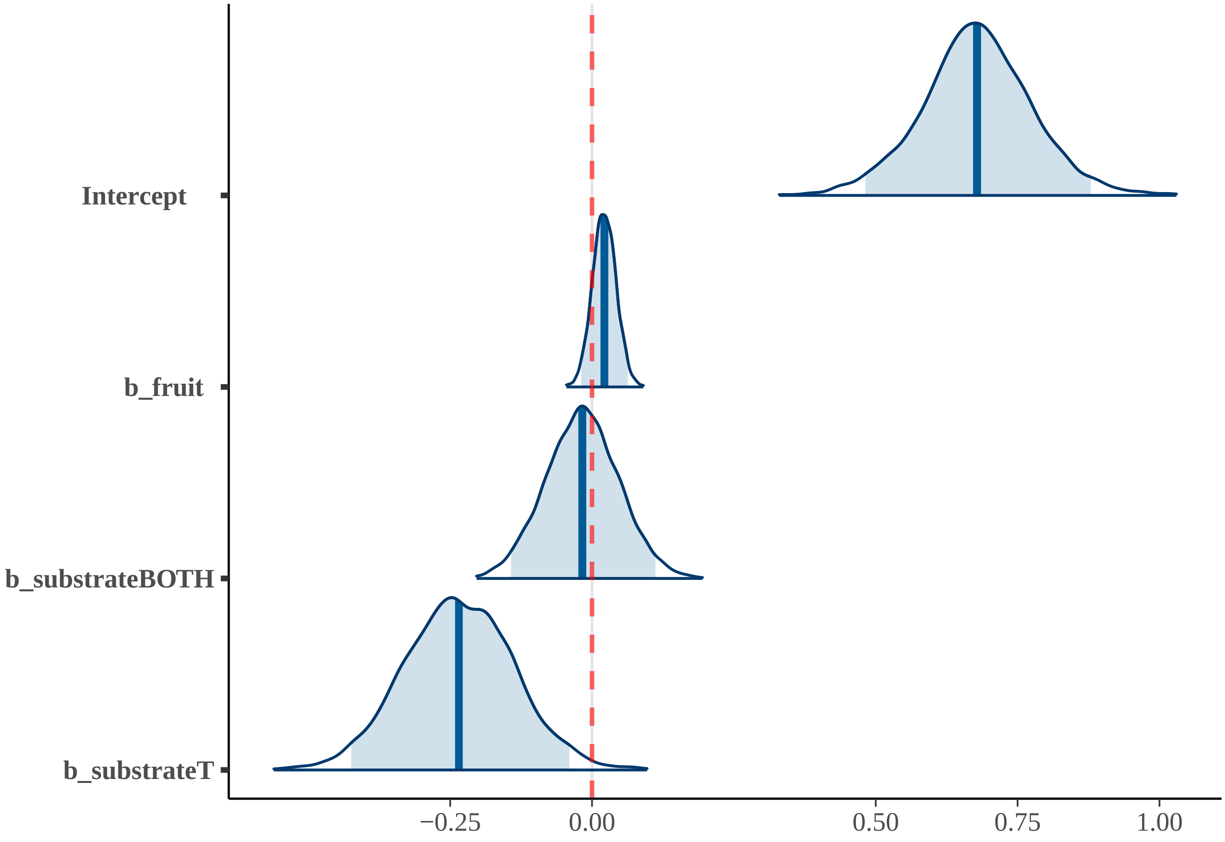

2) POH2

Summary POH2

effect	component	term	estimate	std.error	conf.low	conf.high	rhat
fixed	cond	(Intercept)	0.67	0.10	0.46	0.88	1
fixed	cond	diet	0.01	0.02	-0.03	0.06	1
fixed	cond	substrateBOTH	-0.02	0.07	-0.15	0.11	1
fixed	cond	substrateT	-0.24	0.10	-0.44	-0.05	1

effect	component	group	term	estimate	std.error	conf.low	conf.high	rhat
ran_pars	cond	obs	sd__(Intercept)	0.09	0.02	0.06	0.13	1
ran_pars	cond	phylo	sd__(Intercept)	0.03	0.01	0.01	0.05	1
ran_pars	cond	species	sd__(Intercept)	0.03	0.02	0.00	0.09	1

Posterior predictive check POH2; 100 simulated datasets

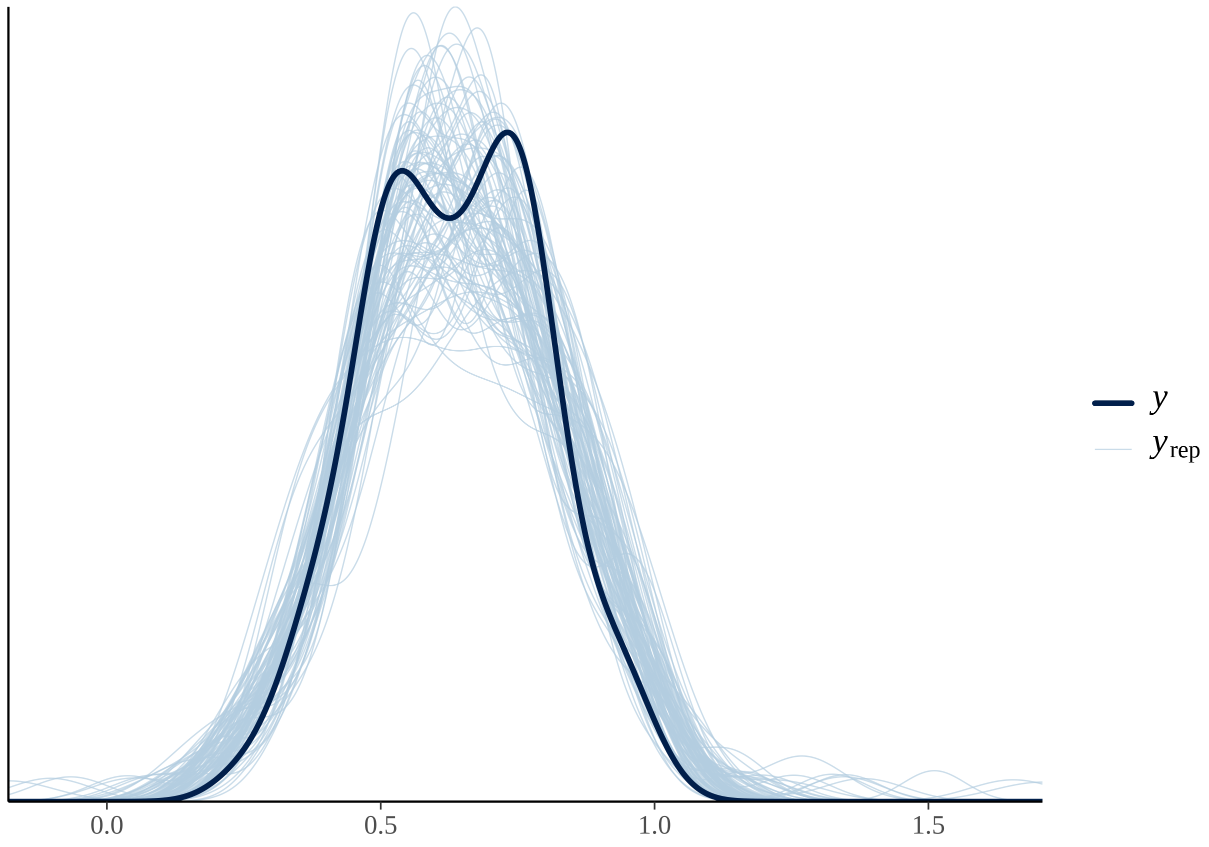

Fixed effects results POH2

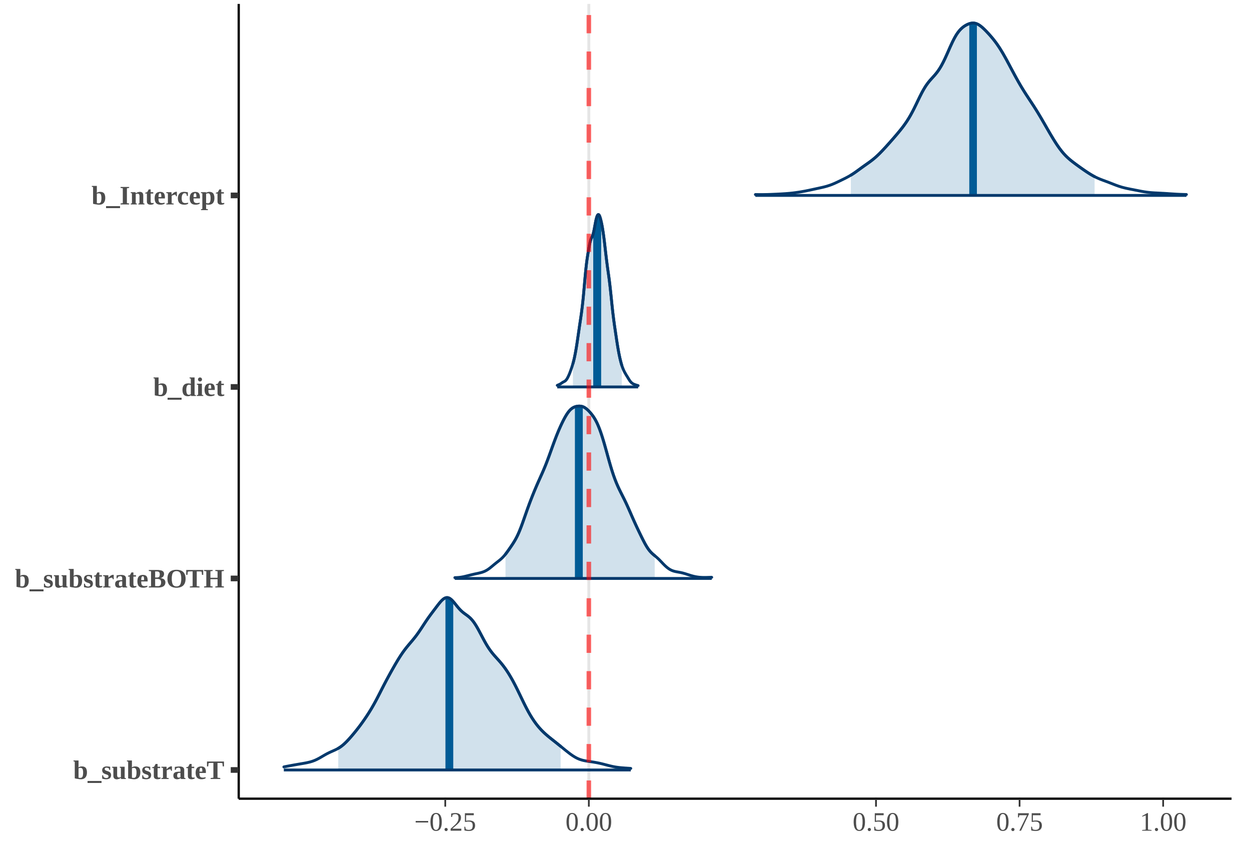

3) BH

Summary BH

effect	component	term	estimate	std.error	conf.low	conf.high	rhat
fixed	cond	(Intercept)	0.70	0.09	0.51	0.89	1
fixed	cond	imi	-0.08	0.05	-0.18	0.03	1
fixed	cond	ecv	0.08	0.10	-0.11	0.27	1
fixed	cond	bm	-0.05	0.10	-0.24	0.14	1
fixed	cond	substrateBOTH	-0.08	0.08	-0.22	0.08	1
fixed	cond	substrateT	-0.30	0.11	-0.51	-0.09	1

effect	component	group	term	estimate	std.error	conf.low	conf.high	rhat
ran_pars	cond	obs	sd__(Intercept)	0.10	0.02	0.06	0.13	1
ran_pars	cond	phylo	sd__(Intercept)	0.03	0.01	0.01	0.05	1
ran_pars	cond	species	sd__(Intercept)	0.03	0.03	0.00	0.10	1

Posterior predictive check BH; 100 simulated datasets

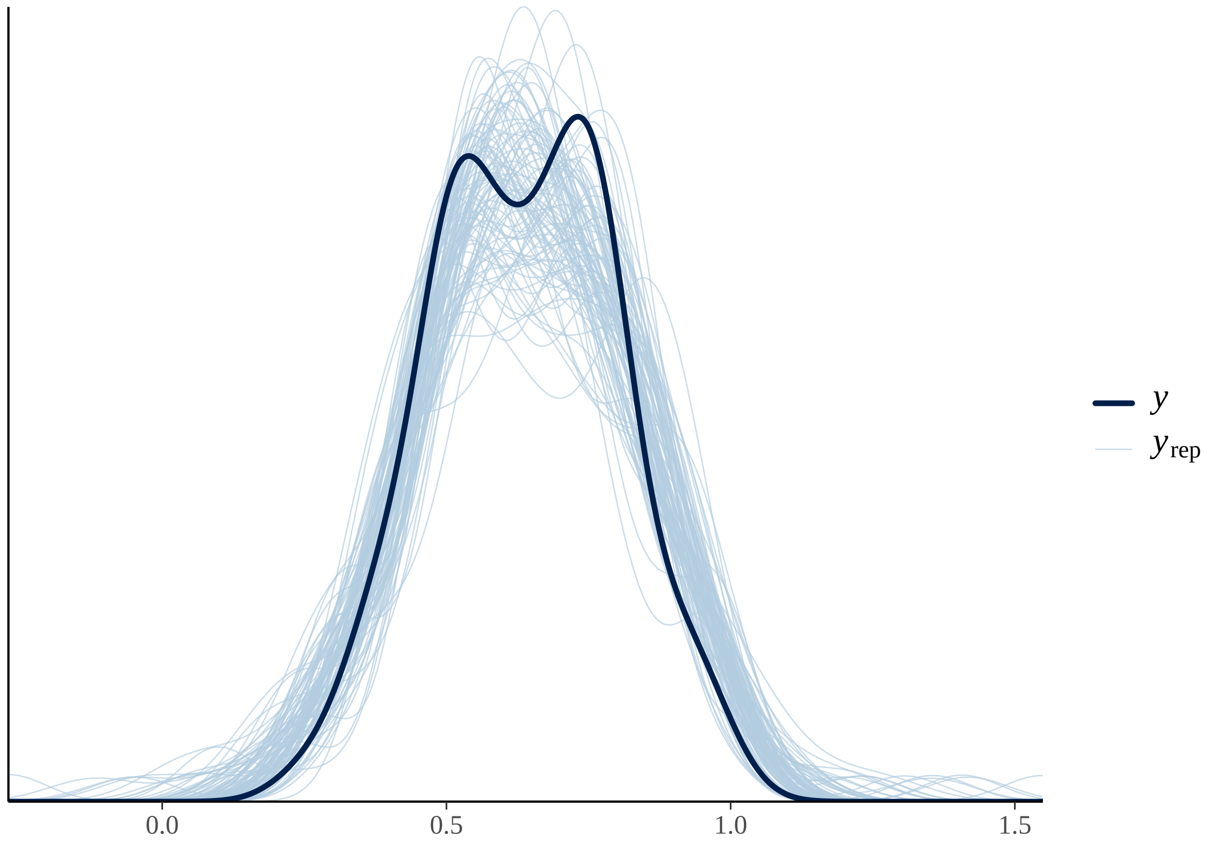

Fixed effects results BH

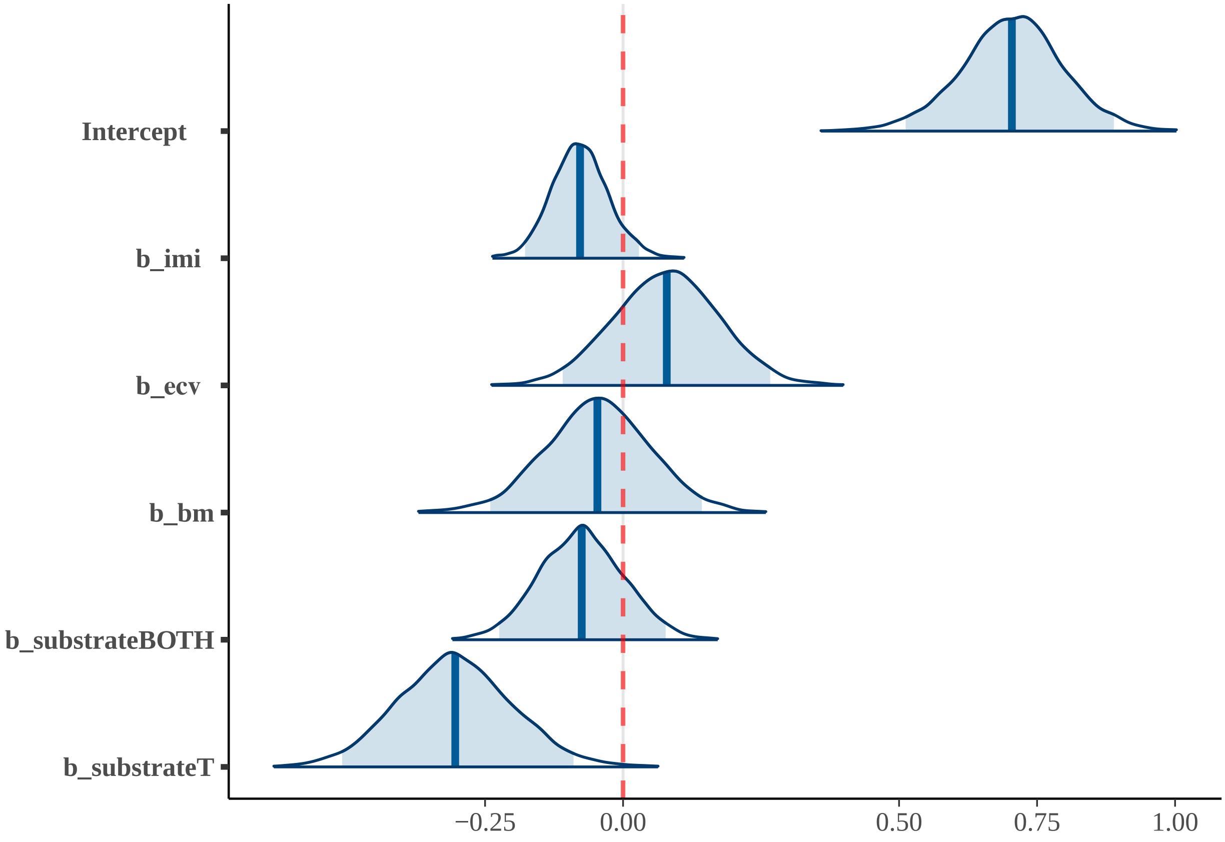

4) TUH

Summary TUH

effect	component	term	estimate	std.error	conf.low	conf.high	rhat
fixed	cond	(Intercept)	0.65	0.12	0.41	0.90	1
fixed	cond	tool_useYes	0.06	0.08	-0.09	0.21	1
fixed	cond	ecv	0.05	0.12	-0.18	0.28	1
fixed	cond	bm	-0.09	0.12	-0.32	0.14	1

effect	component	group	term	estimate	std.error	conf.low	conf.high	rhat
ran_pars	cond	obs	sd__(Intercept)	0.10	0.02	0.06	0.14	1
ran_pars	cond	phylo	sd__(Intercept)	0.04	0.01	0.02	0.06	1
ran_pars	cond	species	sd__(Intercept)	0.04	0.03	0.00	0.10	1

Posterior predictive check TUH; 100 simulated datasets

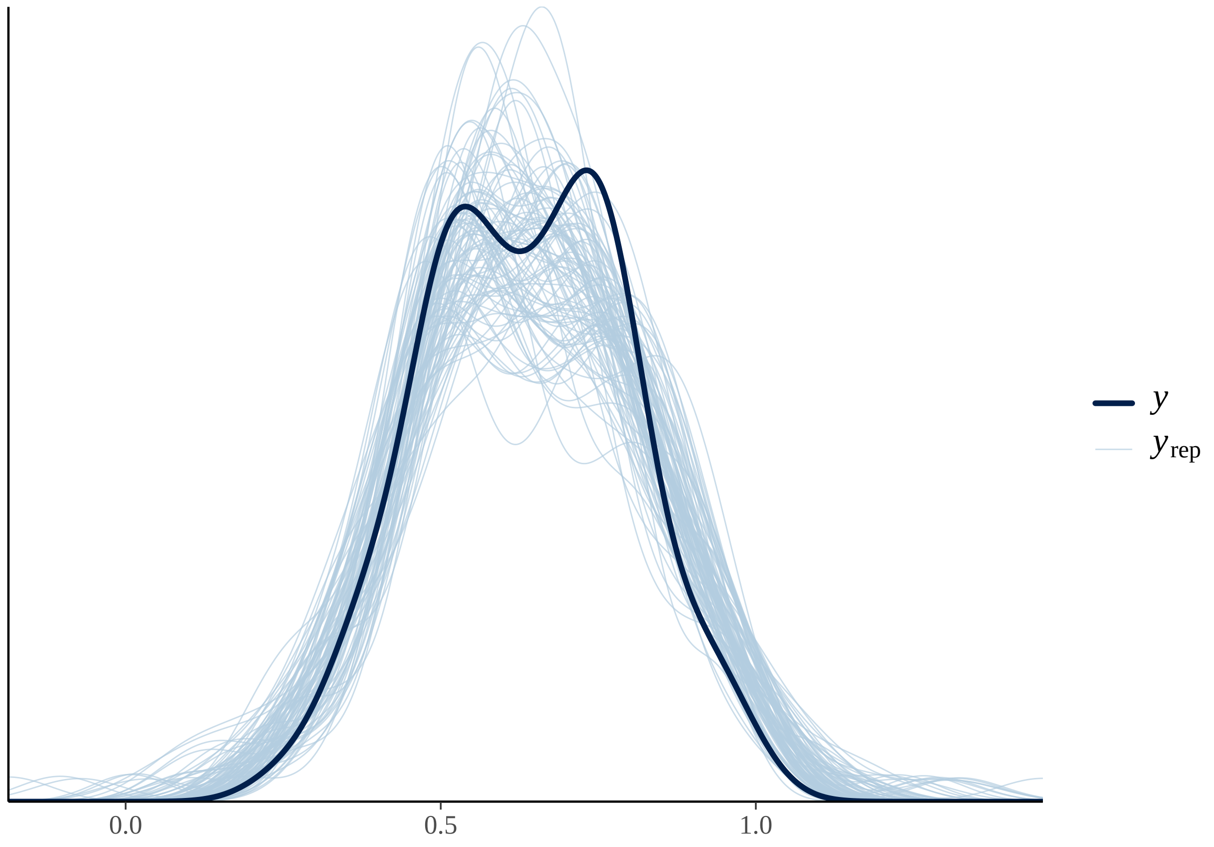

Fixed effects results TUH

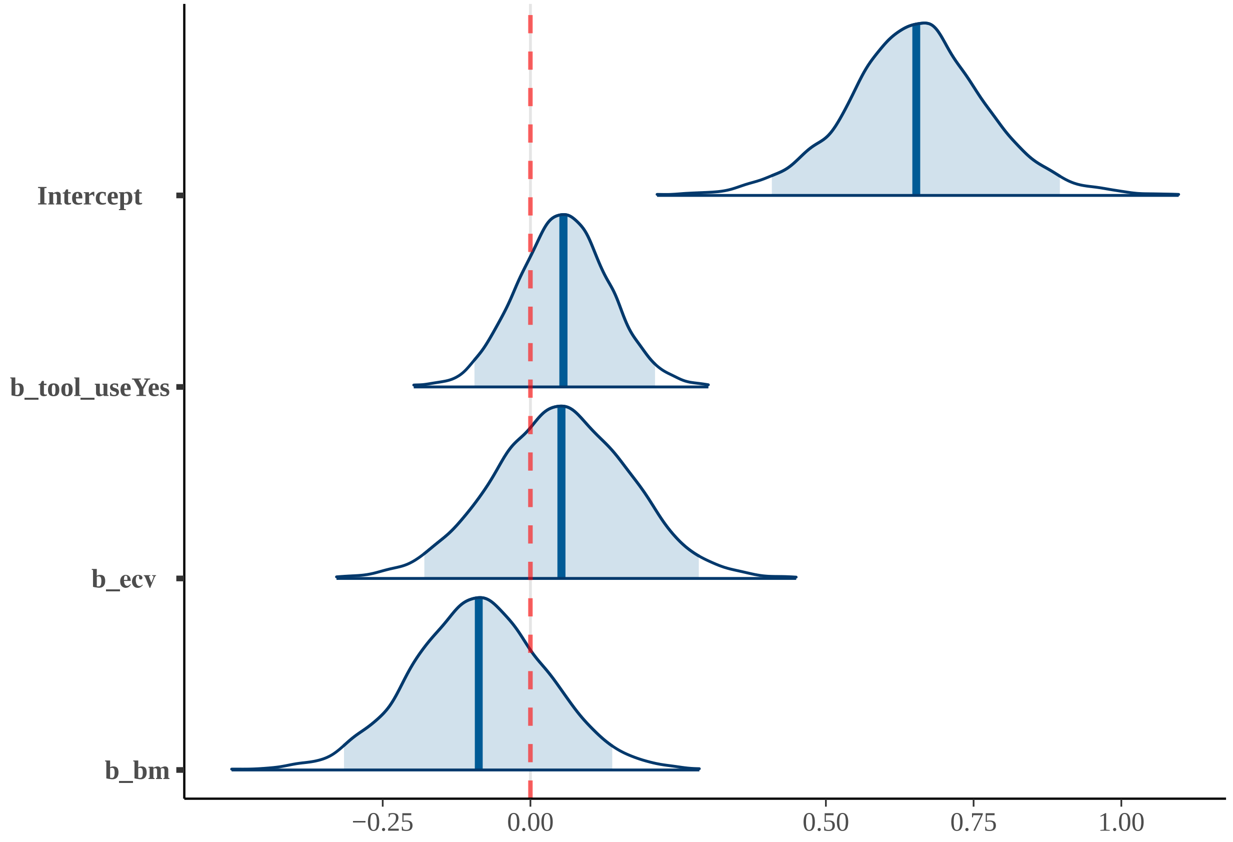

5) TU-SH

Summary TU-SH

effect	component	term	estimate	std.error	conf.low	conf.high	rhat
fixed	cond	(Intercept)	0.68	0.11	0.46	0.90	1
fixed	cond	tool_useYes	0.04	0.07	-0.10	0.19	1
fixed	cond	ecv	0.04	0.11	-0.17	0.25	1
fixed	cond	bm	-0.05	0.11	-0.26	0.16	1
fixed	cond	substrateBOTH	-0.02	0.07	-0.16	0.12	1
fixed	cond	substrateT	-0.24	0.10	-0.45	-0.04	1

effect	component	group	term	estimate	std.error	conf.low	conf.high	rhat
ran_pars	cond	obs	sd__(Intercept)	0.09	0.02	0.06	0.13	1
ran_pars	cond	phylo	sd__(Intercept)	0.03	0.01	0.02	0.05	1
ran_pars	cond	species	sd__(Intercept)	0.03	0.02	0.00	0.09	1

Posterior predictive check TU-SH; 100 simulated datasets

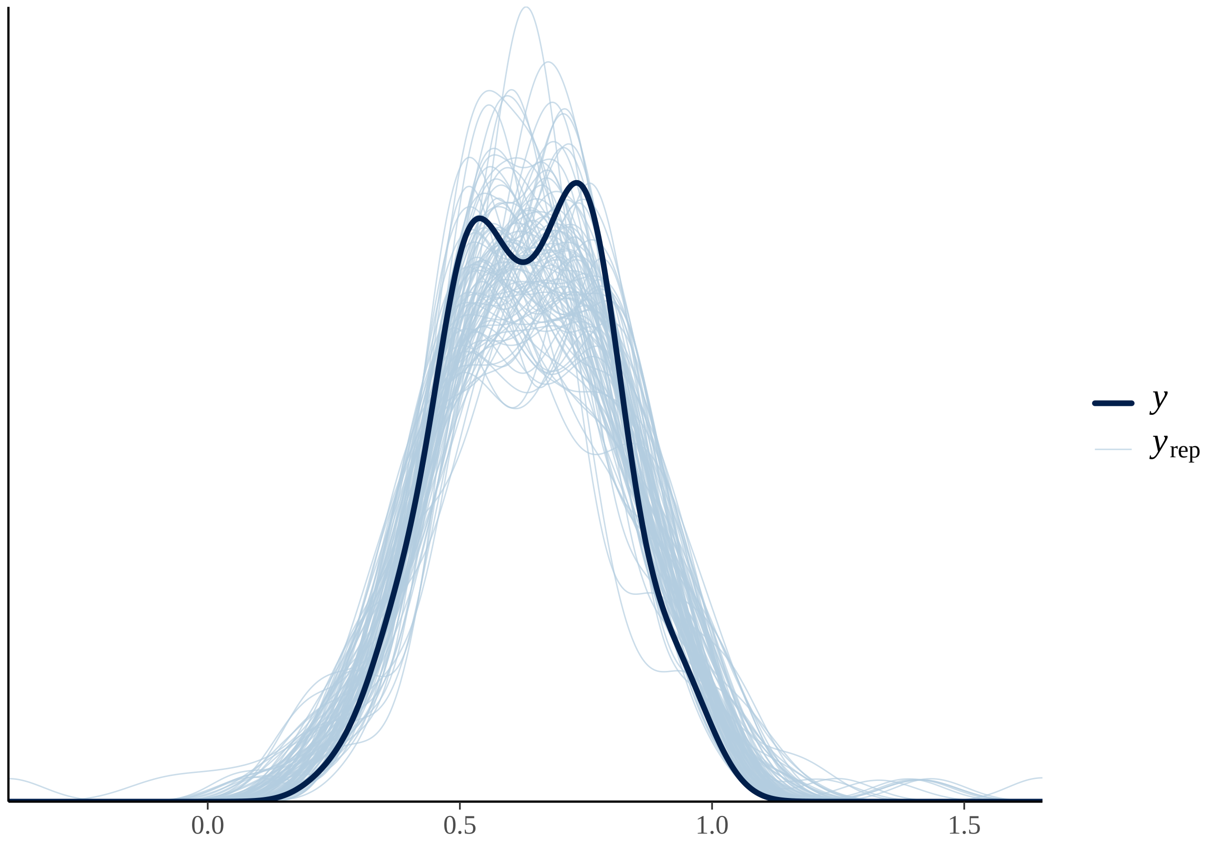

Fixed effects results TU-SH

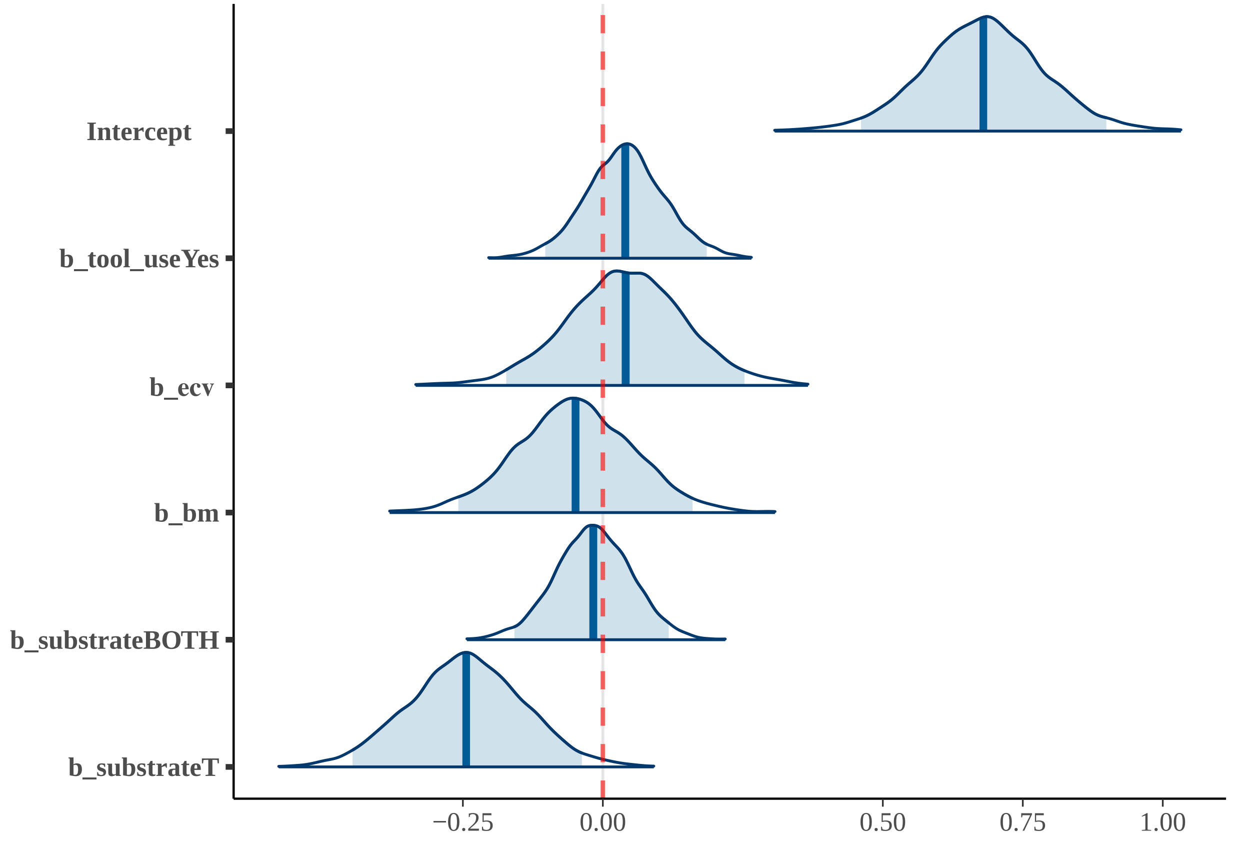

6) TU-BH

Summary TU-BH

effect	component	term	estimate	std.error	conf.low	conf.high	rhat
fixed	cond	(Intercept)	0.70	0.10	0.48	0.90	1
fixed	cond	tool_useYes	0.01	0.07	-0.13	0.16	1
fixed	cond	ecv	0.08	0.10	-0.13	0.28	1
fixed	cond	bm	-0.05	0.10	-0.25	0.15	1
fixed	cond	substrateBOTH	-0.07	0.08	-0.23	0.09	1
fixed	cond	substrateT	-0.30	0.11	-0.51	-0.08	1
fixed	cond	imi	-0.08	0.05	-0.17	0.03	1

effect	component	group	term	estimate	std.error	conf.low	conf.high	rhat
ran_pars	cond	obs	sd__(Intercept)	0.10	0.02	0.06	0.13	1
ran_pars	cond	phylo	sd__(Intercept)	0.03	0.01	0.01	0.05	1
ran_pars	cond	species	sd__(Intercept)	0.03	0.03	0.00	0.10	1

Posterior predictive check TU-BH; 100 simulated datasets

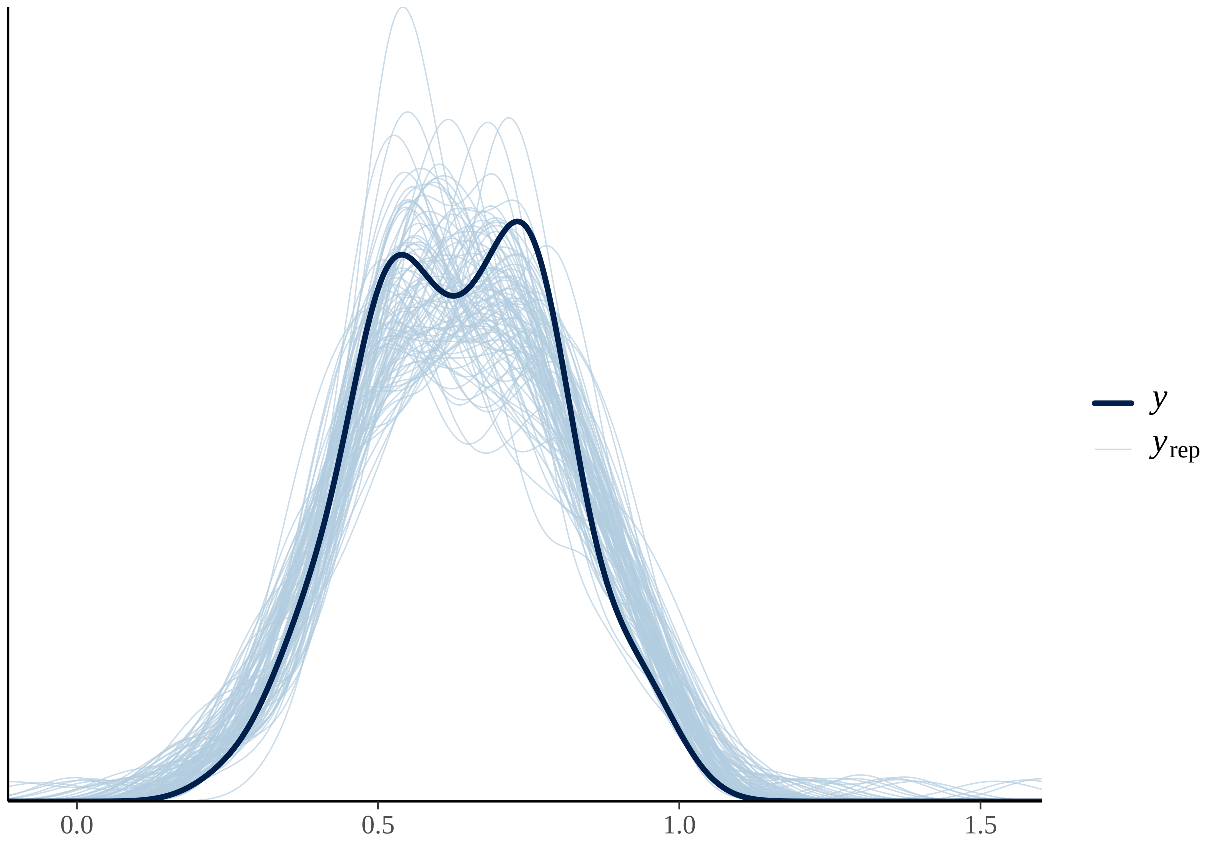

Fixed effects results TU-BH

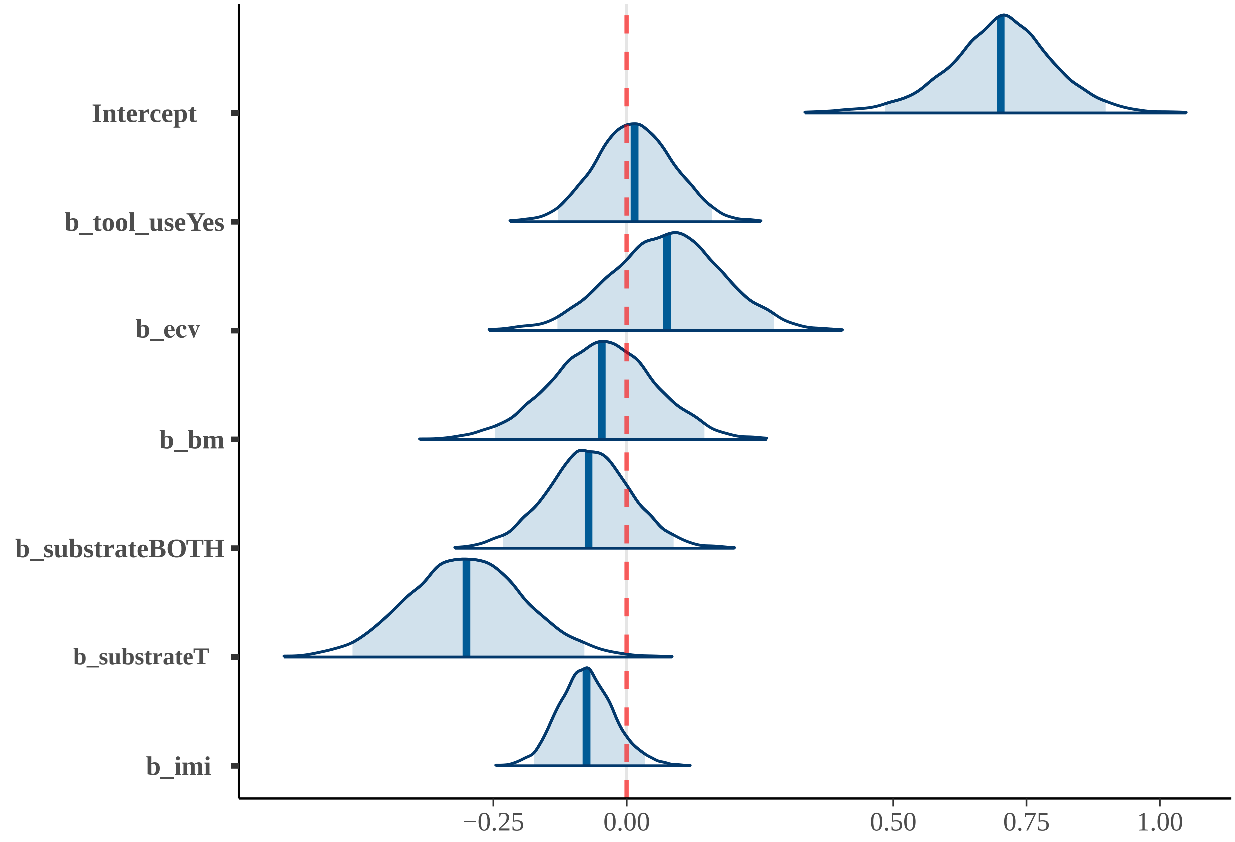

7) TU-SH-SSH

Summary TU-SH-SSH

effect	component	term	estimate	std.error	conf.low	conf.high	rhat
fixed	cond	(Intercept)	0.75	0.09	0.55	0.92	1
fixed	cond	tool_useYes	0.03	0.07	-0.10	0.17	1
fixed	cond	substrateBOTH	-0.09	0.07	-0.23	0.05	1
fixed	cond	substrateT	-0.32	0.10	-0.51	-0.13	1
fixed	cond	social_systemPair	-0.19	0.10	-0.38	0.02	1
fixed	cond	social_systemSolitary	-0.21	0.16	-0.53	0.10	1

effect	component	group	term	estimate	std.error	conf.low	conf.high	rhat
ran_pars	cond	obs	sd__(Intercept)	0.10	0.02	0.07	0.14	1
ran_pars	cond	phylo	sd__(Intercept)	0.02	0.01	0.00	0.05	1
ran_pars	cond	species	sd__(Intercept)	0.03	0.03	0.00	0.09	1

Posterior predictive check TU-SH-SSH; 100 simulated datasets

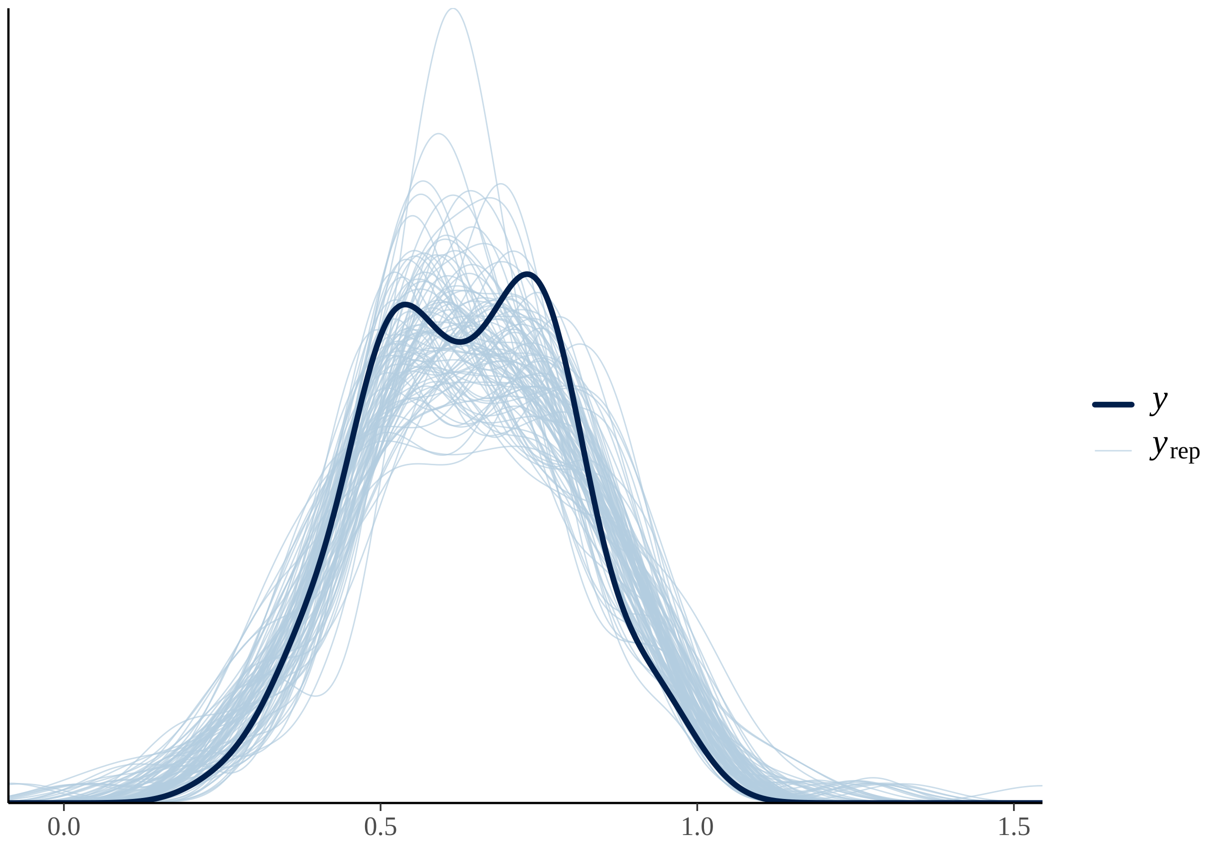

Fixed effects results TU-SH-SSH

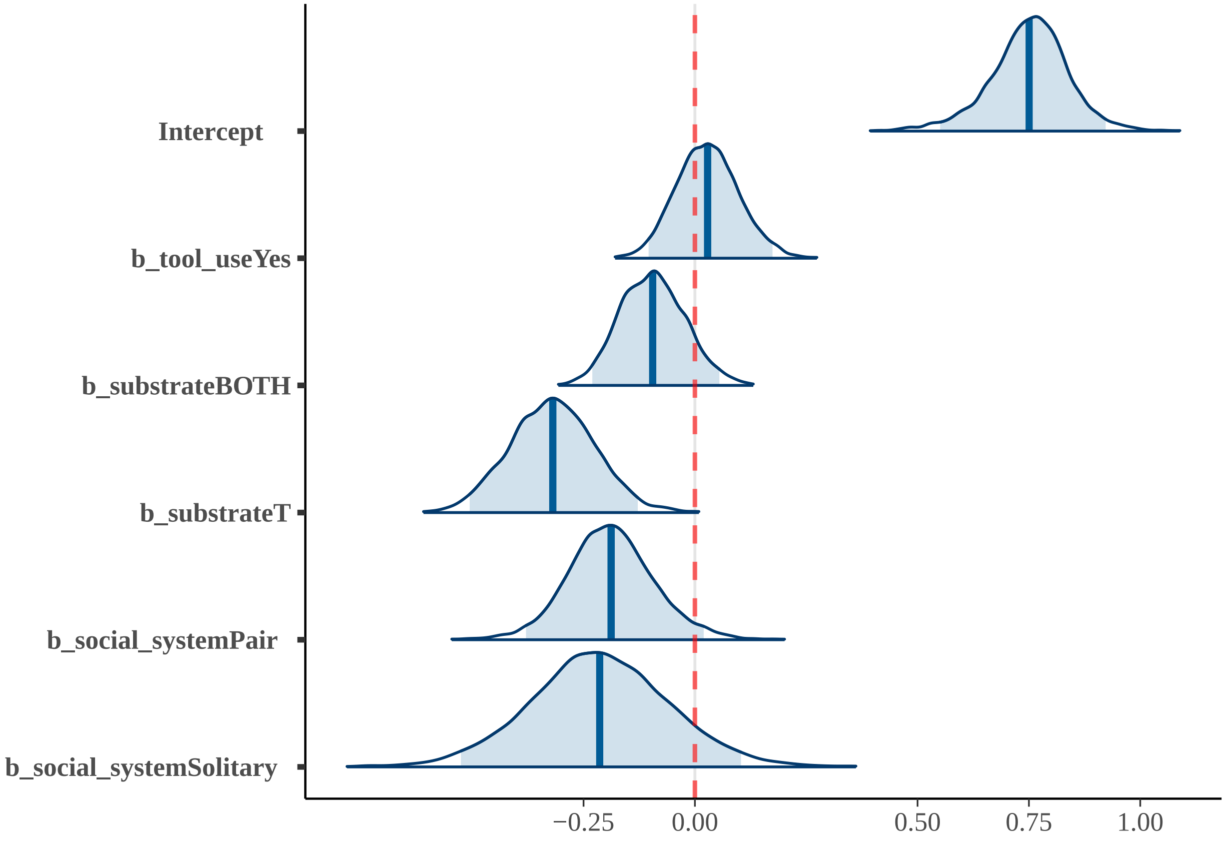

8) SP-SS-B-TUH

Summary SP-SS-B-TUH

effect	component	term	estimate	std.error	conf.low	conf.high	rhat
fixed	cond	(Intercept)	0.75	0.10	0.54	0.95	1
fixed	cond	tool_useYes	0.04	0.07	-0.10	0.18	1
fixed	cond	substrateBOTH	-0.06	0.08	-0.21	0.10	1
fixed	cond	substrateT	-0.26	0.11	-0.47	-0.05	1
fixed	cond	social_systemPair	-0.29	0.17	-0.63	0.05	1
fixed	cond	social_systemSolitary	-0.24	0.21	-0.66	0.17	1
fixed	cond	bm	-0.06	0.07	-0.20	0.07	1
fixed	cond	imi	0.04	0.08	-0.11	0.20	1

effect	component	group	term	estimate	std.error	conf.low	conf.high	rhat
ran_pars	cond	obs	sd__(Intercept)	0.09	0.02	0.06	0.13	1
ran_pars	cond	phylo	sd__(Intercept)	0.03	0.01	0.01	0.05	1
ran_pars	cond	species	sd__(Intercept)	0.03	0.02	0.00	0.09	1

Posterior predictive check SP-SS-B-TUH; 100 simulated datasets

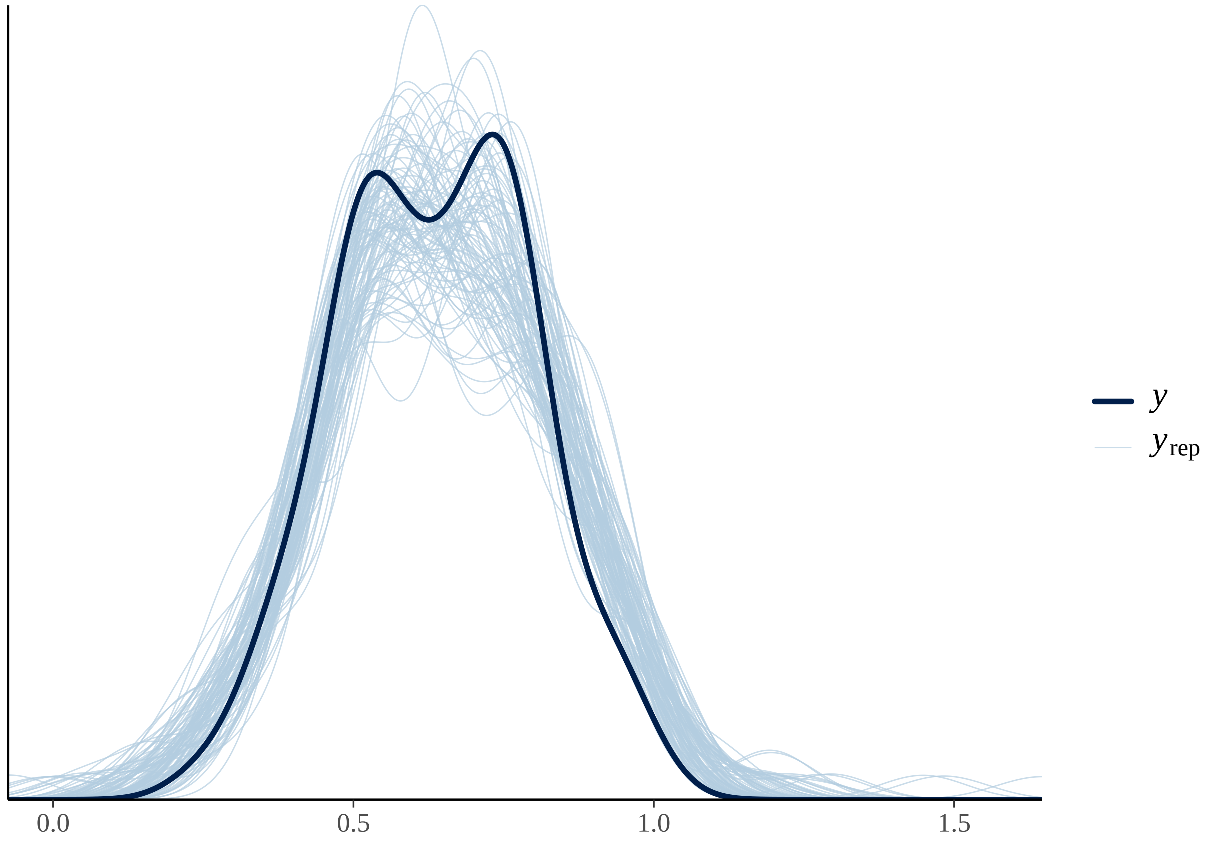

Fixed effects results SP-SS-B-TUH

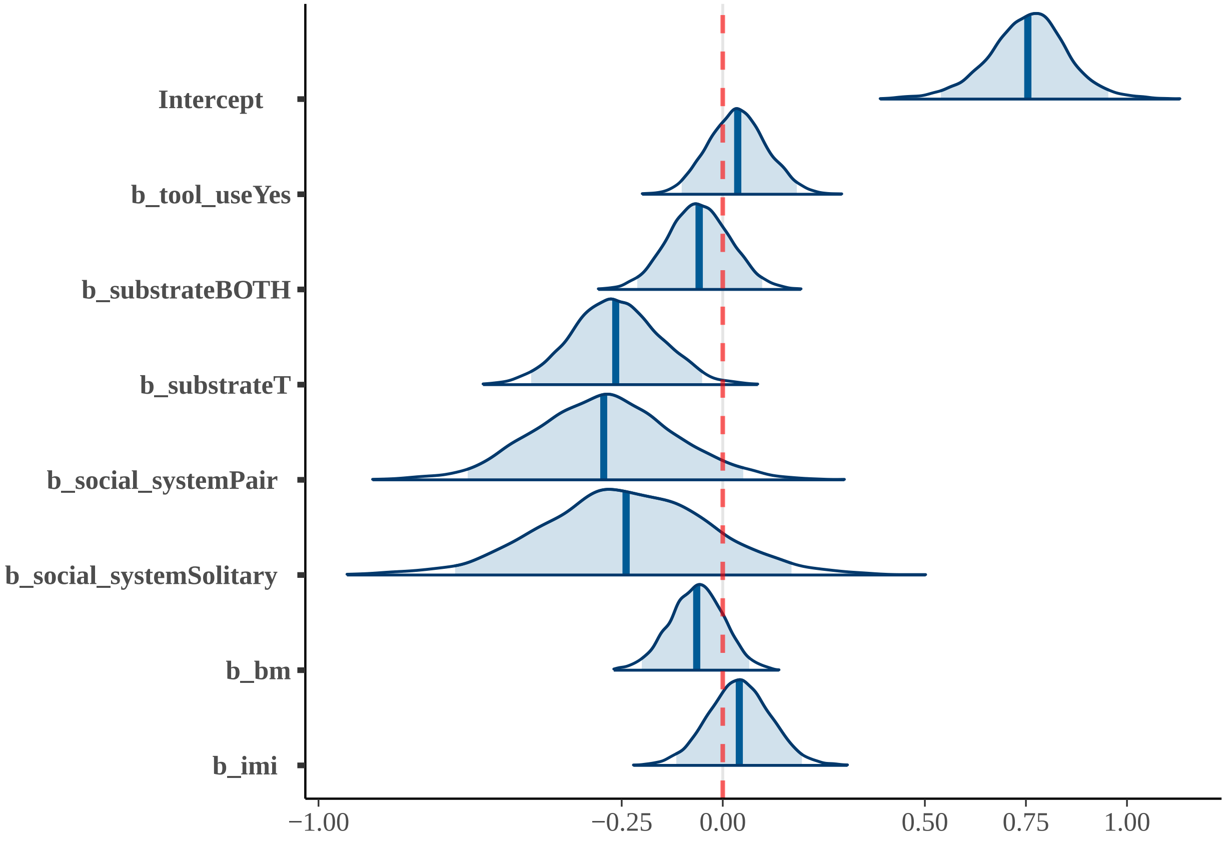

9) FH

Summary FH

effect	component	term	estimate	std.error	conf.low	conf.high	rhat
fixed	cond	(Intercept)	0.67	0.14	0.40	0.96	1
fixed	cond	dimo	-0.07	0.04	-0.14	0.00	1
fixed	cond	cl2	-0.09	0.13	-0.35	0.15	1
fixed	cond	cl3	0.08	0.14	-0.20	0.35	1
fixed	cond	cl4	-0.04	0.15	-0.35	0.24	1

effect	component	group	term	estimate	std.error	conf.low	conf.high	rhat
ran_pars	cond	obs	sd__(Intercept)	0.10	0.02	0.06	0.14	1
ran_pars	cond	phylo	sd__(Intercept)	0.03	0.01	0.01	0.05	1
ran_pars	cond	species	sd__(Intercept)	0.04	0.03	0.00	0.10	1

Posterior predictive check FH; 100 simulated datasets

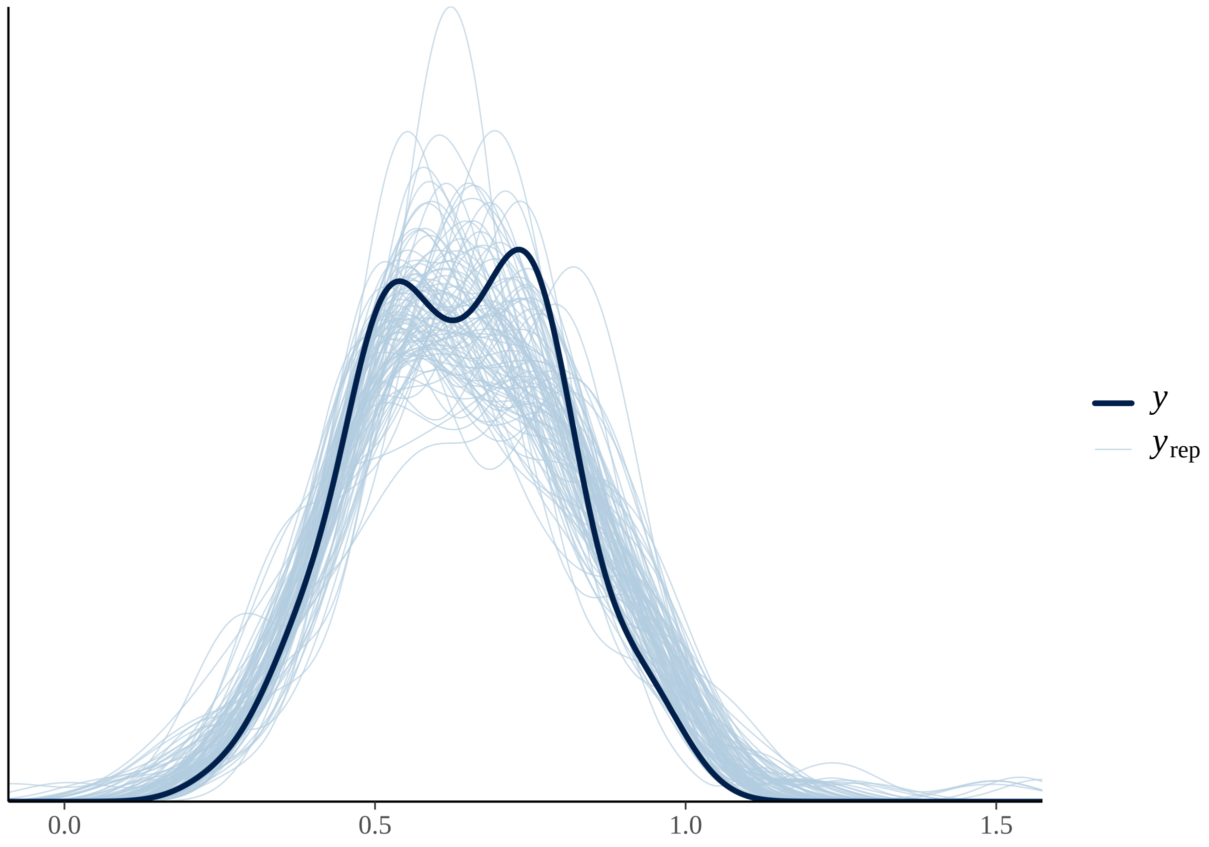

Fixed effects results FH

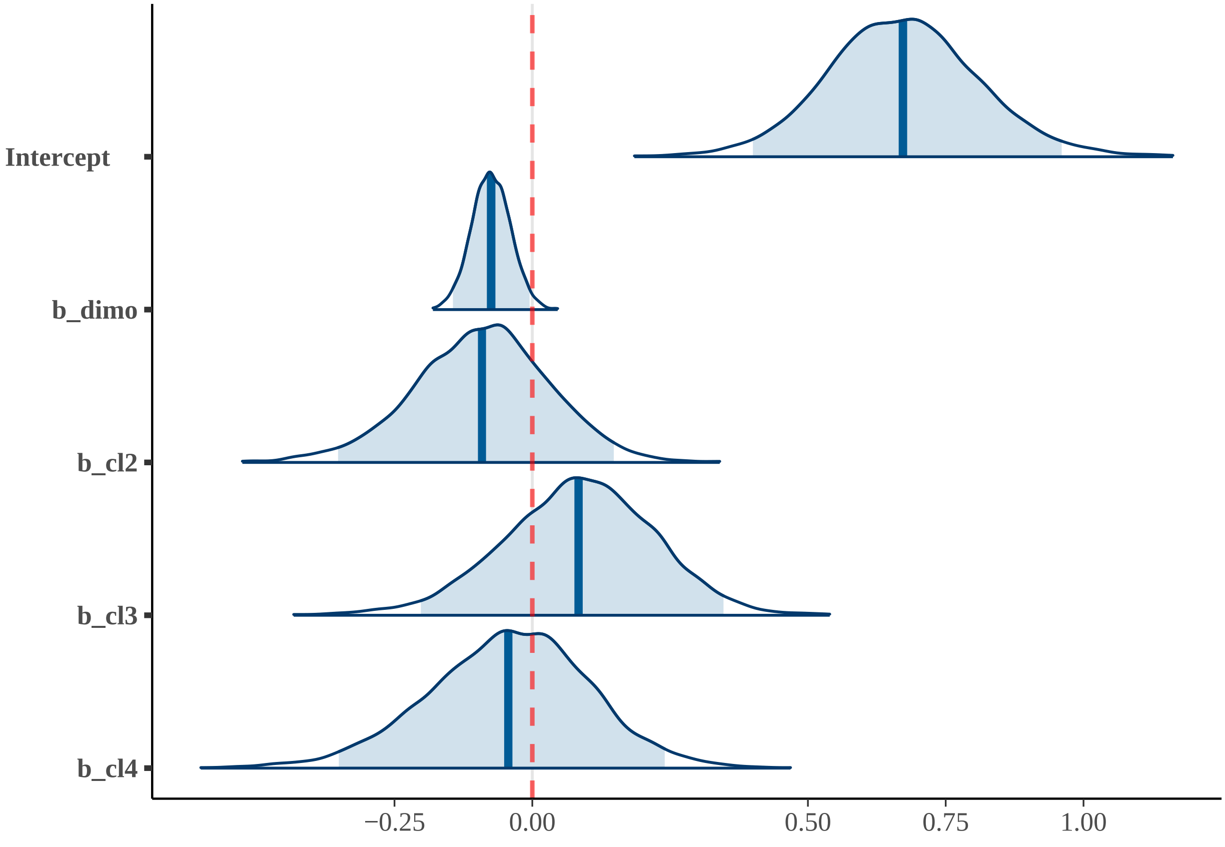

10) EFH

Summary EFH

effect	component	term	estimate	std.error	conf.low	conf.high	rhat
fixed	cond	(Intercept)	0.68	0.11	0.45	0.90	1
fixed	cond	extractive	-0.05	0.08	-0.21	0.12	1
fixed	cond	social_learning	-0.02	0.07	-0.16	0.12	1
fixed	cond	tool_useYes	0.08	0.08	-0.07	0.23	1

effect	component	group	term	estimate	std.error	conf.low	conf.high	rhat
ran_pars	cond	obs	sd__(Intercept)	0.10	0.02	0.07	0.14	1
ran_pars	cond	phylo	sd__(Intercept)	0.03	0.01	0.01	0.06	1
ran_pars	cond	species	sd__(Intercept)	0.04	0.03	0.00	0.11	1

Posterior predictive check EFH; 100 simulated datasets

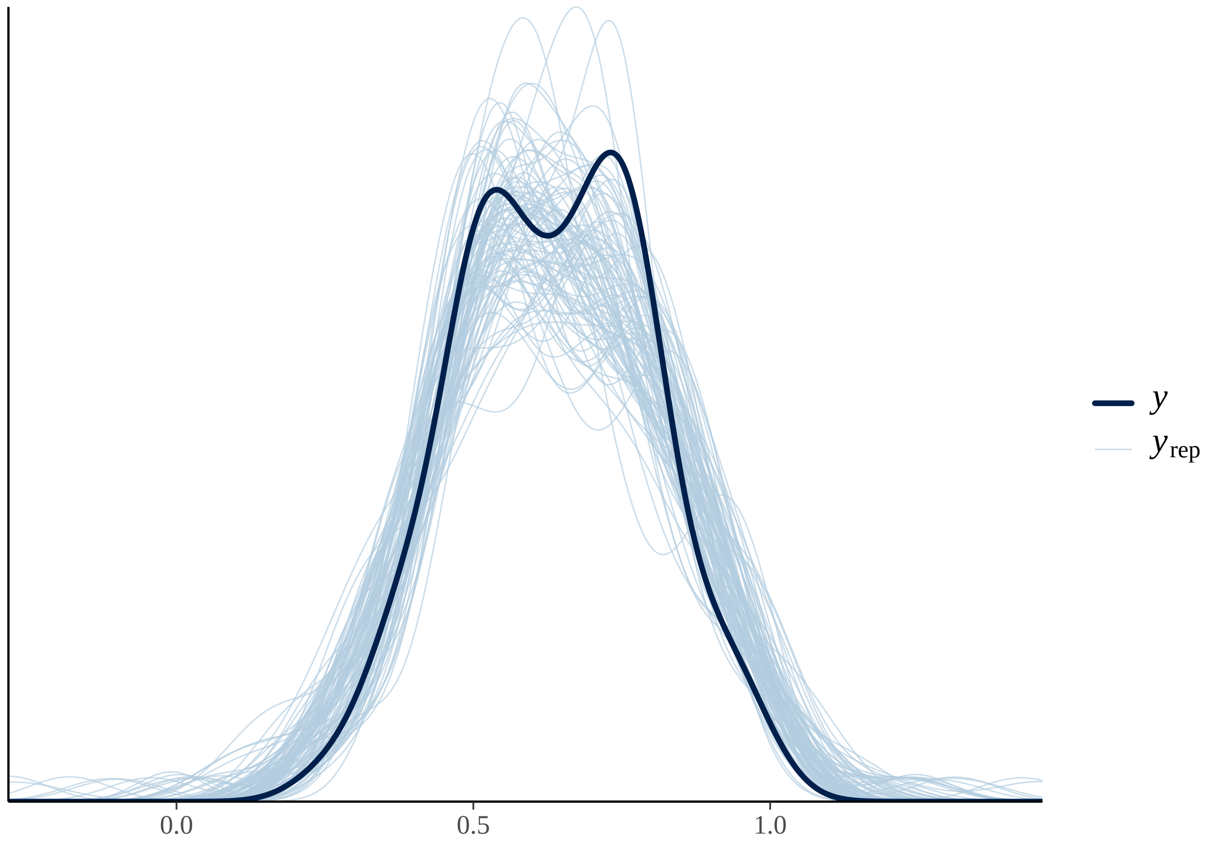

Fixed effects results EFH

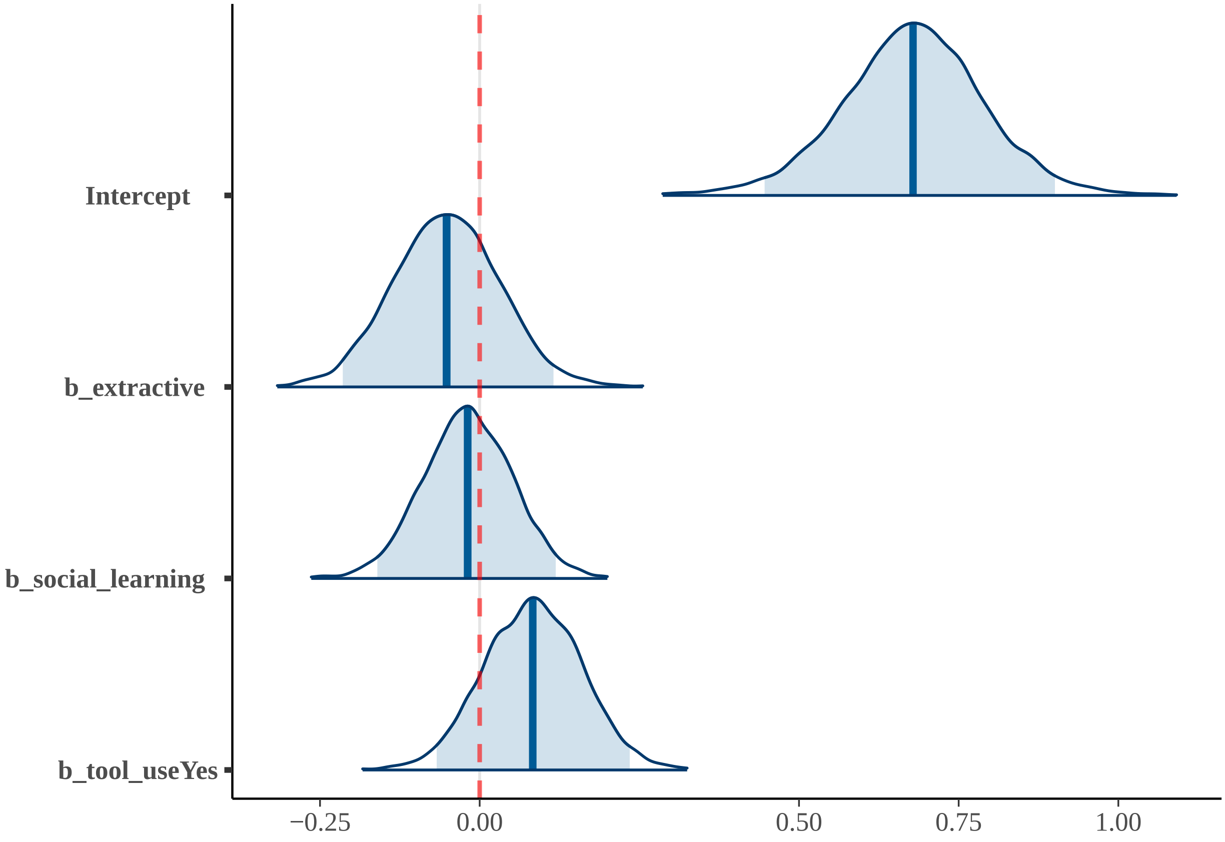

11) Reduced model

Summary Reduced model

effect	component	term	estimate	std.error	conf.low	conf.high	rhat
fixed	cond	(Intercept)	0.69	0.10	0.50	0.88	1
fixed	cond	substrateBOTH	-0.02	0.06	-0.15	0.10	1
fixed	cond	substrateT	-0.26	0.10	-0.44	-0.07	1

effect	component	group	term	estimate	std.error	conf.low	conf.high	rhat
ran_pars	cond	obs	sd___(Intercept)	0.10	0.02	0.06	0.13	1
ran_pars	cond	phylo	sd___(Intercept)	0.03	0.01	0.01	0.05	1
ran_pars	cond	species	sd___(Intercept)	0.03	0.02	0.00	0.09	1

Posterior predictive check Reduced model; 100 simulated datasets

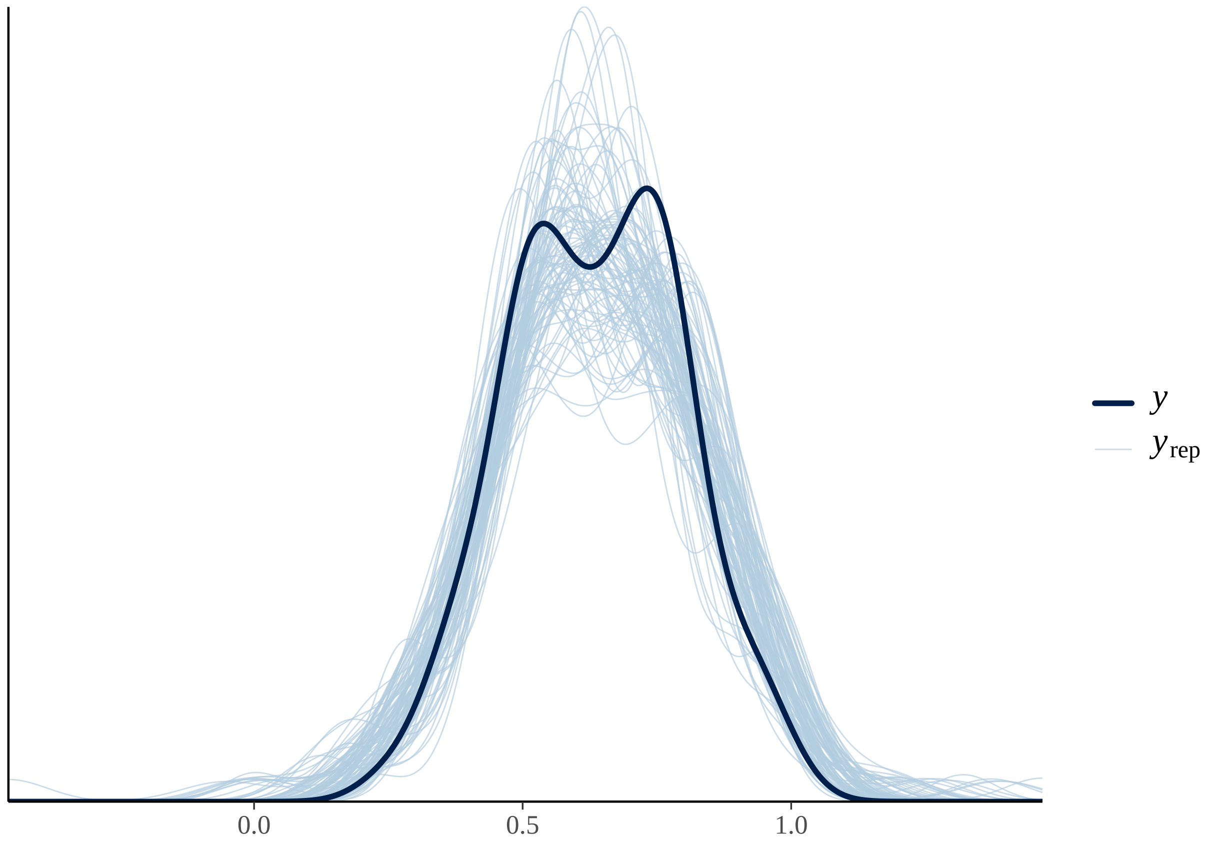

Fixed effects results Reduced model

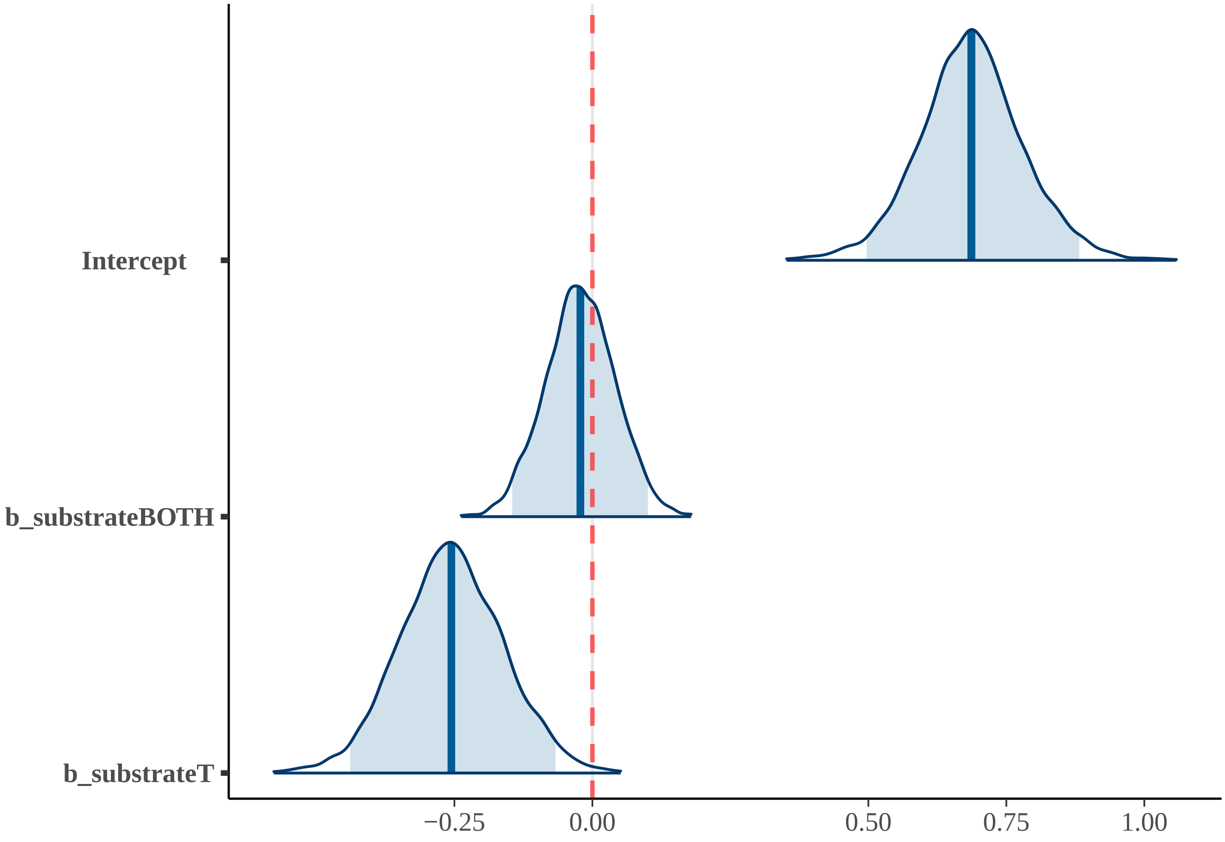

MABSHI results including *Homo sapiens*

Numerical results for all the hypotheses tested, using MABSHI as the dependent variable, are presented here (see Supplementary Table 1 for further details). *Homo sapiens* was included in these analyses. In addition, the final reduced model is also presented after all the hypotheses tested. Posterior predictive checks for each of the tested hypotheses are shown, along with plots illustrating the estimates for the fixed effects included in each of the tested models.

0) Intercepts-only model

Summary

effect	component	term	estimate	std.error	conf.low	conf.high	rhat
fixed	cond	(Intercept)	0.67	0.11	0.44	0.91	1

effect	component	group	term	estimate	std.error	conf.low	conf.high	rhat
ran_pars	cond	obs	sd__(Intercept)	0.11	0.02	0.07	0.15	1
ran_pars	cond	phylo	sd__(Intercept)	0.03	0.01	0.01	0.06	1
ran_pars	cond	species	sd__(Intercept)	0.04	0.03	0.00	0.12	1

Posterior predictive check; 100 simulated datasets

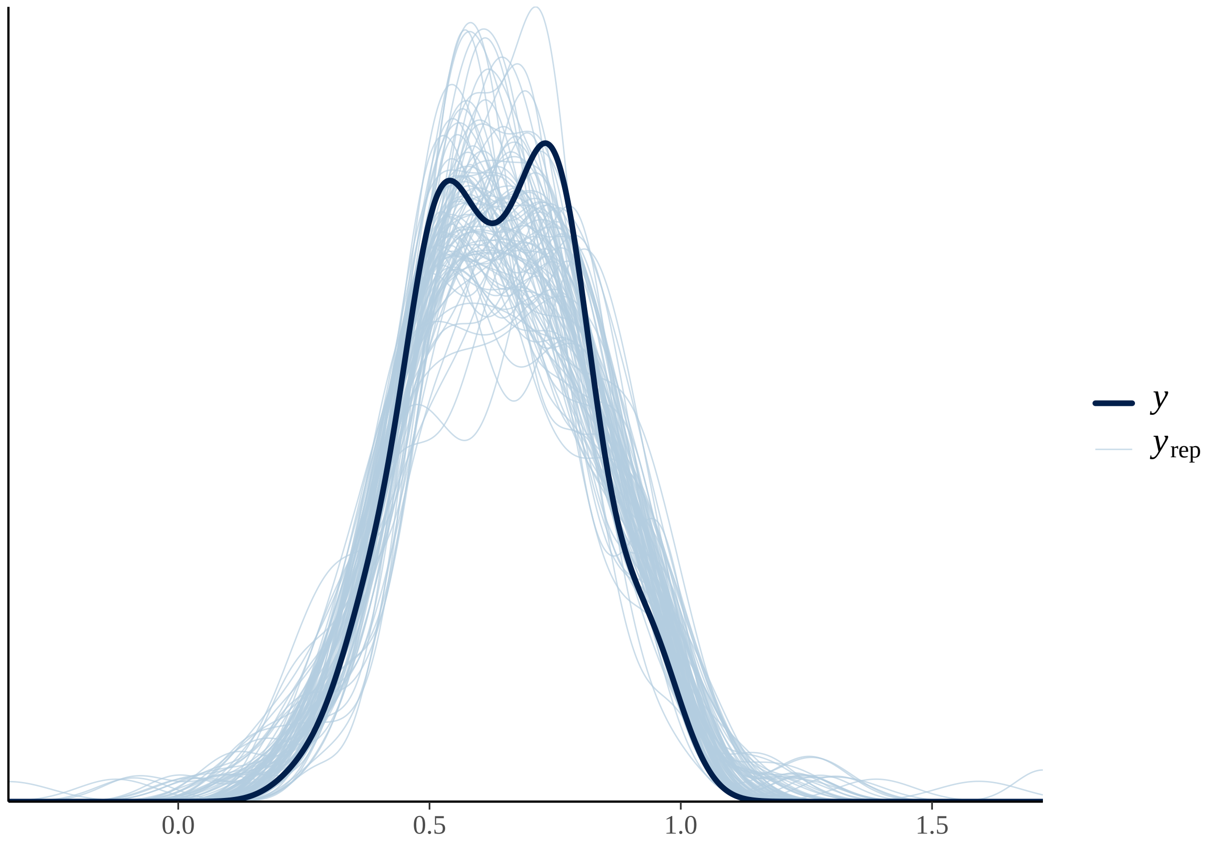

Fixed effects results

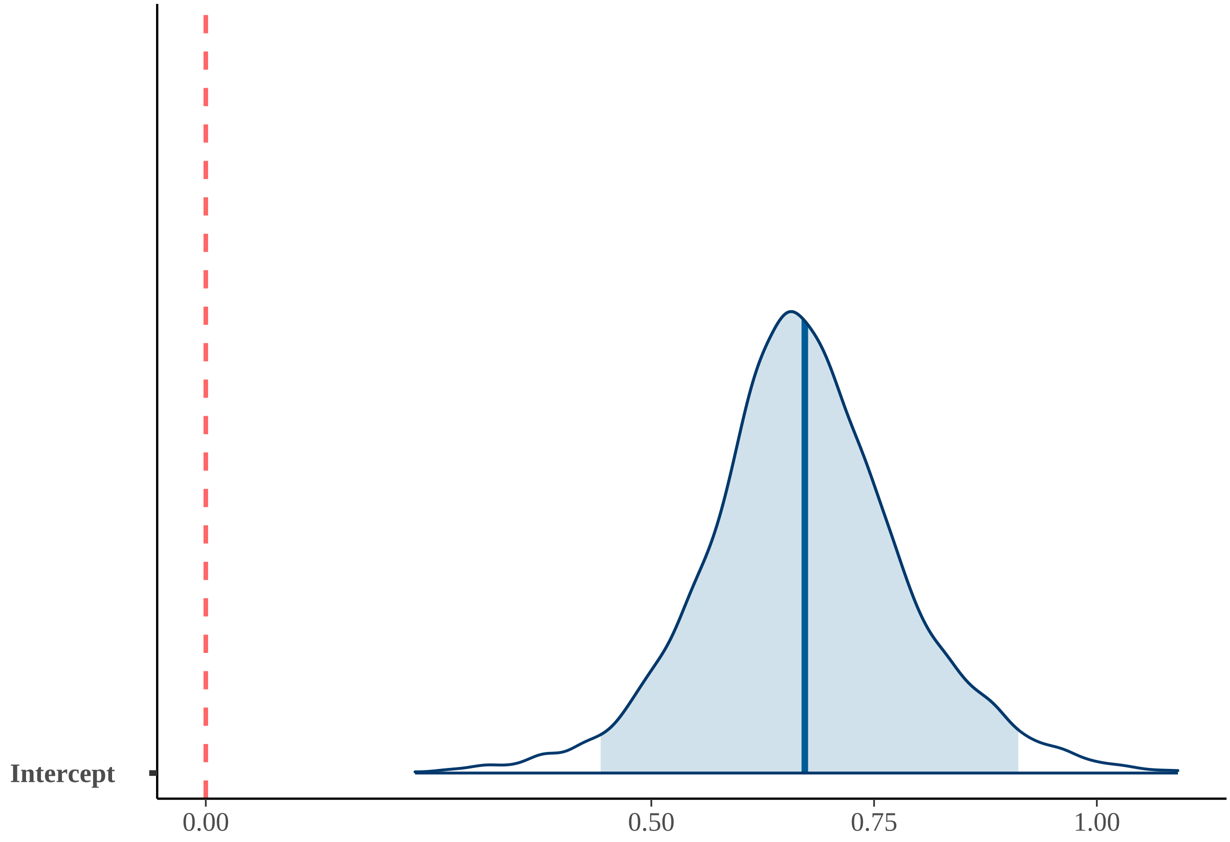

1) POH1

Summary POH1

effect	component	term	estimate	std.error	conf.low	conf.high	rhat
fixed	cond	(Intercept)	0.67	0.12	0.44	0.92	1
fixed	cond	fru	0.02	0.02	-0.02	0.07	1
fixed	cond	substrateBOTH	-0.02	0.07	-0.16	0.13	1
fixed	cond	substrateT	-0.08	0.10	-0.28	0.12	1

effect	component	group	term	estimate	std.error	conf.low	conf.high	rhat
ran_pars	cond	obs	sd__(Intercept)	0.11	0.02	0.07	0.15	1
ran_pars	cond	phylo	sd__(Intercept)	0.03	0.01	0.01	0.06	1
ran_pars	cond	species	sd__(Intercept)	0.05	0.04	0.00	0.13	1

Posterior predictive check POH1; 100 simulated datasets

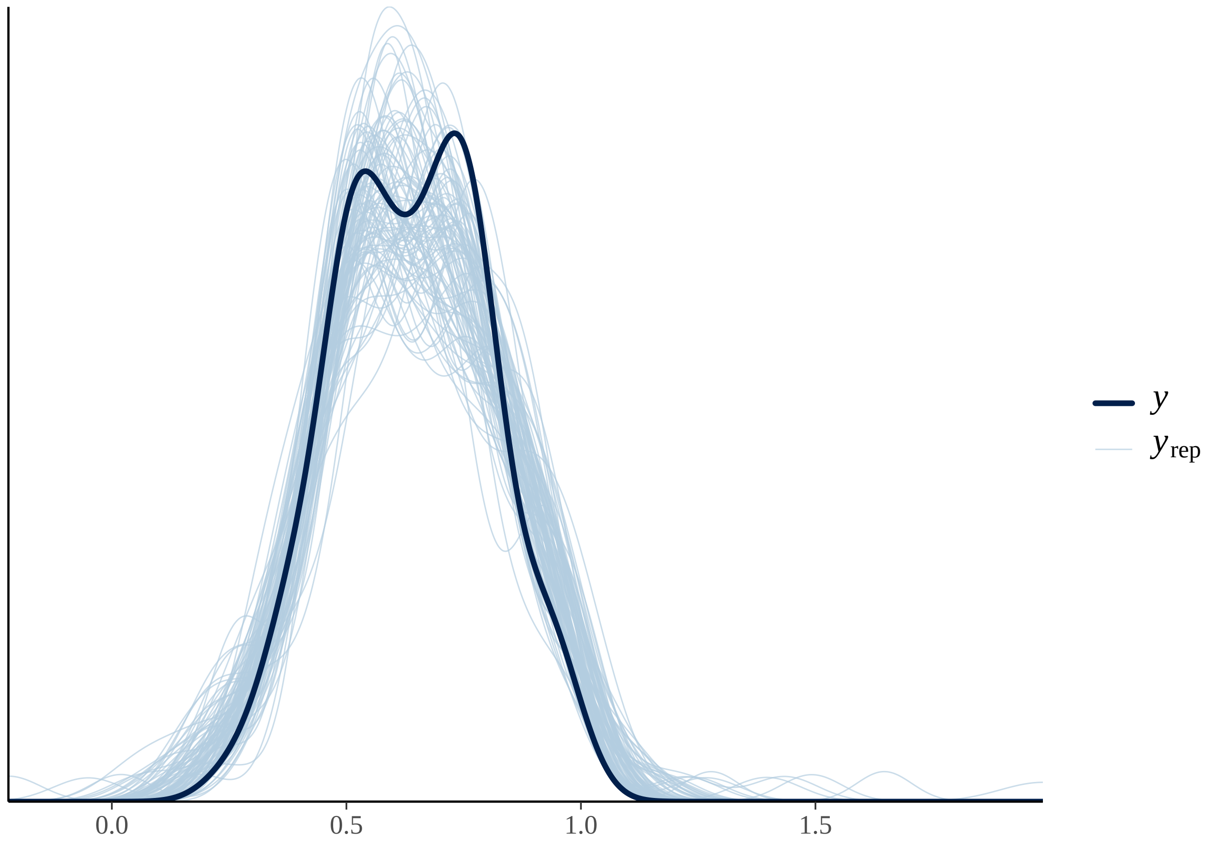

Fixed effects results POH1

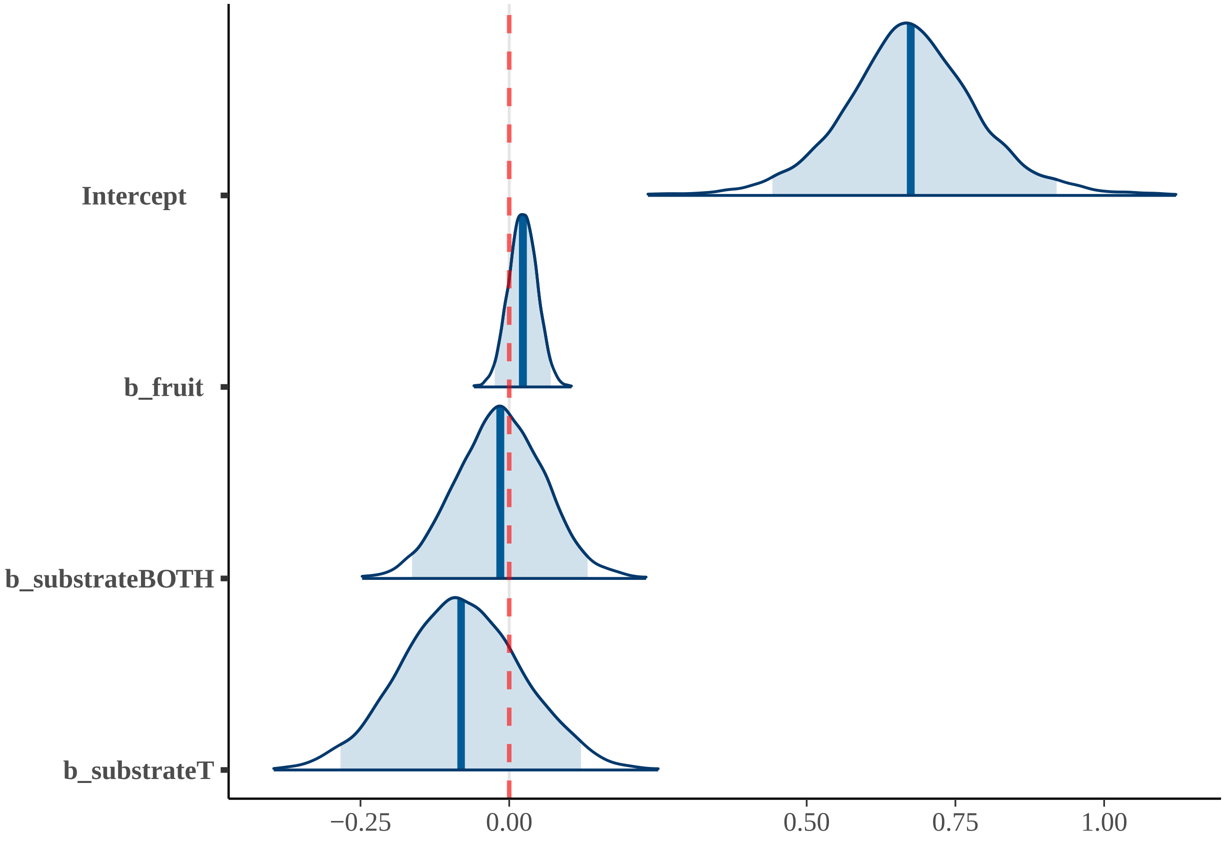

2) POH2

Summary POH2

effect	component	term	estimate	std.error	conf.low	conf.high	rhat
fixed	cond	(Intercept)	0.63	0.13	0.38	0.87	1
fixed	cond	diet	0.04	0.02	0.00	0.08	1
fixed	cond	substrateBOTH	-0.01	0.07	-0.15	0.14	1
fixed	cond	substrateT	-0.10	0.10	-0.29	0.09	1

effect	component	group	term	estimate	std.error	conf.low	conf.high	rhat
ran__pars	cond	obs	sd___(Intercept)	0.10	0.02	0.07	0.14	1
ran__pars	cond	phylo	sd___(Intercept)	0.04	0.01	0.02	0.06	1
ran__pars	cond	species	sd___(Intercept)	0.04	0.03	0.00	0.11	1

Posterior predictive check POH2; 100 simulated datasets

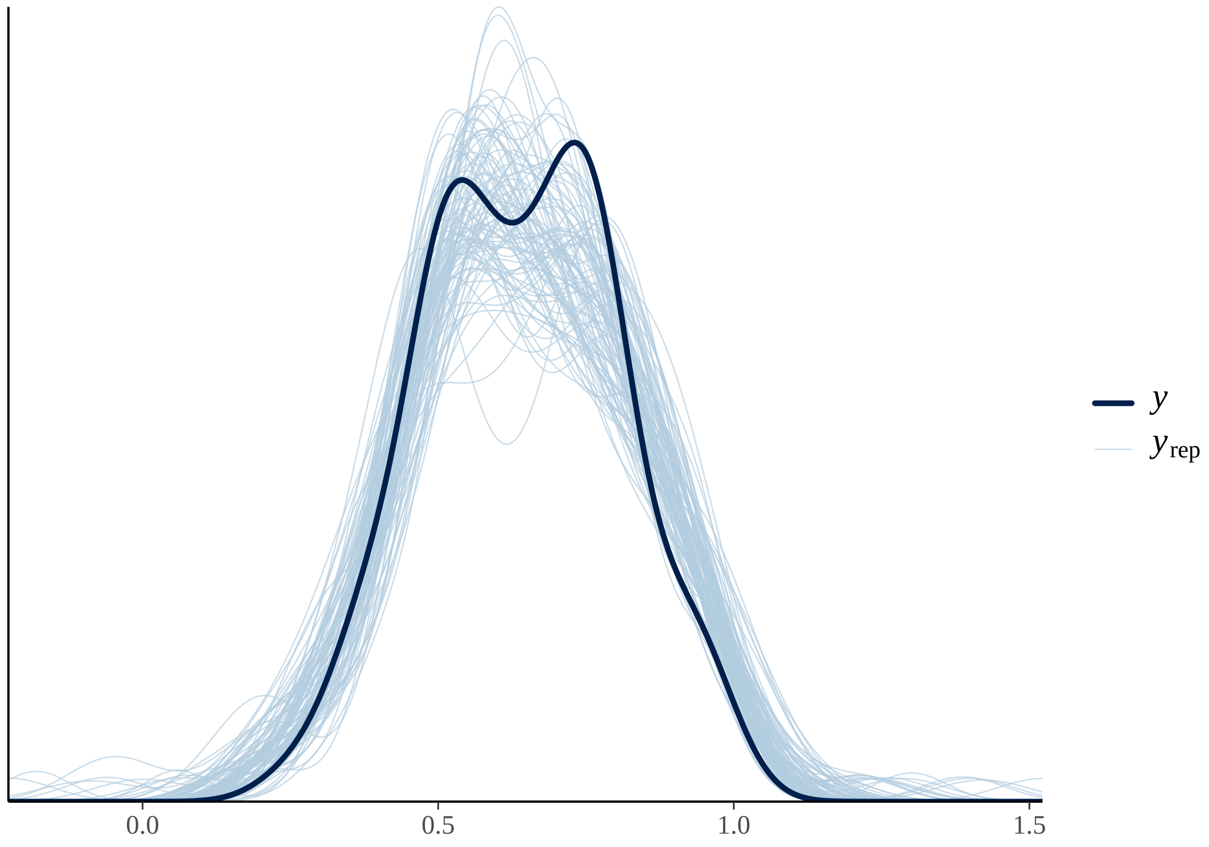

Fixed effects results POH2

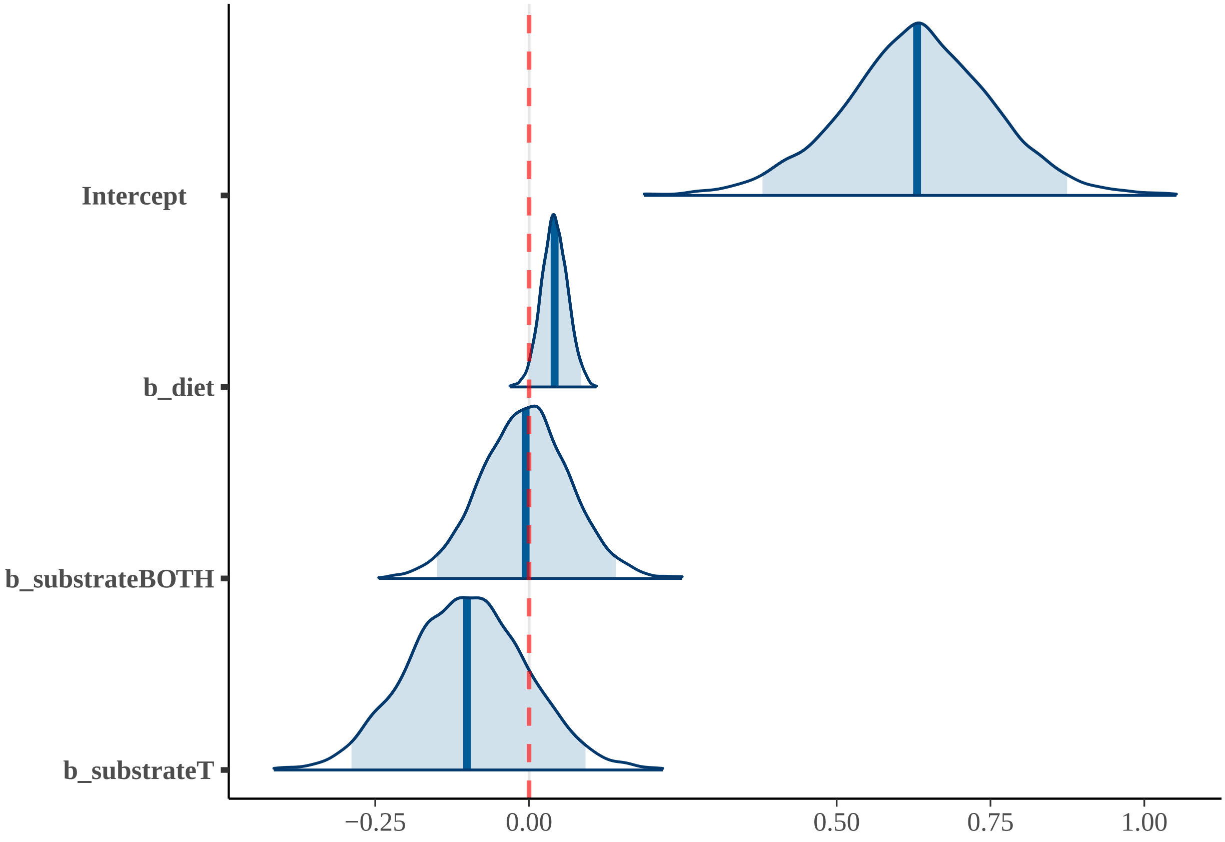

3) BH

Summary BH

effect	component	term	estimate	std.error	conf.low	conf.high	rhat
fixed	cond	(Intercept)	0.72	0.09	0.54	0.91	1
fixed	cond	imi2	-0.11	0.04	-0.19	-0.02	1
fixed	cond	cc	0.18	0.07	0.03	0.32	1
fixed	cond	bm	-0.12	0.08	-0.28	0.05	1
fixed	cond	substrateBOTH	-0.10	0.08	-0.25	0.05	1
fixed	cond	substrateT	-0.29	0.11	-0.50	-0.08	1

effect	component	group	term	estimate	std.error	conf.low	conf.high	rhat
ran_pars	cond	obs	sd___(Intercept)	0.10	0.02	0.07	0.13	1
ran_pars	cond	phylo	sd___(Intercept)	0.03	0.01	0.01	0.05	1
ran_pars	cond	species	sd___(Intercept)	0.04	0.03	0.00	0.10	1

Posterior predictive check BH; 100 simulated datasets

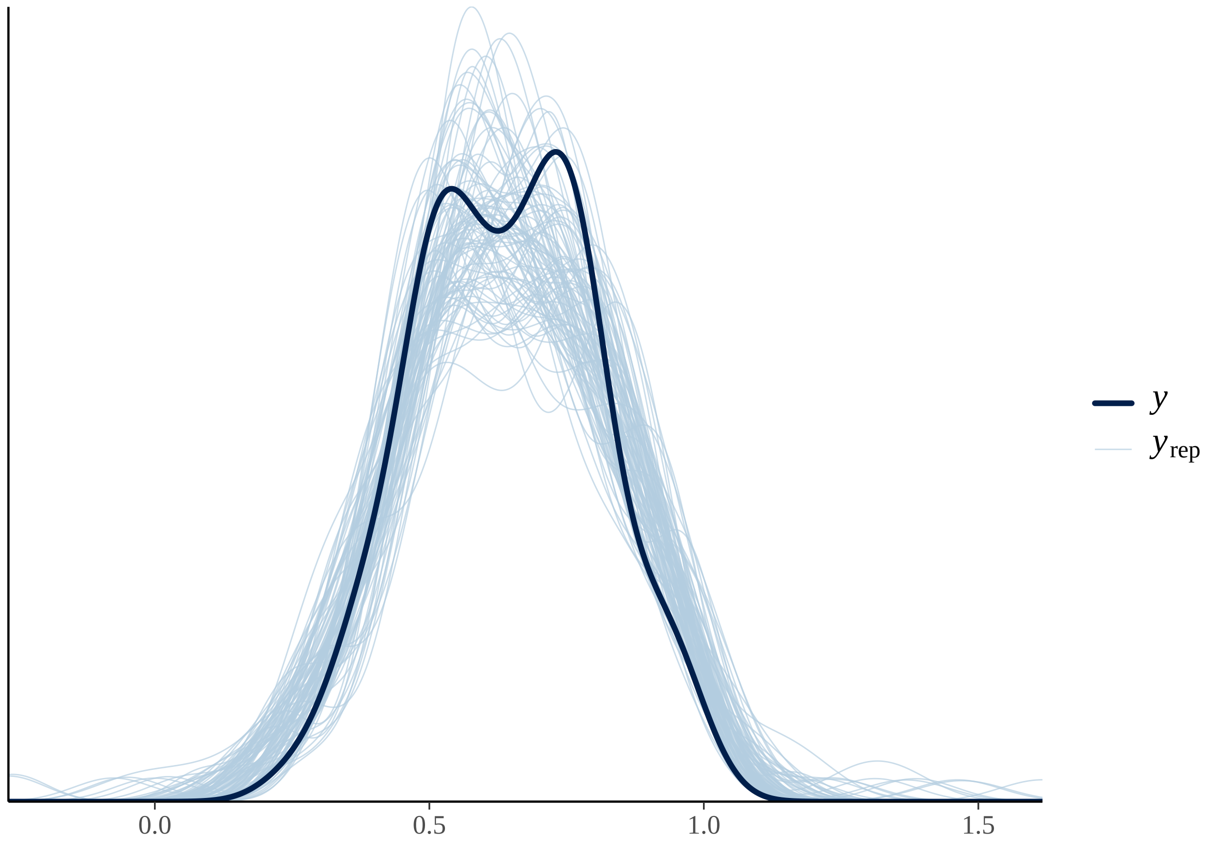

Fixed effects results BH

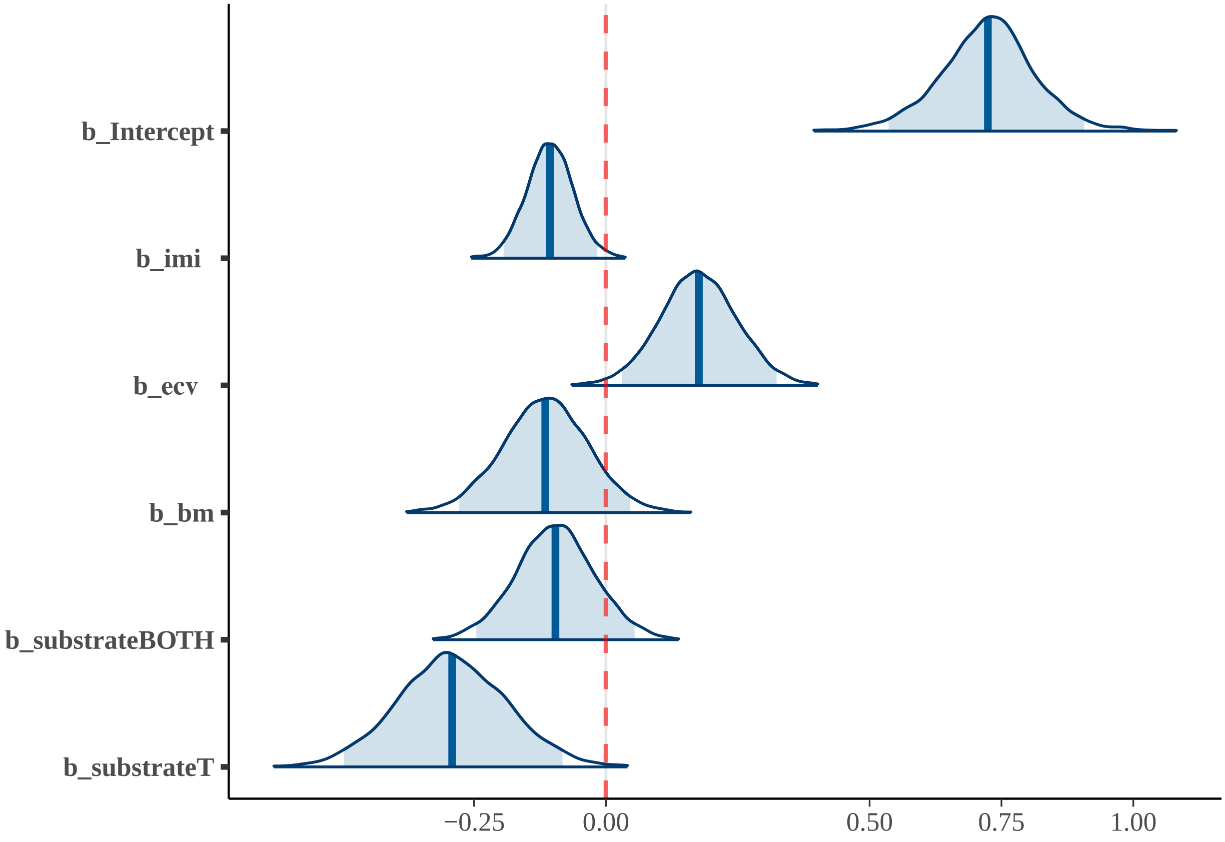

4) TUH

Summary TUH

effect	component	term	estimate	std.error	conf.low	conf.high	rhat
fixed	cond	(Intercept)	0.67	0.12	0.43	0.91	1
fixed	cond	tool_useYes	0.06	0.08	-0.09	0.21	1
fixed	cond	cc	0.15	0.08	0.00	0.31	1
fixed	cond	bm	-0.17	0.08	-0.34	-0.01	1

effect	component	group	term	estimate	std.error	conf.low	conf.high	rhat
ran_pars	cond	obs	sd__(Intercept)	0.10	0.02	0.07	0.14	1
ran_pars	cond	phylo	sd__(Intercept)	0.04	0.01	0.02	0.06	1
ran_pars	cond	species	sd__(Intercept)	0.04	0.03	0.00	0.10	1

Posterior predictive check TUH; 100 simulated datasets

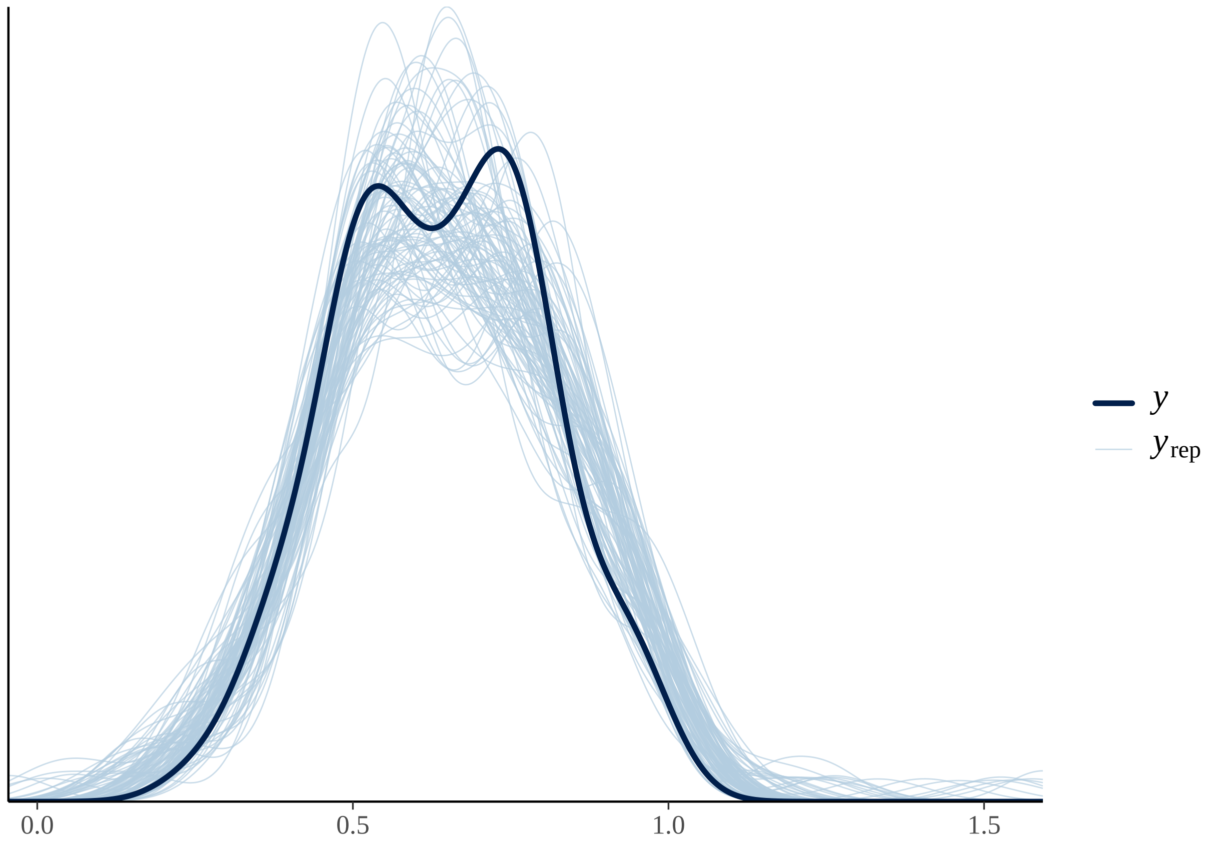

Fixed effects results TUH

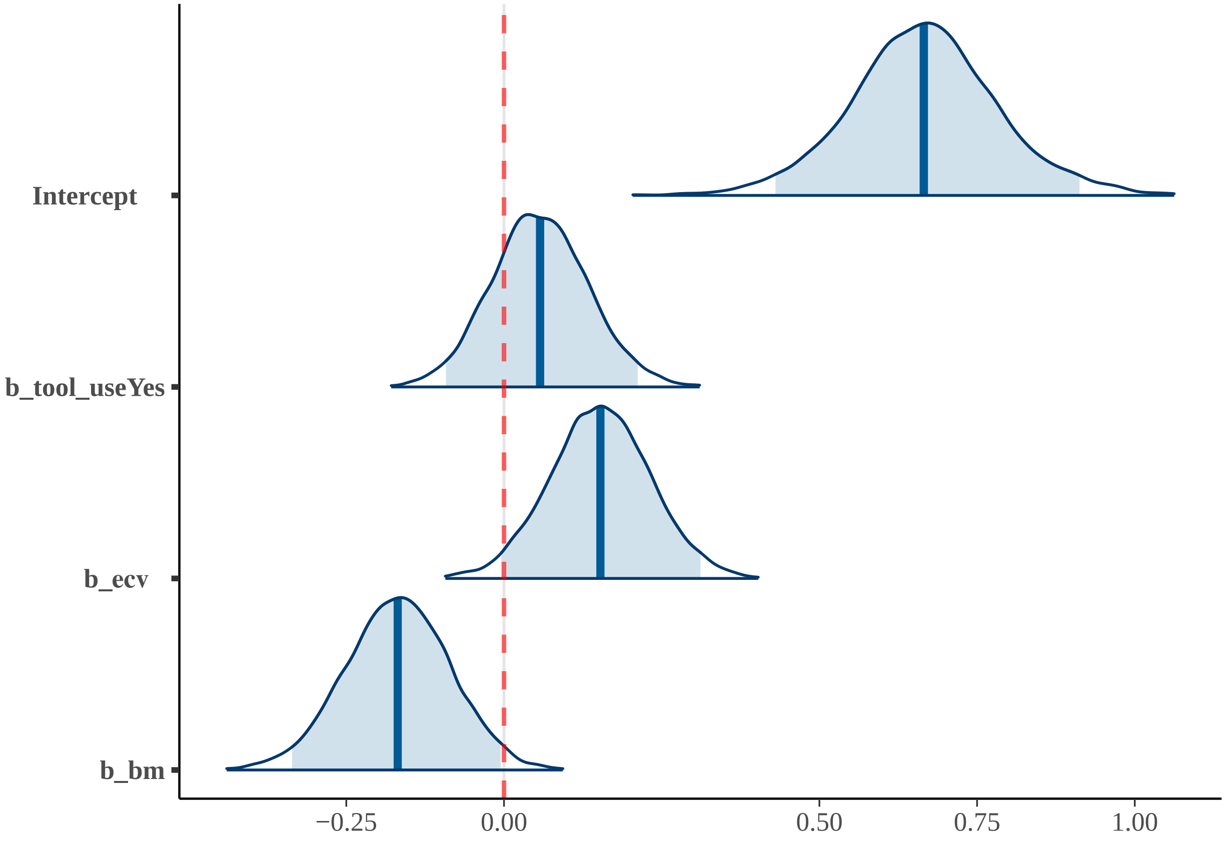

5) TU-SH

Summary TU-SH

effect	component	term	estimate	std.error	conf.low	conf.high	rhat
fixed	cond	(Intercept)	0.70	0.12	0.46	0.95	1
fixed	cond	tool_useYes	0.05	0.08	-0.10	0.20	1
fixed	cond	ecv	0.21	0.08	0.05	0.38	1
fixed	cond	bm	-0.20	0.08	-0.36	-0.03	1
fixed	cond	substrateBOTH	-0.03	0.07	-0.17	0.11	1
fixed	cond	substrateT	-0.18	0.11	-0.39	0.02	1

effect	component	group	term	estimate	std.error	conf.low	conf.high	rhat
ran_pars	cond	obs	sd__(Intercept)	0.09	0.02	0.06	0.13	1
ran_pars	cond	phylo	sd__(Intercept)	0.04	0.01	0.02	0.06	1
ran_pars	cond	species	sd__(Intercept)	0.04	0.03	0.00	0.10	1

Posterior predictive check TU-SH; 100 simulated datasets

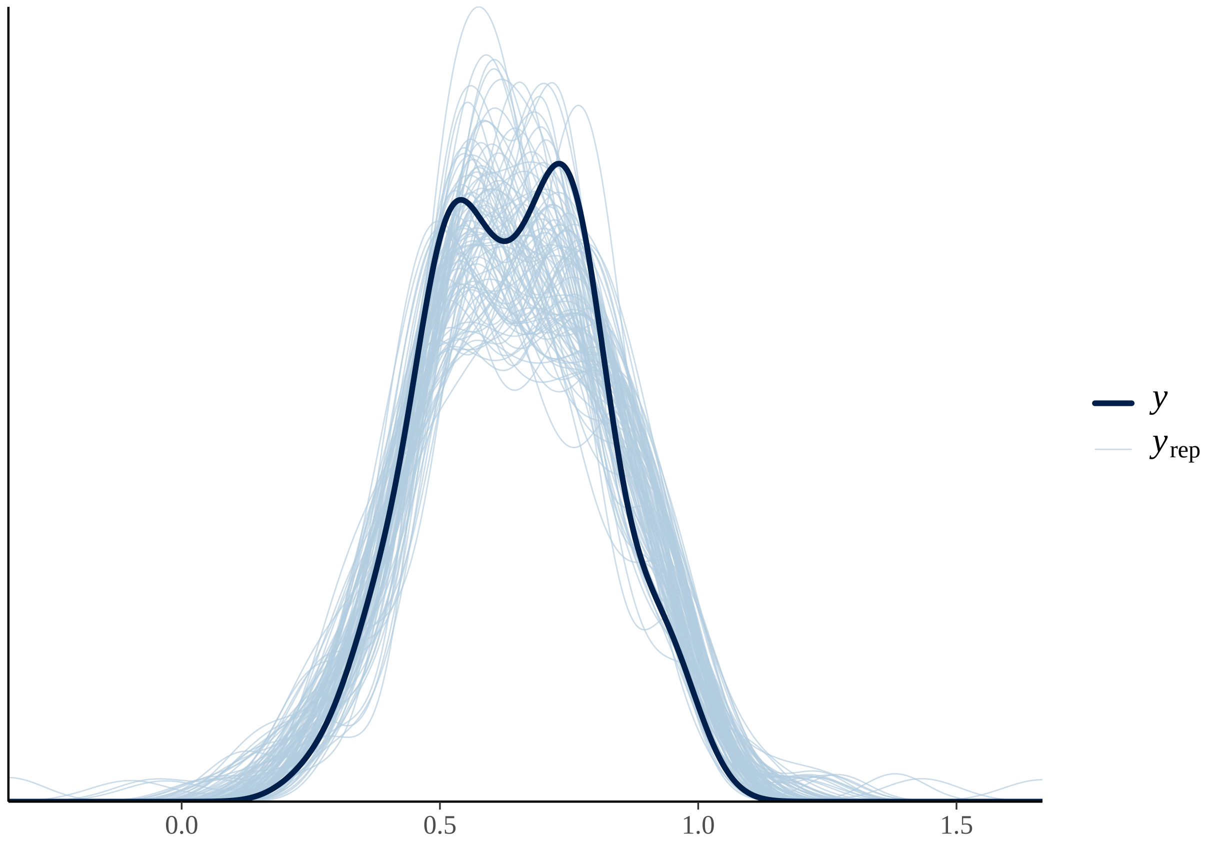

Fixed effects results TU-SH

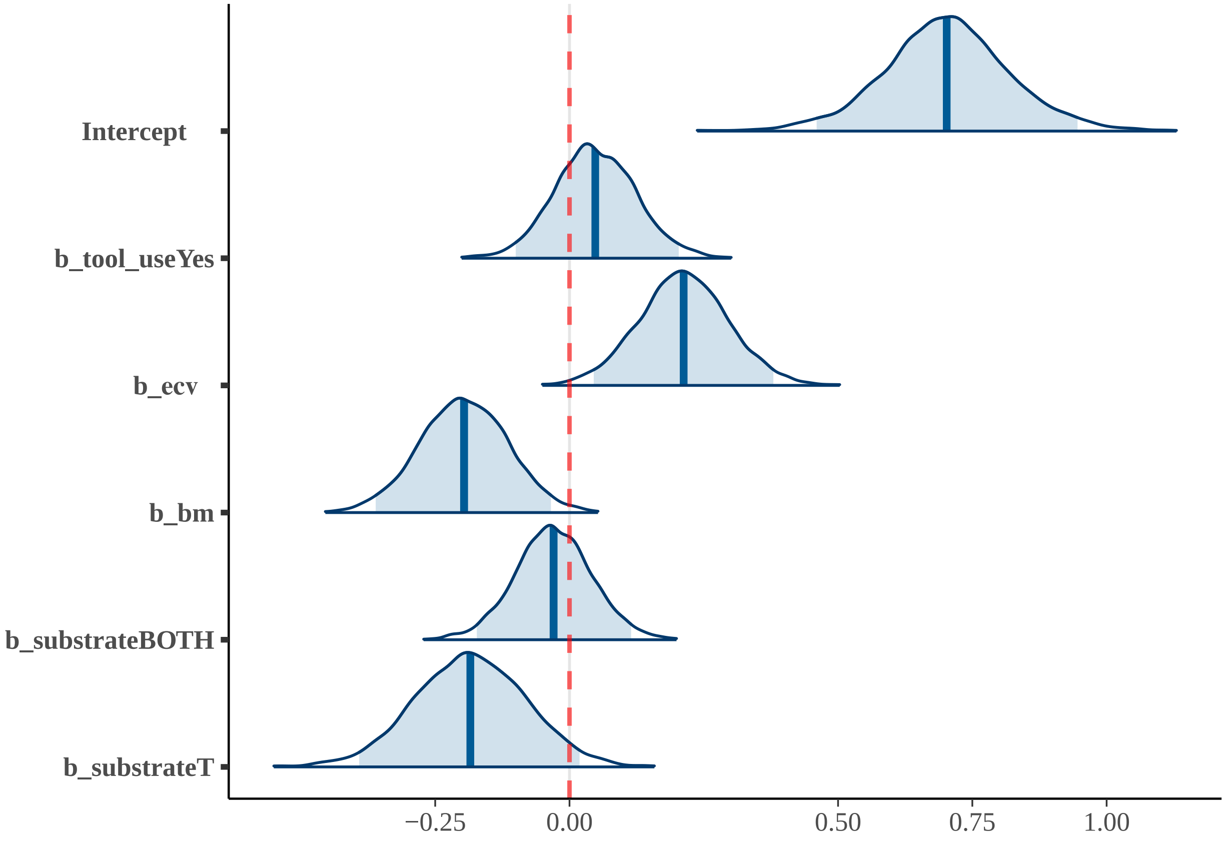

6) TU-BH

Summary TU-BH

effect	component	term	estimate	std.error	conf.low	conf.high	rhat
fixed	cond	(Intercept)	0.72	0.10	0.52	0.91	1
fixed	cond	tool_useYes	0.00	0.07	-0.14	0.15	1
fixed	cond	ecv	0.18	0.08	0.02	0.33	1
fixed	cond	bm	-0.12	0.08	-0.29	0.05	1
fixed	cond	substrateBOTH	-0.10	0.08	-0.25	0.06	1
fixed	cond	substrateT	-0.29	0.11	-0.50	-0.07	1
fixed	cond	imi	-0.11	0.05	-0.19	-0.01	1

effect	component	group	term	estimate	std.error	conf.low	conf.high	rhat
ran_pars	cond	obs	sd___(Intercept)	0.10	0.02	0.06	0.14	1
ran_pars	cond	phylo	sd___(Intercept)	0.03	0.01	0.00	0.05	1
ran_pars	cond	species	sd___(Intercept)	0.04	0.03	0.00	0.11	1

Posterior predictive check TU-BH; 100 simulated datasets

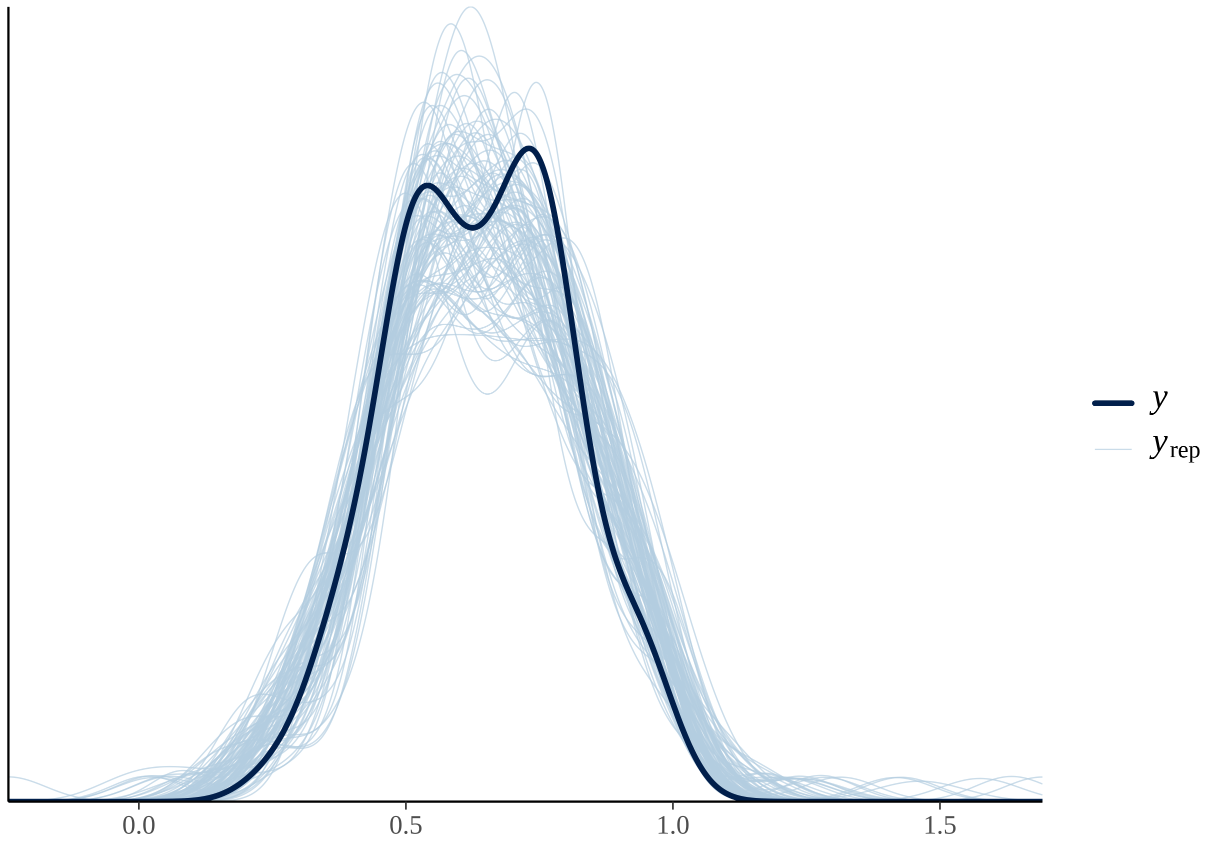

Fixed effects results TU-BH

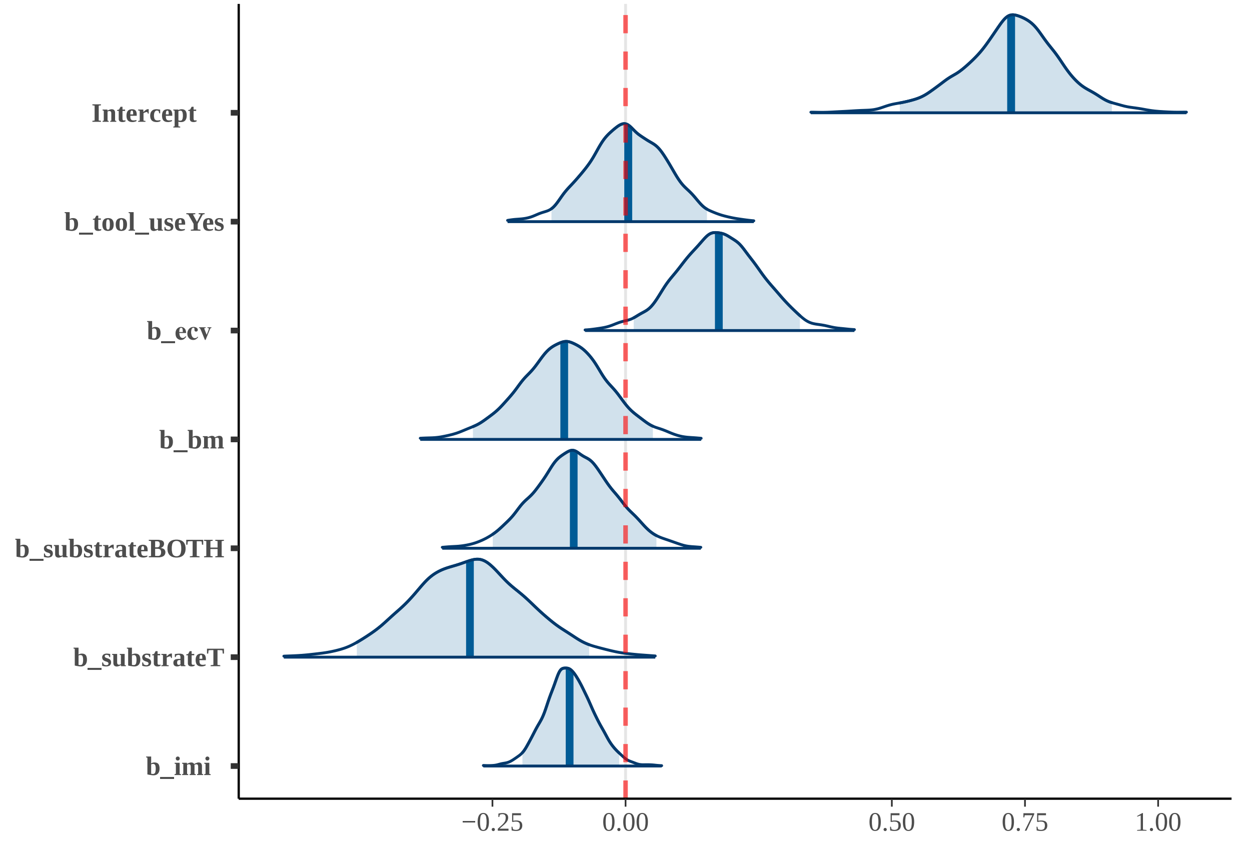

7) TU-SH-SSH

Summary TU-SH-SSH

effect	component	term	estimate	std.error	conf.low	conf.high	rhat
fixed	cond	(Intercept)	0.74	0.11	0.51	0.96	1
fixed	cond	tool_useYes	0.11	0.08	-0.04	0.27	1
fixed	cond	substrateBOTH	-0.09	0.08	-0.25	0.08	1
fixed	cond	substrateT	-0.19	0.10	-0.39	0.02	1
fixed	cond	social_systemPair	-0.19	0.12	-0.42	0.05	1
fixed	cond	social_systemSolitary	-0.30	0.19	-0.68	0.07	1

effect	component	group	term	estimate	std.error	conf.low	conf.high	rhat
ran_pars	cond	obs	sd__(Intercept)	0.10	0.02	0.07	0.15	1
ran_pars	cond	phylo	sd__(Intercept)	0.03	0.01	0.00	0.06	1
ran_pars	cond	species	sd__(Intercept)	0.05	0.03	0.00	0.12	1

Posterior predictive check TU-SH-SSH; 100 simulated datasets

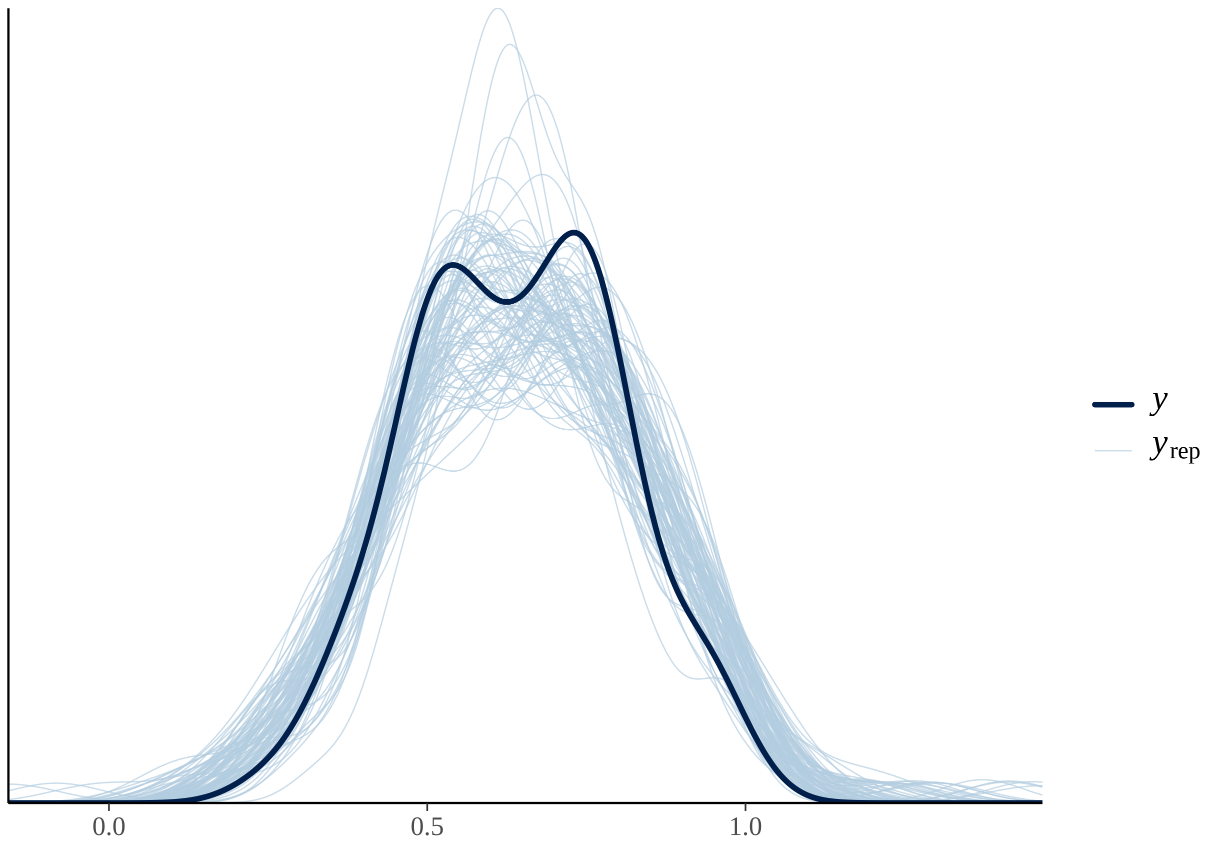

Fixed effects results TU-SH-SSH

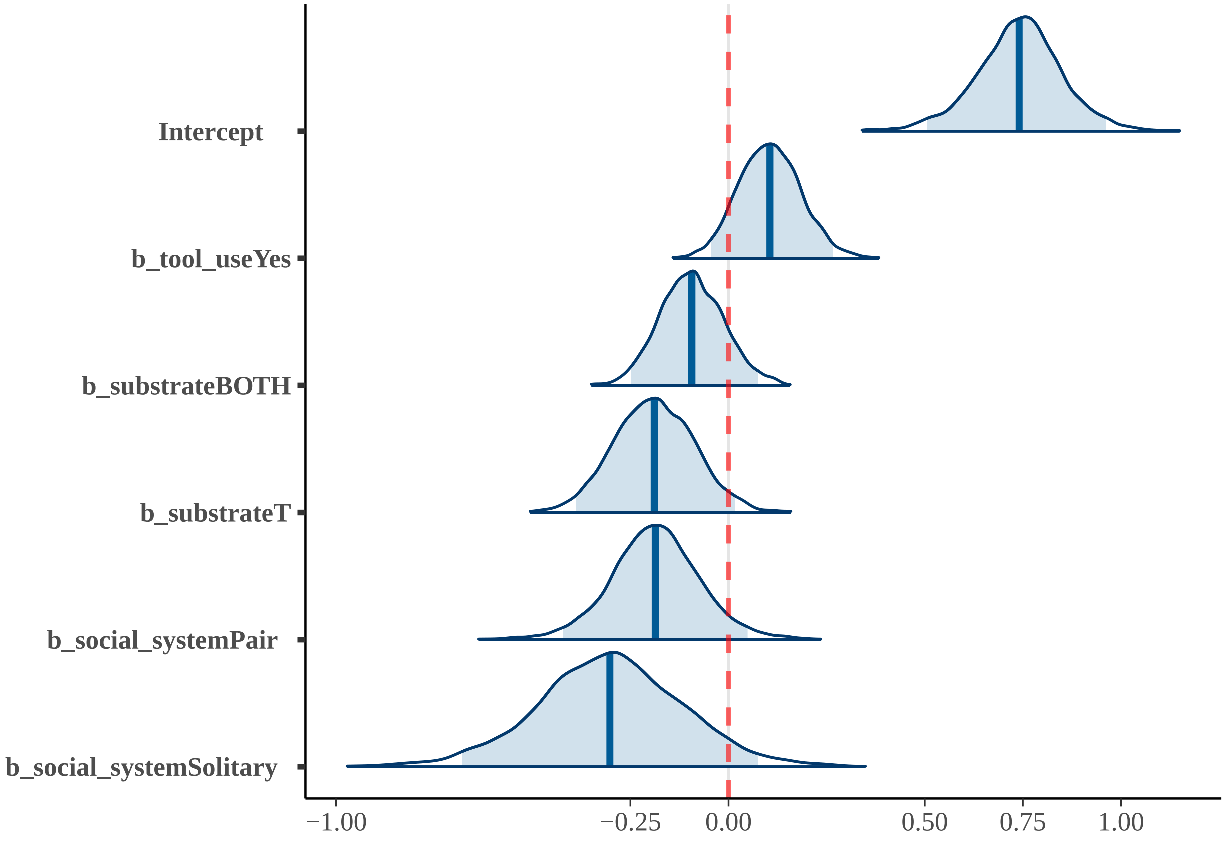

8) SP-SS-B-TUH

Summary SP-SS-B-TUH

effect	component	term	estimate	std.error	conf.low	conf.high	rhat
fixed	cond	(Intercept)	0.71	0.12	0.45	0.96	1
fixed	cond	tool_useYes	0.08	0.08	-0.08	0.24	1
fixed	cond	substrateBOTH	-0.09	0.09	-0.26	0.08	1
fixed	cond	substrateT	-0.22	0.12	-0.46	0.02	1
fixed	cond	social_systemPair	-0.09	0.18	-0.46	0.27	1
fixed	cond	social_systemSolitary	-0.10	0.24	-0.58	0.37	1
fixed	cond	bm	0.01	0.07	-0.13	0.15	1
fixed	cond	imi	-0.10	0.07	-0.24	0.05	1

effect	component	group	term	estimate	std.error	conf.low	conf.high	rhat
ran_pars	cond	obs	sd__(Intercept)	0.10	0.02	0.07	0.14	1
ran_pars	cond	phylo	sd__(Intercept)	0.03	0.01	0.01	0.06	1
ran_pars	cond	species	sd__(Intercept)	0.04	0.03	0.00	0.11	1

Posterior predictive check SP-SS-B-TUH; 100 simulated datasets

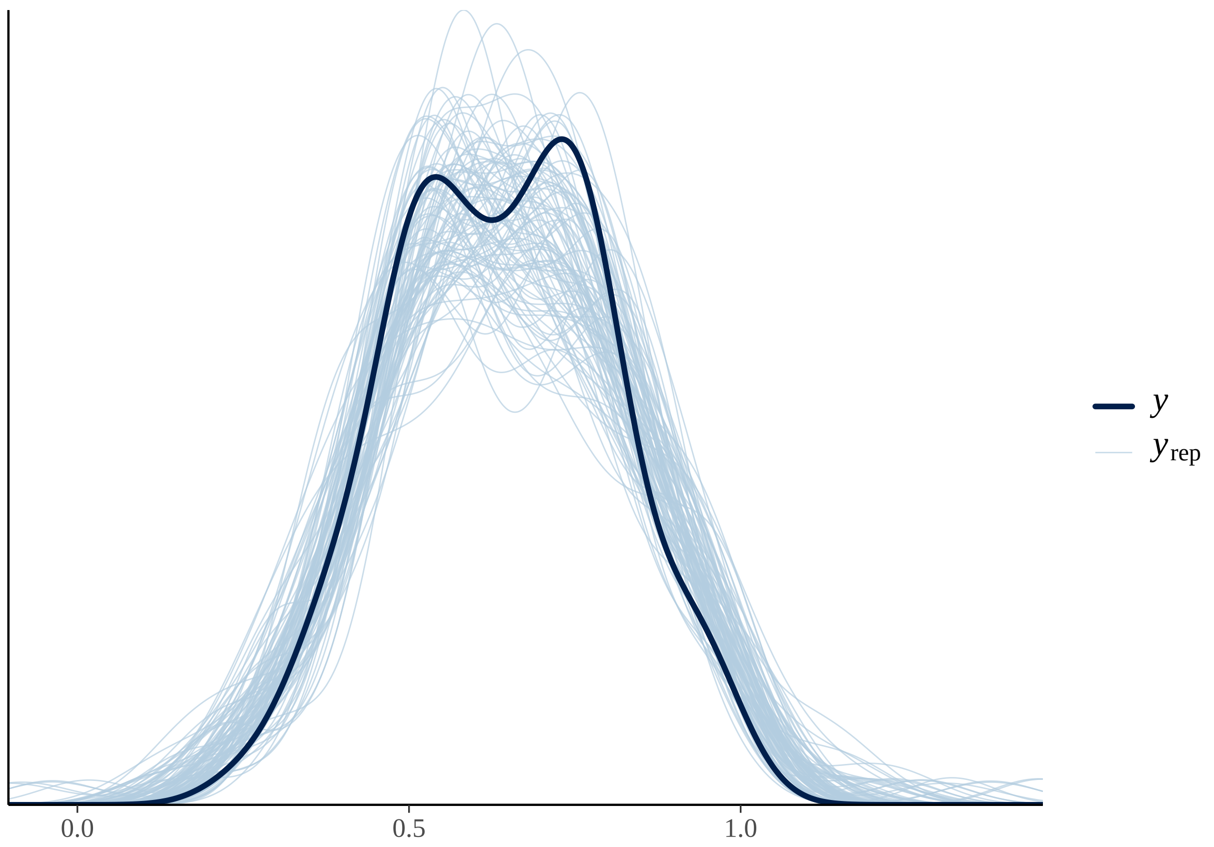

Fixed effects results SP-SS-B-TUH

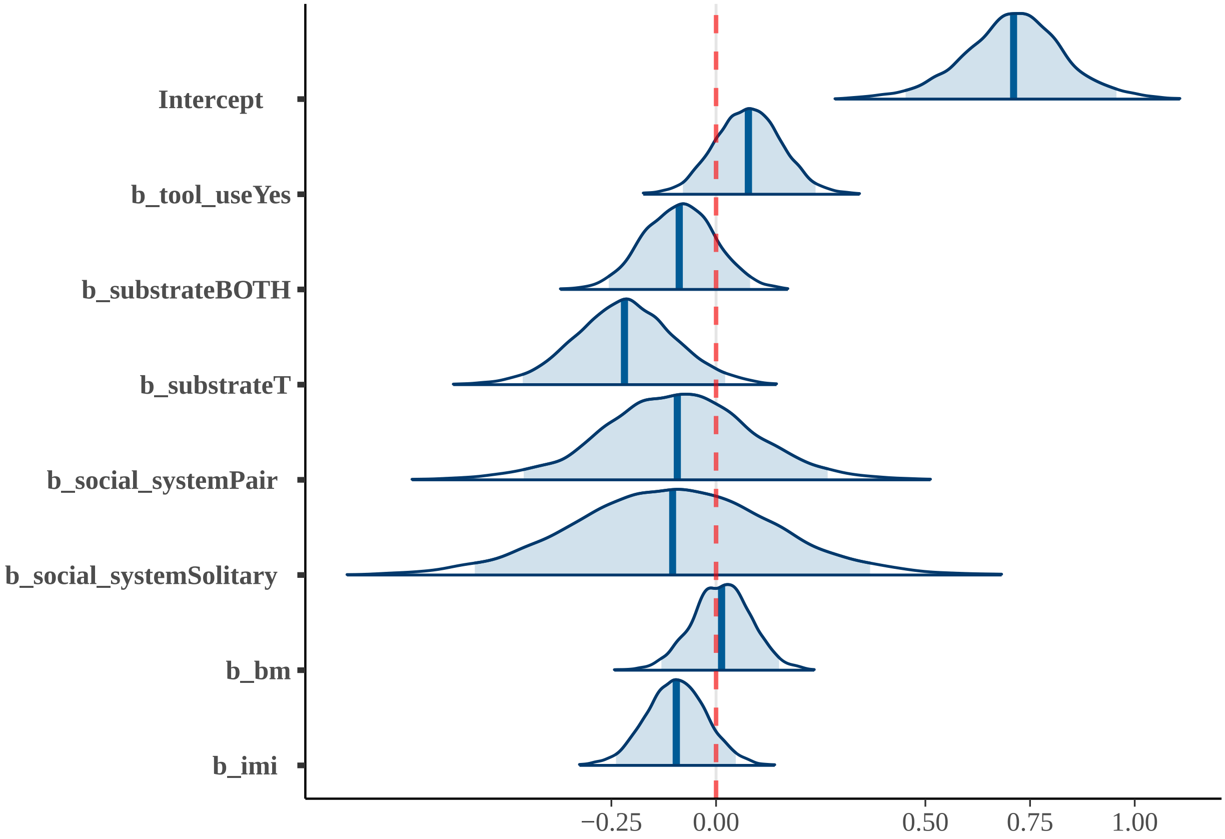

9) FH

Summary FH

effect	component	term	estimate	std.error	conf.low	conf.high	rhat
fixed	cond	(Intercept)	0.65	0.15	0.37	0.97	1
fixed	cond	dimo	-0.08	0.04	-0.16	-0.01	1
fixed	cond	cl2	-0.03	0.13	-0.31	0.23	1
fixed	cond	cl3	0.10	0.15	-0.21	0.38	1
fixed	cond	cl4	-0.02	0.16	-0.36	0.28	1

effect	component	group	term	estimate	std.error	conf.low	conf.high	rhat
ran_pars	cond	obs	sd__(Intercept)	0.10	0.02	0.07	0.14	1
ran_pars	cond	phylo	sd__(Intercept)	0.03	0.01	0.01	0.06	1
ran_pars	cond	species	sd__(Intercept)	0.04	0.03	0.00	0.11	1

Posterior predictive check FH; 100 simulated datasets

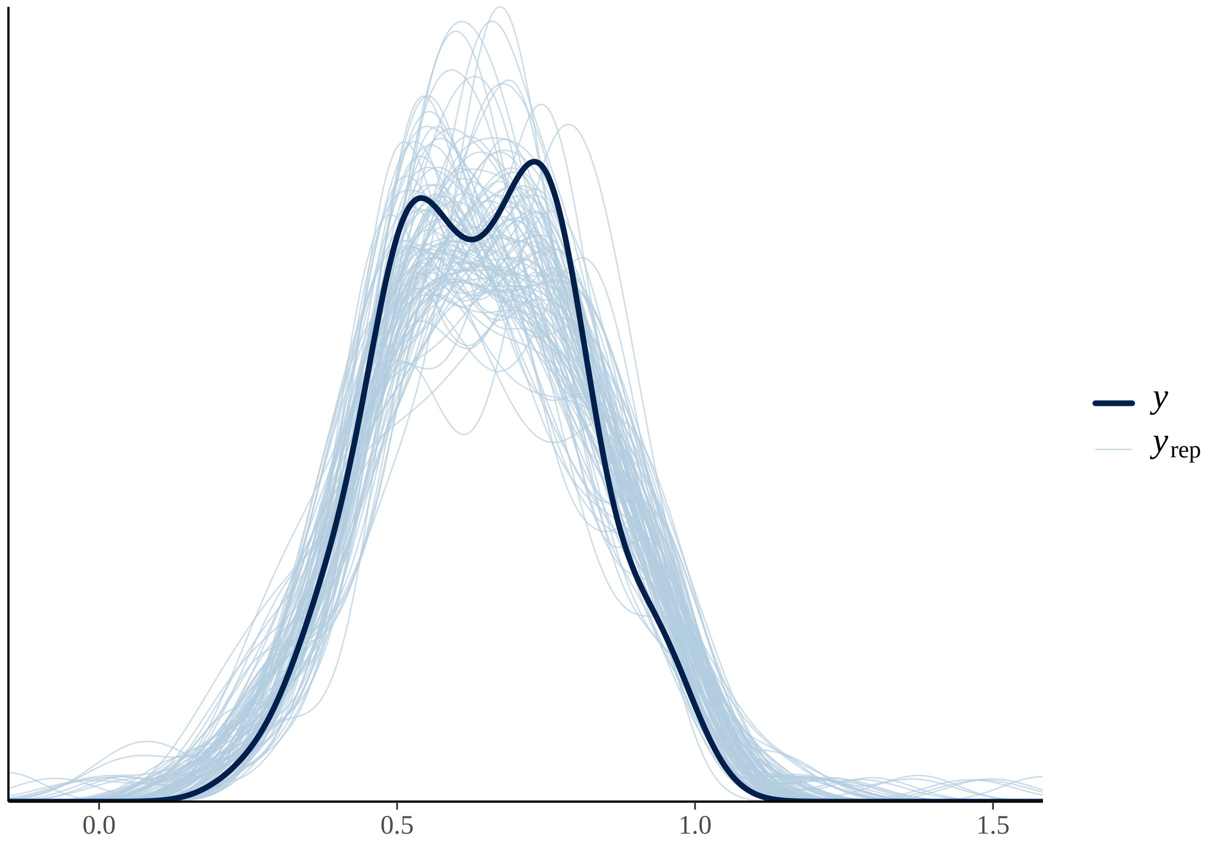

Fixed effects results FH

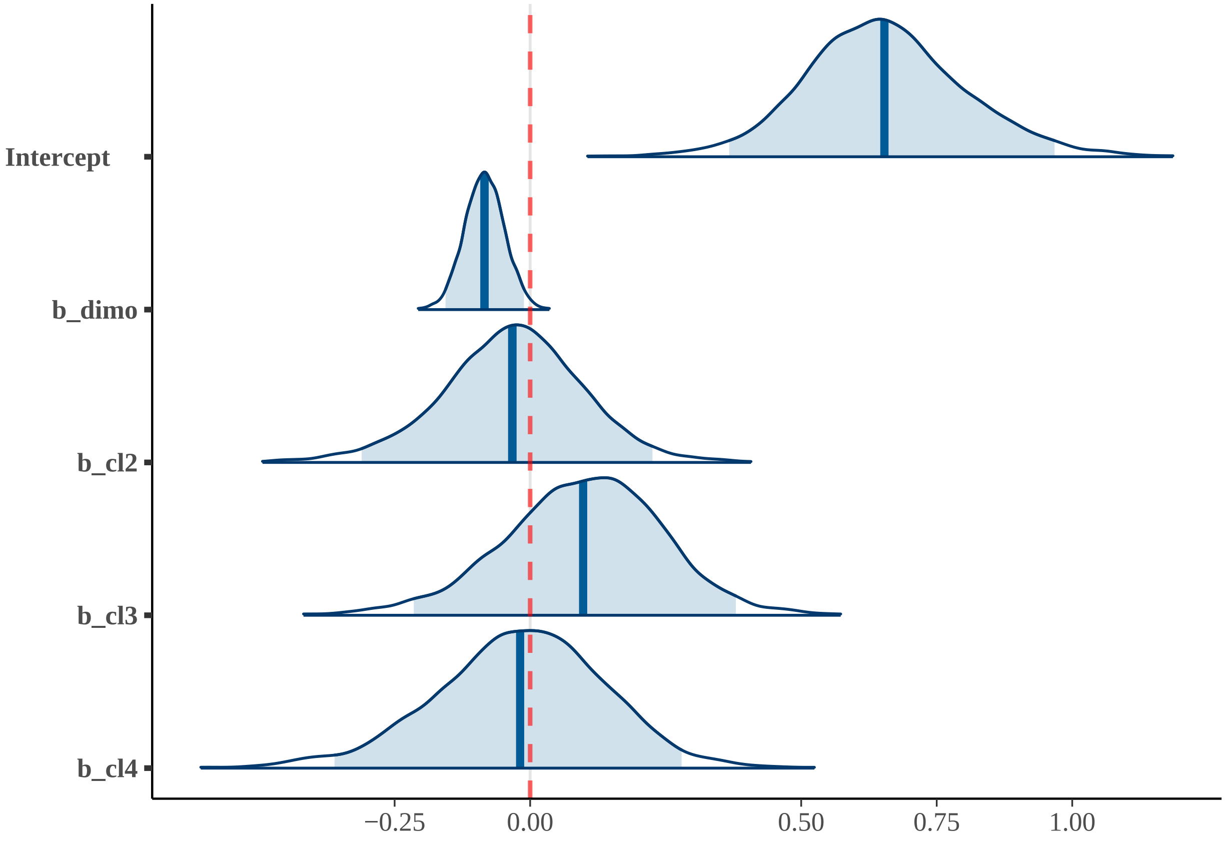

10) EFH

Summary EFH

effect	component	term	estimate	std.error	conf.low	conf.high	rhat
fixed	cond	(Intercept)	0.68	0.12	0.43	0.92	1
fixed	cond	extractive	-0.06	0.09	-0.23	0.12	1
fixed	cond	social_learning	-0.02	0.07	-0.17	0.12	1
fixed	cond	tool_useYes	0.13	0.08	-0.03	0.28	1

effect	component	group	term	estimate	std.error	conf.low	conf.high	rhat
ran_pars	cond	obs	sd__(Intercept)	0.11	0.02	0.07	0.15	1
ran_pars	cond	phylo	sd__(Intercept)	0.03	0.01	0.01	0.06	1
ran_pars	cond	species	sd__(Intercept)	0.05	0.03	0.00	0.13	1

Posterior predictive check EFH; 100 simulated datasets

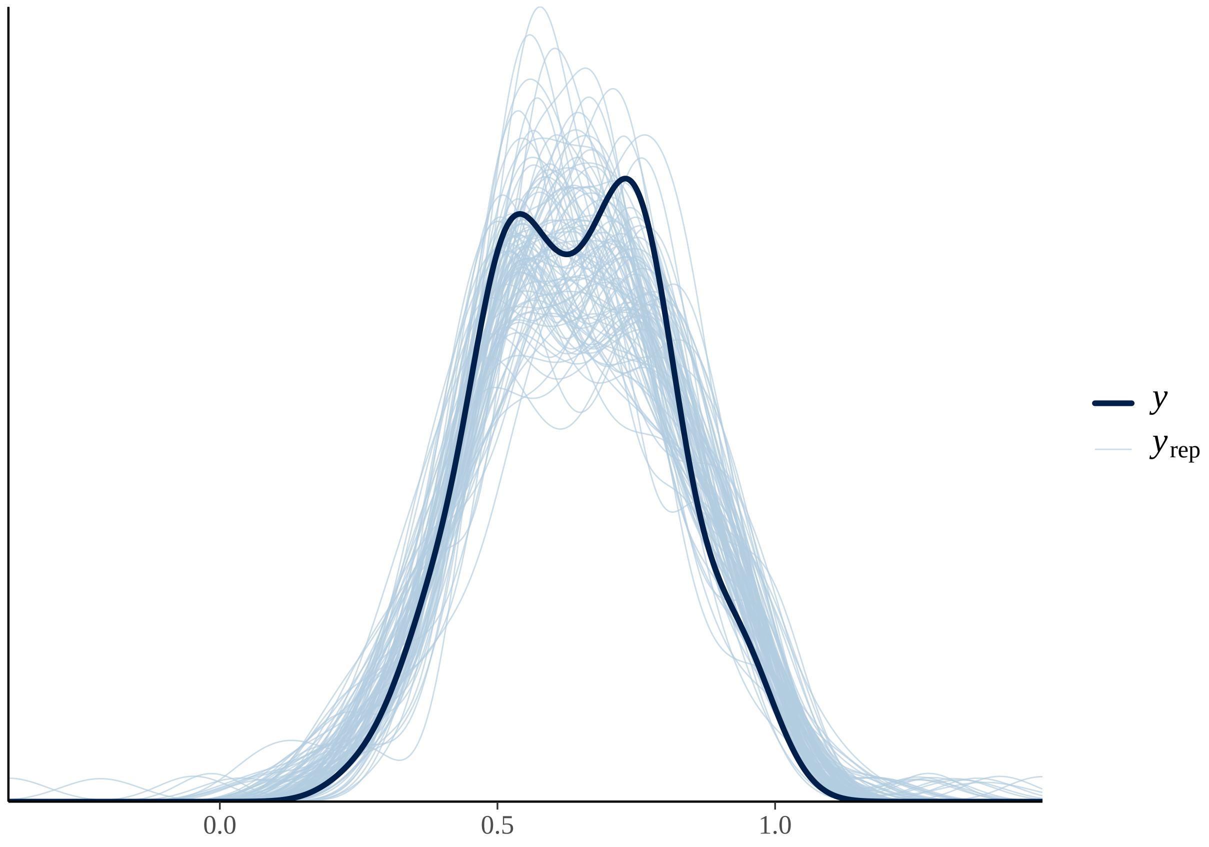

Fixed effects results EFH

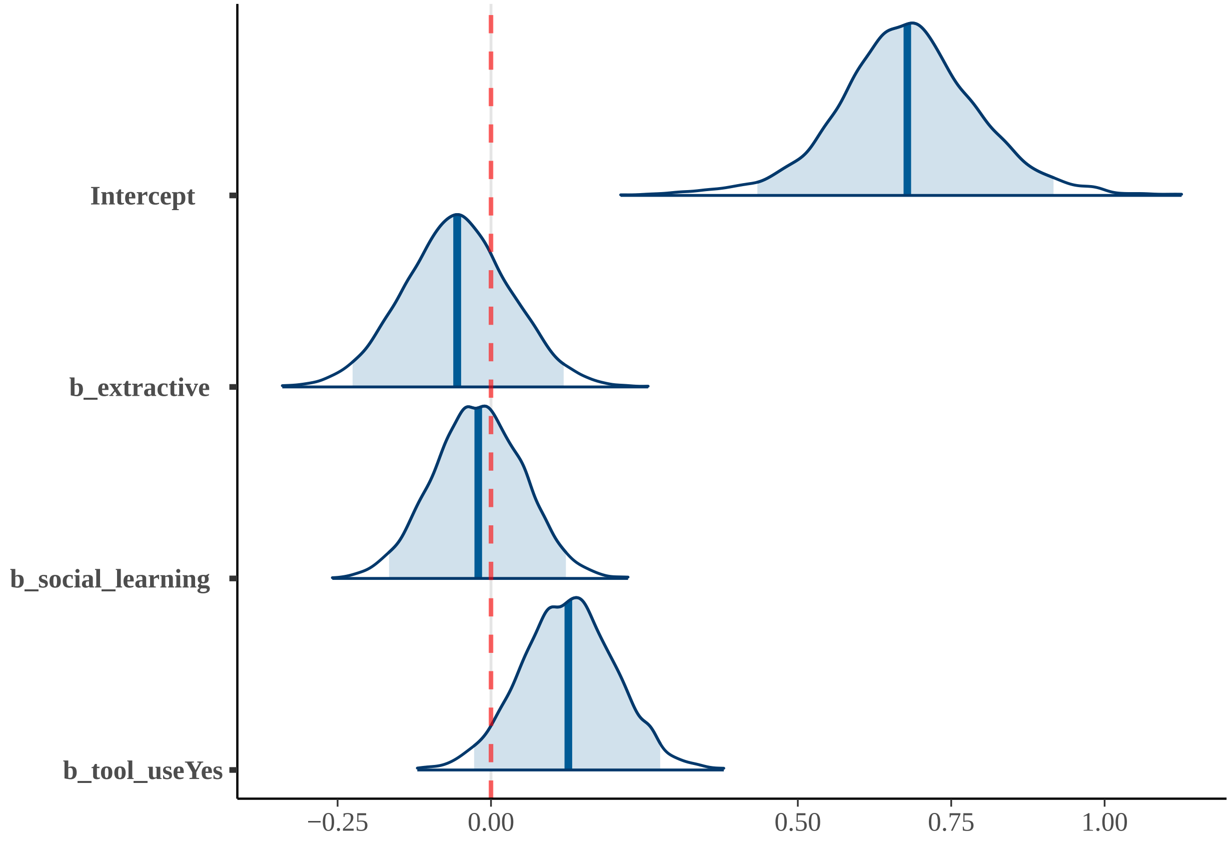

11) Reduced model

Summary Reduced model

effect	component	term	estimate	std.error	conf.low	conf.high	rhat
fixed	cond	(Intercept)	0.73	0.09	0.54	0.91	1
fixed	cond	ecv	0.09	0.04	0.01	0.16	1
fixed	cond	imi	-0.13	0.04	-0.21	-0.05	1
fixed	cond	substrateBOTH	-0.13	0.07	-0.27	0.02	1
fixed	cond	substrateT	-0.31	0.11	-0.52	-0.10	1

effect	component	group	term	estimate	std.error	conf.low	conf.high	rhat
ran_pars	cond	obs	sd__(Intercept)	0.10	0.02	0.07	0.14	1
ran_pars	cond	phylo	sd__(Intercept)	0.03	0.01	0.00	0.05	1
ran_pars	cond	species	sd__(Intercept)	0.04	0.03	0.00	0.10	1

Posterior predictive check Reduced model; 100 simulated datasets

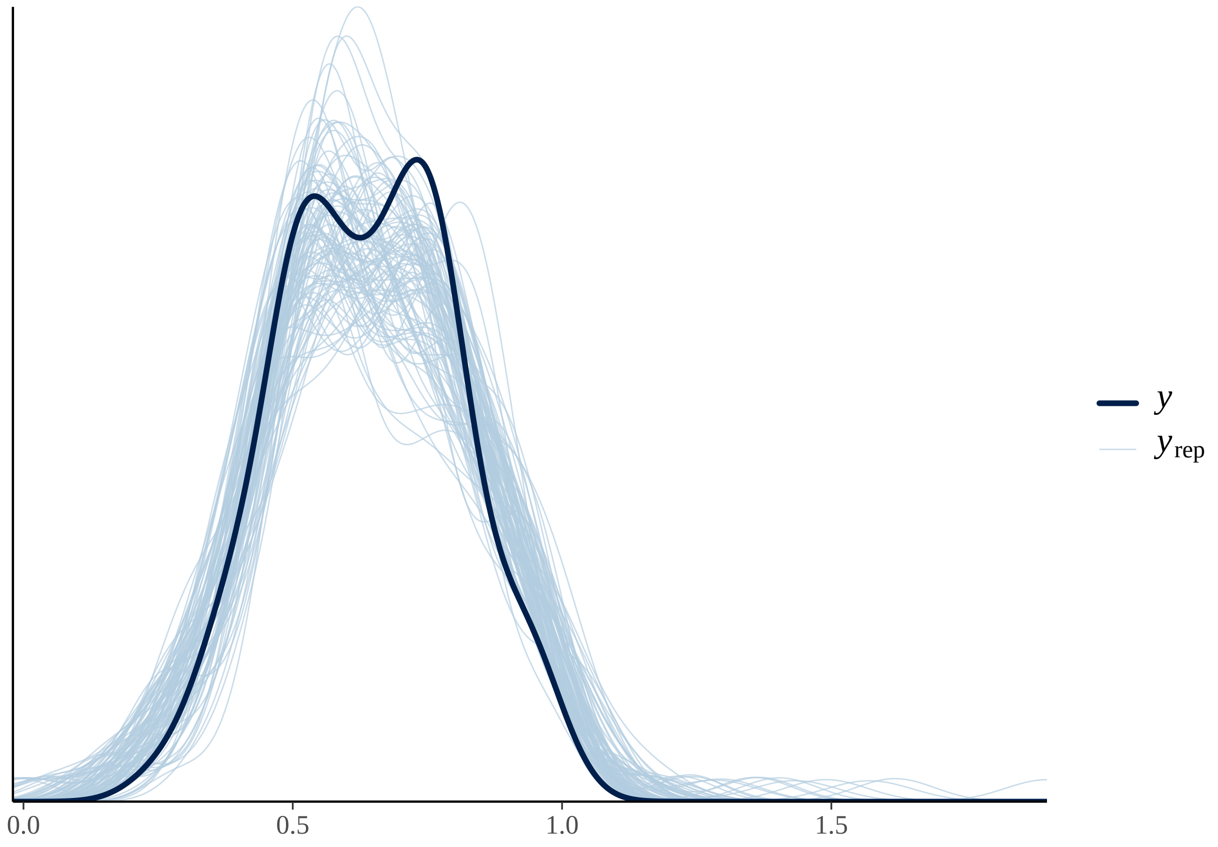

Fixed effects results Reduced model

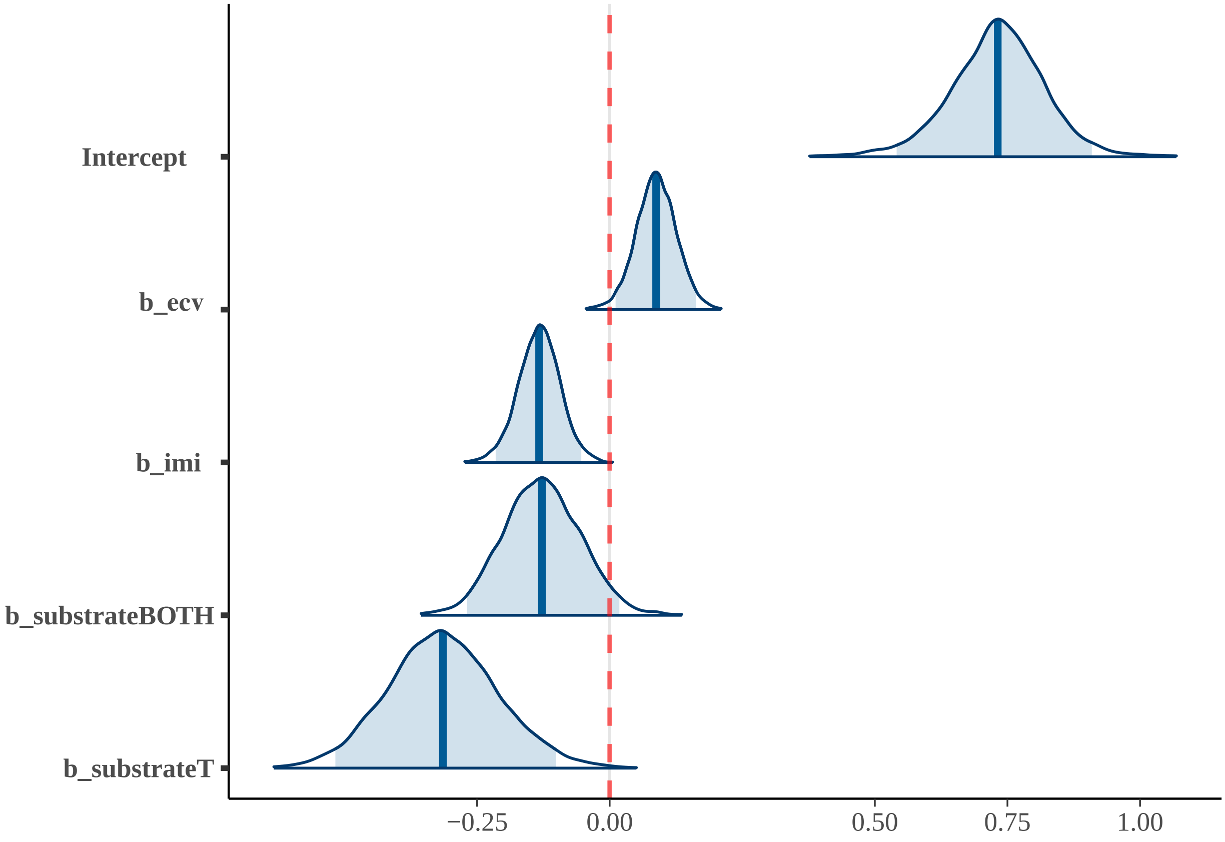

Supplement: S2 Section — (PDF) [file pbio.3003771.s007.pdf]
